# Evolutionary history of the OmpR/IIIA family of signal transduction two component systems in *Lactobacillaceae *and *Leuconostocaceae*

**DOI:** 10.1186/1471-2148-11-34

**Published:** 2011-02-01

**Authors:** Manuel Zúñiga, Ciara Luna Gómez-Escoín, Fernando González-Candelas

**Affiliations:** 1 Departamento de Biotecnología de Alimentos, Instituto de Agroquímica y Tecnología de Alimentos, Consejo Superior de Investigaciones Científicas (CSIC), PO Box 73, 46100 Burjassot, Valencia, Spain; 2 Instituto Cavanilles de Biodiversidad y Biología Evolutiva, Universidad de Valencia, Valencia, Spain; 3 Area de Genómica y Salud, Centro Superior de Investigación en Salud Pública, Valencia. Spain

## Abstract

**Background:**

Two component systems (TCS) are signal transduction pathways which typically consist of a sensor histidine kinase (HK) and a response regulator (RR). In this study, we have analyzed the evolution of TCS of the OmpR/IIIA family in *Lactobacillaceae *and *Leuconostocaceae*, two families belonging to the group of lactic acid bacteria (LAB). LAB colonize nutrient-rich environments such as foodstuffs, plant materials and the gastrointestinal tract of animals thus driving the study of this group of both basic and applied interest.

**Results:**

The genomes of 19 strains belonging to 16 different species have been analyzed. The number of TCS encoded by the strains considered in this study varied between 4 in *Lactobacillus helveticus *and 17 in *Lactobacillus casei*. The OmpR/IIIA family was the most prevalent in *Lactobacillaceae *accounting for 71% of the TCS present in this group. The phylogenetic analysis shows that no new TCS of this family has recently evolved in these *Lactobacillaceae *by either lineage-specific gene expansion or domain shuffling. Furthermore, no clear evidence of non-orthologous replacements of either RR or HK partners has been obtained, thus indicating that coevolution of cognate RR and HKs has been prevalent in *Lactobacillaceae*.

**Conclusions:**

The results obtained suggest that vertical inheritance of TCS present in the last common ancestor and lineage-specific gene losses appear as the main evolutionary forces involved in their evolution in *Lactobacillaceae*, although some HGT events cannot be ruled out. This would agree with the genomic analyses of *Lactobacillales *which show that gene losses have been a major trend in the evolution of this group.

## Background

Two component systems (TCS) are widespread signal transduction pathways mainly found in bacteria where they play a major role in adaptation to changing environmental conditions. Nevertheless, they can also be found in some eukaryotes and archaea. Numerous studies have shown the involvement of TCS in a broad range of adaptive processes such as sporulation, nitrogen regulation, phosphate regulation, cell envelope stress response, pathogenicity, motility, etc. [1]. TCS typically consist of a sensor histidine kinase (HK), usually membrane-bound, and a cytoplasmic response regulator (RR). HKs and RRs are modular proteins containing homologous and heterologous domains [2,3]. The homologous domains, kinase domain and H-box in HKs and receptor domain in RR, are involved in the phosphotransfer reaction whereas the heterologous domains, sensor (HKs) and effector (RR) domains, are involved in the reception of a specific stimulus and the corresponding response, respectively.

In the most basic scheme, upon detection of a stimulus, the HK autophosphorylates in a conserved His residue at the H-box and subsequently transfers the phosphate group to a conserved aspartyl residue at the receptor domain of the RR. Phosphorylation of the RR modulates its activity and in most cases it functions as a transcriptional regulator [1]. In addition, more complex phosphotransfer relays also exist which involve multiple phosphotransfer reactions among domains that can be found on separate polypeptides or as part of multi-domain proteins [4-6]. Furthermore, some HKs also contain PAS (*P*er-*A*rnt-*S*im) domains [7], possibly involved in sensing redox potential, HAMP domains (*H*istidine kinases, *A*denylyl cyclases, *M*ethyl binding proteins, *P*hosphatases) which have been proposed to transmit the stimulus from the sensor domain to the H-box and kinase domains [8] or a second type of His-domain termed HPt which functions as an intermediate phosphate receiver and donor in complex phosphorelays [1]. In some cases, TCS also include auxiliary proteins that regulate the activities of the HK or that influence the stability of RR phosphorylation [9].

TCS are found in varying numbers in bacteria although, generally, bacteria with larger genomes encode more TCS [10,11]. In addition, free-living bacteria usually harbour more TCS than pathogenic bacteria [4], suggesting a correlation between metabolic versatility and number of TCS [10]. Data from complete genome sequencing projects have shown that TCS-specific domains rank among the most common protein domains found in bacteria. This has led to the development of specialised databases such as MiST [12] or P2CS [13] and to the proposal of a number of classification schemes. Some researchers have based TCS classifications on phylogenetic reconstructions of conserved domains [4,14-16]. A second approach has made use of the domain composition of TCS proteins [17,18]. Notwithstanding, the results of most classifications agree to a considerable extent and have shown that the majority of TCS proteins belong to a limited number of families which share common ancestry and domain structure [19]. Furthermore, TCS are usually encoded by adjacent genes (although orphan genes can also be found) and are arranged in the same order and orientation [4].

The evolutionary history of TCS has also been the subject of a number of studies [19]. Koretke et al. [4] studied the TCS proteins encoded in 18 genomes (12 bacteria, 4 archaea and 2 eukaryotes). From their phylogenetic analyses they concluded that TCS systems originated in bacteria and were acquired by archaea and eukaryotes by multiple horizontal gene transfer (HGT) events. They also concluded that coevolution of cognate HKs and RRs has been prevalent, although some examples of recruitment were also detected, mostly in hybrid HKs. Furthermore, coevolution is also prevalent at the domain level, so that domain shuffling or swapping have been relatively rare events [4,20]. A subsequent study focused on HKs present in 207 genomes modified to some extent this view [21]. The analysis of this dataset revealed that many bacteria carry a large repertoire of recently evolved HKs as a result of lineage-specific gene expansion (LSE) or HGT and species-specific preference for either of these two modes of acquisition of new TCS. For example, genomes with large numbers of HKs relative to their genome size tended to accumulate HKs by LSE. In addition, whereas TCS acquired by HGT tended to be organized in operons, those arising from LSE were much more likely to show as "orphans" separated from their cognate RRs [21]. The origin of TCS also correlated with the frequency of subsequent gene rearrangements. For instance, whereas 47.4% of HGT-acquired HKs conserved the same domain composition, only 29.1% of LSE-acquired HKs retained the same domain structure as their closest paralogs [21].

Other studies have focused on TCS systems present in particular bacterial groups [18,22-25]. These studies have not shown great discrepancies with the conclusions from general studies although they have provided a more detailed picture of the corresponding evolutionary scenarios. For example, the study of TCS systems in *Pseudomonas *has shown a significant contribution of gene recruitment in the evolution of the NarL-group of TCS whereas coevolution was prevalent in the OmpR-group [24]. In summary, the results obtained so far indicate that all TCS share a common ancestor from which major families have evolved by duplication and divergence. This process has continued during bacterial evolution with the acquisition of new sensor or effector capabilities via domain shuffling [19].

Lactic acid bacteria (LAB) constitute a group of obligate fermentative microorganisms that produce lactic acid as the main product of sugar degradation. This characteristic has been exploited to produce a variety of fermented products since the acidification and enzymatic processes associated to their growth prevent the proliferation of detrimental organisms and pathogens and confer the characteristic flavor and texture of these products. Furthermore, some strains, especially lactobacilli that colonize the gastrointestinal tract of humans and animals, are considered as probiotics [26,27]. LAB have been isolated from a wide range of sources including a variety of foodstuffs, beverages, plants and the gastrointestinal tract of animals. Taxonomically, LAB are classified within the order *Lactobacillales *which encompasses the families *Aerococcaceae*, *Carnobacteriaceae*, *Enterococcaceae*, *Lactobacillaceae*, *Leuconostocaceae *and *Streptococcaceae*. However, phylogenetic analyses do not support the distinction between *Leuconostocaceae *and *Lactobacillaceae *[28]. For this reason, throughout this study the term *Lactobacillaceae *will be used to refer to species currently classified within the families *Lactobacillaceae *and *Leuconostocaceae*. The genome sequences of a number of *Lactobacillaceae *species from different ecological niches are currently available thus enabling comparative genomics and evolutionary analyses. An important conclusion from these studies is that lineage-specific gene loss has been extensive in the evolution of *Lactobacillales *[29]. However, no study on the evolution of TCS in this bacterial group has been carried out yet. A number of physiological studies have dealt with the functional role of TCS in LAB. These studies have shown the involvement of some TCS in quorum sensing and production of bacteriocins [30-33], the stress response in some species of this group [34-36] and malic acid metabolism in *Lactobacillus casei *[37]. These results suggest that TCS may have played a role in the adaptation of LAB to the different ecological niches that they occupy. Therefore, the phylogenetic analysis of TCS present in LAB may provide insight into the evolutionary processes involved in the adaptation of LAB to the different habitats they colonize and into the functional role of as yet uncharacterized TCS. The aim of this work is thus to explore the evolution of TCS in *Lactobacillaceae*. To this end we have focused in the OmpR/IIIA family since they are the most widely distributed in this bacterial group. The prototypic *Escherichia coli *OmpR EnvZ system was originally identified as regulating the expression of the porin-encoding genes *ompF *and *ompC *in response to medium osmolarity [38]. Later studies have shown the involvement of members of this family in varied physiological processes. To put some examples, OmpR/IIIA TCSs are involved in nitrogen metabolism in *Streptomyces coelicolor *[39] or phosphate metabolism in *E. coli *[40]and *Bacillus subtilis *[41]. Furthermore, some orthologous systems control different processes in different bacteria, such as the YycFG TCS which has been involved in cell division, cell wall biosynthesis or virulence factor expression, among other functions [42].

## Results and discussion

### Number, distribution and classification of TCS present in *Lactobacillaceae*

**Figure 1:**
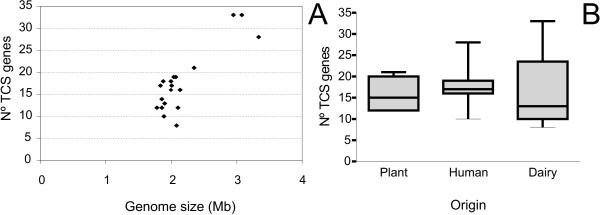
**Number of TCS-encoding genes versus genome size or habitat**. A. Number of TCS genes versus genome size in the 19 *Lactobacillaceae *strains analized. B. Number of TCS genes versus the main habitat of the corresponding strain. The upper and lower boundaries of the boxes indicate the 75^th ^and 25^th ^percentile, respectively. The line within the box marks the median. The whiskers indicate the maximum and minimum values of each data series.

The number of TCS-encoding genes harbored by the strains considered in this study varied between 8, in *Lactobacillus helveticus *DPC 4571, and 33 in *Lactobacillus casei *BL23 and *L. casei *ATCC 334 (Table 1). Taking the *Bacteria *domain as a whole, a correlation between genome size and the number of encoded TCS was observed [17]. The genomes of the *Lactobacillaceae *strains considered here have very similar genome sizes with an average of about 2 Mb, except *L. casei *and *Lactobacillus plantarum *(Table 1). Hence, this correlation cannot be observed although the strains with the largest genomes encode the highest numbers of TCS genes (Figure 1A). Additionally, no correlation was observed between the main habitat of the strains and the number of TCS genes in their genomes (Figure 1B). Several authors have observed that species with complex lifestyles, colonizing varied environments or possessing numerous alternative metabolic pathways tend to encode larger complements of signal-transducing proteins [10,21]. The lack of differences between *Lactobacillaceae *isolated from distinct environments likely reflects the low metabolic diversity within this group and their similar lifestyles and it also suggests that they do not have to cope with significantly different levels of environmental challenges.

**Table 1:** Genome size and number of TCS genes encoded by the strains used in this study

Strain	**Genome size (Mb)**^**1**^	**TCS genes**^**2**^	Origin
*Lactobacillus acidophilus *NCFM	1.99	16	Human isolate [61]
*L. brevis *ATCC 367	2.35	21	Fermented plant material [62]
*L. casei *BL23	3.08	33	Uncertain origin [63]
*L. casei *ATCC 334	2.95	33	Cheese (ATCC^3^)
*L. delbrueckii subsp. bulgaricus *ATCC 11842	1.86	12	Yogurt (ATCC)
*L. delbrueckii subsp. bulgaricus *ATCC BAA-365	1.86	14	French starter culture (ATCC)
*L. fermentum *IFO 3956	2.10	12	Fermented plant material [64]
*L. gasseri *ATCC 33323	1.89	10	Human isolate (DSMZ^4^)
*L. helveticus *DPC 4571	2.08	8	Swiss cheese isolate [65]
*L. johnsonii *NCC533	1.99	18	Human isolate [66]
*L. plantarum *WCFS1	3.34	28	Human saliva isolate [67]
*L. reuteri *DSM 20016	2.00	17	Human intestinal isolate (DSMZ)
*L. reuteri *JCM 1112	2.04	19	Human fecal isolate [64]
*L. sakei *23 K	1.88	18	Meat isolate [68]
*L. salivarius *UCC118	2.13	16	Human ileal-caecal isolate [69]
*Leuconostoc citreum *KM20	1.9	13	Kimchi [70]
*Lc. mesenteroides *subsp. *mesenteroides *ATCC 8293	2.07	19	Olive fermentation (ATCC)
*Oenococcus oeni PSU-1*	1.78	12	Wine isolate [71]
*Pediococcus pentosaceus *ATCC 25745	1.83	17	Fermented plant material [72]

^1 ^Values calculated including plasmids.

^2 ^Number of genes encoding either a RR or a HK (putative pseudogenes are not considered).

^3 ^American Type Culture Collection

^4 ^Deutsche Sammlung von Mikroorganismen und Zellkulturen

No hybrid HKs were encoded by any strain included in this study. The genes encoding HKs and their corresponding RR partners were organized in operons (not shown). In a few cases, one of the partners was a pseudogene (Table 2 and additional file 1). In addition, some true orphan genes were also detected although they accounted for a very small fraction of the total (10 genes out of 173 TCS; Table 2 and additional file 1).

**Table 2:** Number of TCS genes in different families encoded by *Lactobacillaceae*

Strain	Families						
	
	AraC	CitB	NarL	LytR	LytR	OmpR	YcbB
	I	IV	II	I	**HPK**_**10**_	IIIA	IV
*L. acidophilus *NCFM	0	0	0	0	4	12	0
*L. brevis *ATCC 367	0	2	0	0	3^1^	14	2
*L. casei *BL23	0	2	4	0	3^2^	24	0
*L. casei *ATCC 334	0	2	4	0	3^2^	24	0
*L. delbrueckii subsp. bulgaricus *ATCC 11842	0	0	0	0	1^2^	11^2^	0
*L. delbrueckii subsp. bulgaricus *ATCC BAA-365	0	0	0	0	2	12	0
*L. fermentum *IFO 3956	0	0	2	0	0	9^3^	1^3^
*L. gasseri *ATCC 33323	0	0	0	0	0	10	0
*L. helveticus *DPC 4571	0	0	0	0	0	8	0
*L. johnsonii *NCC533	0	0	2	0	4	12	0
*L. plantarum *WCFS1	2	0	4	0	10^3,4^	12	0
*L. reuteri *DSM 20016	0	0	1^3^	2	0	13^3^	1^3^
*L. reuteri *JCM 1112	0	0	3^3^	2	0	13^3^	1^3^
*L. sakei *23 K	0	0	3^2^	0	3^2^	12	0
*L. salivarius *UCC118	0	0	2	0	4	10	0
*Leuconostoc citreum *KM20	0	0	0	0	1^2^	12	0
*Lc. mesenteroides *subsp. *mesenteroides *ATCC 8293	0	0	2	0	4	13^2^	0
*Oenococcus oeni *PSU-1	0	0	0	2	0	10	0
*Pediococcus pentosaceus *ATCC 25745	0	0	2	2	2	10	1^3^

**Total**	**2**	**6**	**29**	**8**	**44**	**241**	**6**

^1 ^Gene cluster containing one RR and two HKs.

^2 ^One incomplete TCS (pseudogene).

^3 ^One incomplete TCS (orphan).

^4 ^One gene cluster containing two RR and one HK.

The TCS present in *Lactobacillaceae *were classified according to the schemes of Fabret et al. [15] for HKs and Galperin [17] for RRs. The classification of HKs is based on the comparison of the amino-acid sequence of the region around the phosphorylatable histidine [15]. This analysis divided the HKs present in *B. subtilis *into five classes (I, II, IIIA, IIIB and IV). The classification of RRs is based primarily on their domain architectures and structures of the constituent domains [17]. Most HKs and RRs could be accommodated within these classification schemes. The only exceptions corresponded to a group of HKs associated to LytR RRs, which correspond to the HPK_10 _family of the classification of Grebe and Stock [14], and a group of RRs homologous to the *E. coli *CitB not included in Galperin's classification [17]. A strong correlation in the association of families of HKs and RR was observed in *Lactobacillaceae*, for example, IIIA HKs are invariably associated to OmpR RRs. This correlation has been previously pointed out as a common feature of TCS [4,14,15] and led to Grebe and Stock to propose that many HKs and their cognate RRs have evolved as integral units [14], a view in agreement with the coevolution model [4].

A summary of the types of TCS found in each strain is shown in Table 2 and detailed lists of TCS identified in each strain are provided in the additional file 1. By far, the OmpR/IIIA family was the most prevalent in *Lactobacillaceae*, accounting for 71% of the TCS present in this group (Table 2). Furthermore, this is the only family present in all the strains included in this study. For these reasons, we focused our attention in this family for subsequent analyses.

### Identification and analysis of clusters of orthologs in the OmpR/IIIA family of TCS

Preliminary identification of clusters of orthologs of RR and HK sequences was performed by creating an orthology table of the 19 genomes used in this study using the clustering algorithm implemented in MBGD [43] and manually checking the clusters of orthologs thus obtained for each previously identified TCS gene. The clusters were named according to the following criteria: when a putative ortholog with characterized function was identified, the cluster was named after this ortholog; if no functionally characterized ortholog was found, the group was named after the locus tag of a representative sequence of the cluster. The clusters of orthologs are listed in Table 3.

**Table 3:** Number of TCS in the different clusters of orthologs of the OmpR/IIIA family encoded by *Lactobacillaceae*

Strain	Clusters of orthologs									
	
	Bce	Bil	Cia	Cro	Eta	Kin	Pho	Ycl1	Ycl2	Yyc
*L. acidophilus *NCFM		1		1	1	1		1		1
*L. brevis *ATCC 367			1	1	1	1	1	1		1
*L. casei *BL23	2		1	1	1	1	1	1		1
*L. casei *ATCC 334	2		1	1	1	1	1	1		1
*L. delbrueckii subsp. bulgaricus *ATCC 11842		1		1	1			1		1
*L. delbrueckii subsp. bulgaricus *ATCC BAA-365		1		1	1			1		1
*L. fermentum *IFO 3956				1	1		1^1^	1		1
*L. gasseri *ATCC 33323		1		1	1			1		1
*L. helveticus *DPC 4571		1		1	1			1		1
*L. johnsonii *NCC533				1	1	1		1		1
*L. plantarum *WCFS1				1	1	1	1	1		1
*L. reuteri *DSM 20016	1	1		1	1		1^1^	1		1
*L. reuteri *JCM 1112	1	1		1	1		1^1^	1		1
*L. sakei *23 K	1			1	1		1	1		1
*L. salivarius *UCC118				1	1		1	1		1
*Leuconostoc citreum *KM20	1		1	1	1		1			1
*Lc. mesenteroides *subsp. *mesenteroides *ATCC 8293	1		1	1	1		1		1	1
*Oenococcus oeni *PSU-1			1	1		1			1	1
*Pediococcus pentosaceus *ATCC 25745				1	1		1	1		1

^1 ^One incomplete TCS (orphan).

A phylogenetic reconstruction was performed in order to investigate the evolutionary relationships of the clusters identified in MBGD. *Lactobacillaceae *sequences and selected outgroup sequences (see Methods) were aligned with Muscle and the alignments subsequently refined with Gblocks. The resulting datasets consisted in 147 sequences with 96 conserved positions for the HK alignment and 149 sequences and 158 conserved positions for the RR alignment (additional file 2).

ProtTest was used to determine the best fit model of amino acid substitution. Model LG [44] with a discrete gamma distribution to account for heterogeneity in evolutionary rates among sites, an estimation of the proportion of invariant sites and the empirical frequencies of amino acids (LG+G+I+F) was identified as the best fit model for both datasets. The phylogenetic information content of the datasets was then evaluated by using likelihood mapping. Briefly, this analysis enables to estimate the suitability for phylogenetic reconstruction of a data set from the proportion of unresolved quartets in a maximum likelihood analysis. The analysis was carried out using TreePuzzle with the WAG [45] model of substitution (the second best model selected by ProtTest) since the LG model is not implemented in this program. On the basis of ProtTest results, the datasets were analysed with a discrete gamma distribution and the empirical amino acids frequencies (WAG+G+F). The likelihood mapping showed that both datasets contained relatively low phylogenetic information, with only 68.2% and 77.7% fully resolved quartets in HKs and RRs, respectively (Fig. S1 in additional file 3).

**Figure 2:**
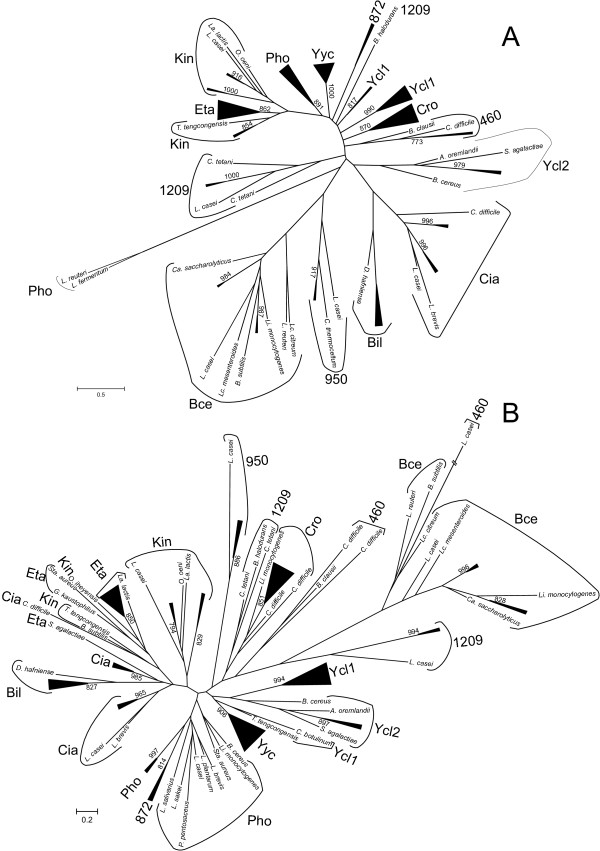
**Summarized maximum likelihood topology of the OmpR and IIIA sequences used in this study**. A. Topology of OmpR (RR) sequences. B. Topology of IIIA (HK) sequences used in this study. The complete trees are shown in Fig. S2 in additional file 3. Support values for the bootstrap analysis by maximum likelihood with support values higher than 750 (1000 bootstrap replicates). The clusters of orthologs derived from the analysis are indicated. The length of the *Lactobacillus casei *460 HK branch has been shortened. Additional details are provided in additional file 1 and Fig. S2 in additional file 2.

The phylogenetic reconstructions were performed with PhyML using the LG+G+I+F model (Figure 2 and Fig. S2 in additional file 3). In accordance with the results of the likelihood mapping, very few nodes had bootstrap support values higher than 75%. Most clusters of orthologs identified in MBGD could be distinguished in the RR tree, although some of them were not supported (clusters 950, Bce, Cia and Ycl2), and in other groups some outgroup sequences did not cluster with their corresponding *Lactobacillaceae *counterparts (clusters 1209, Kin and Ycl1; see Figure 2 and Fig. S2 in additional file 3). Furthermore, the orphan RRs Lreu_1569 and LAF_1230 encoded by *Lactobacillus reuteri *and *Lactobacillus fermentum*, respectively, constituted a separate cluster (Figure 2 and Fig. S2 in additional file 3). However, these genes were located next to a gene cluster encoding a putative phosphate uptake system homologous to those located next to Pho TCS (Fig. S3 in additional file 3).

The HK tree was less resolved, as expected from the likelihood mapping result, and in many cases outgroup sequences did not cluster with their corresponding *Lactobacillaceae *counterparts. Furthermore, some clusters were not observed in the HK phylogenetic reconstruction. HKs belonging to clusters Pho and 872 constituted one cluster (although with low support in their basal nodes; Figure 2). HKs belonging to clusters Ycl1 and Ycl2 were identified by MBGD as belonging to the same cluster of orthologs and the phylogenetic analysis also suggested a relationship between these two clusters. However, the phylogenetic reconstruction and MBGD clustering indicated that Ycl1 and Ycl2 RRs constituted separate clusters of orthologs.

In order to determine whether the above mentioned incongruent cases were due to the low resolution of the trees or they indicated wrong assignments of clusters of orthologs, detailed analyses of Ycl1 and Ycl2 HKs, Pho and 872 RRs and HKs, and Eta and Kin RRs and HKs were carried out.

**Figure 3:**
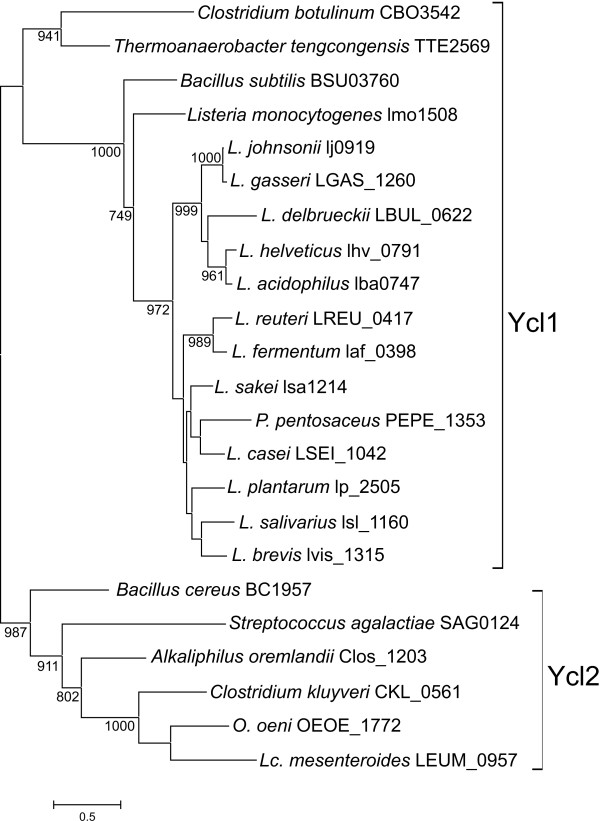
**Maximum likelihood topology of the Ycl1 and Ycl2 HK sequences used in this study**. The tree is arbitrarily rooted with the Ycl2 cluster. The species and the locus tags of the corresponding genes are indicated. The brackets indicate the clusters of orthologs. Support of nodes is indicated as in Figure 2.

HK sequences belonging to groups Ycl1 and Ycl2 were aligned, resulting in a dataset of 233 sites after trimming the initial alignment with Gblocks (additional file 2). The best fit model for this dataset was LG+G+I+F. The likelihood mapping (using again WAG+G+F) showed an increase in phylogenetic signal compared to the complete HK dataset (89% resolved quartets; Fig. S4 in additional file 3). The phylogenetic analysis of Ycl1 and Ycl2 HKs showed that Ycl1 and Ycl2 formed separate clusters with strong support that included their corresponding outgroup sequences (Figure 3) with the exception of the putative Ycl1 sequences of *Clostridium botulinum *and *Thermoanaerobacter tengcongensis*. This result confirms that they constitute two different clusters of orthologs.

**Figure 4:**
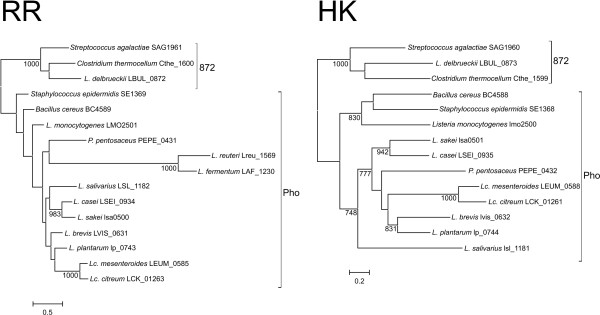
**Maximum likelihood topologies of the Pho and 872 sequences used in this study**. The trees are arbitrarily rooted with the 872 cluster. The species and the locus tags of the corresponding genes are indicated. The brackets indicate the clusters of orthologs. Support of nodes is indicated as in Figure 2.

Pho and 872 RRs and HKs were aligned and trimmed, resulting in 193 and 239 site datasets, respectively (additional file 2). ProtTest analysis also selected LG+G+I+F as the best fit model for both datasets. Likelihood mapping analysis also showed an increase in phylogenetic signal in the HK dataset (85.5% resolved quartets; Fig. S4 in additional file 3) but the phylogenetic signal in the RR dataset was slightly lower than in the complete OmpR dataset (73.3% resolved quartets for Pho and 872 vs. 77.7% for the OmpR dataset; Fig. S4 in additional file 3). The phylogenetic reconstruction of Pho and 872 HKs (Figure 4) separated both groups, thus confirming that they constitute separate clusters of orthologs. The phylogenetic reconstruction of Pho RR also showed the separation between Pho and 872 clusters. Furthermore, the orphan genes Lreu_1569 and LAF_1230 appeared in a long branch within the other Pho sequences (Figure 4). Although the basal nodes were not supported in the maximum likelihood reconstruction, the position of these two sequences in the phylogenetic tree and the analysis of their genomic context (Fig. S3 in additional file 3) strongly suggest that they belong to the Pho cluster of orthologs.

**Figure 5:**
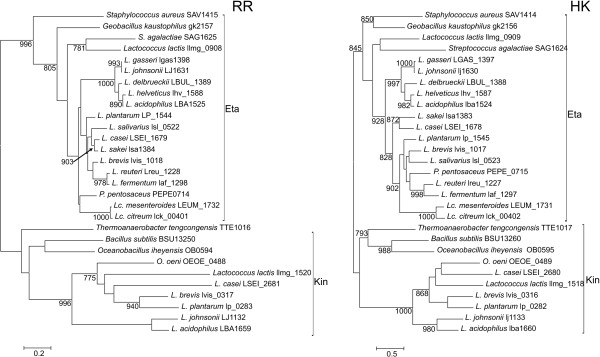
**Maximum likelihood topologies of the Eta and Kin sequences used in this study**. The trees are arbitrarily rooted with the Kin cluster. The species and the locus tags of the corresponding genes are indicated. The brackets indicate the clusters of orthologs. Support of nodes is indicated as in Figure 2.

Eta and Kin sequences were also identified as separate clusters of orthologs; however, the phylogenetic reconstructions of RR and HKs suggested that they might constitute a cluster of orthologs. In order to ascertain this point a detailed analysis of these groups was also carried out. The trimmed alignments of the corresponding HK and RR sequences consisted of 262 and 203 conserved sites, respectively (additional file 2). ProtTest selected LG+G+I+F for the HK dataset and LG+G for the RR dataset. The likelihood mapping analysis (using WAG+G+F) showed an increase in phylogenetic signal for both datasets (85% and 89.1% resolved quartets for HK and RRs, respectively; Fig. S4 in additional file 3). The ML reconstruction showed that Eta and Kin sequences were clearly separated with strong support, thus demonstrating that they constitute separate clusters of orthologs (Figure 5).

In summary, the phylogenetic reconstructions of OmpR RRs and IIIA HKs showed the clustering of the *Lactobacillaceae *orthologous sequences with their corresponding outgroup sequences thus indicating that the TCS systems present in *Lactobacillaceae *have not resulted from duplications (lineage-specific gene expansion) after the differentiation of this taxonomical group. This result suggests that these systems either were present in the last common ancestor of the group or that they were acquired by HGT during the evolution of this group.

### Distribution of clusters of orthologs in the reference tree

**Figure 6:**
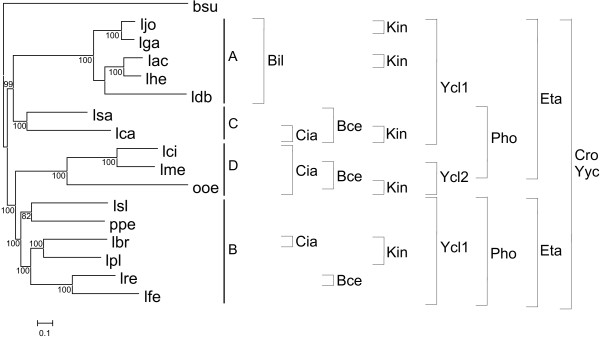
**Distribution of the OmpR/IIIA clusters of orthologs identified in *Lactobacillaceae *in a reference phylogenetic tree**. A-D indicate the subgroups identified by Claesson et al. [28]. The brackets indicate the species harboring TCSs belonging to each of the clusters of orthologs identified in *Lactobacillaceae*. Support of nodes is indicated as in Figure 2. bsu, *Bacillus subtilis*; lac, *Lactobacillus acidophilus*; lbr, *Lactobacillus brevis*; lca, *Lactobacillus casei*; ldb, *Lactobacillus delbrueckii*; lfe, *Lactobacillus fermentum*; lga, *Lactobacillus gasseri*; lhe, *Lactobacillus helveticus*; ljo, *Lactobacillus johnsonii*; lpl, *Lactobacillus plantarum*; lre, *Lactobacillus reuteri*; lsa, *Lactobacillus sakei*; lsl, *Lactobacillus salivarius*; lci, *Leuconostoc citreum*; lme, *Leuconostoc mesenteroides*; ooe, *Oenococcus oeni*; ppe, *Pediococcus pentosaceous*.

In order to gain insight on the origin of the OmprR/IIIA TCS present in *Lactobacillaceae*, we compared their distribution with a concatenated reference species tree (Figure 6). The reference tree was derived from a 139204 sites dataset obtained from the Gblocks-trimmed concatenated alignments of 141 genes (see Methods). The tree was obtained by maximum likelihood using the (GTR+G+I+F) nucleotide substitution model [46] selected with jModelTest. The topology of the tree was essentially the same as that obtained by Claesson et al. [28] and the four groups identified by these authors were also identified in this phylogenetic reconstruction (Figure 6).

Clusters of orthologs with only one *Lactobacillaceae *sequence were not considered, as this analysis cannot provide clues about their origin. The widespread distribution of clusters Cro, Eta (only absent in *Oenococcus oeni*), and Yyc strongly suggests that they were present in the last common ancestor of *Lactobacillaceae*. Similarly, the distribution of Pho can be explained by lineage-specific gene losses in the last common ancestor of group A and in *O. oeni*. Alternative scenarios would require three independent HGT events in the last common ancestor of group B, the last common ancestor of group C, and the last common ancestor of *Leuconostoc mesenteroides *and *Leuconostoc citreum *or two HGT events in the last common ancestors of group C and groups B and D and a subsequent lineage-specific gene loss in *O. oeni*. The distribution of the Ycl1 cluster also points to its presence in the last common ancestor of *Lactobacillaceae*, with a subsequent lineage-specific gene loss in group D. The origin of other clusters is more controversial: the distribution of Kin sequences could be explained by five HGT events or seven lineage-specific gene losses; the distribution of Cia by three HGT events or six lineage-specific gene losses; the distribution of Bce by four HGT events or five lineage-specific gene losses, and, the distribution of Bil by one HGT or two lineage-specific gene losses. Although future analyses with more sequences may shed light on the phylogenetic history of these clusters, it is worth mentioning that if they had resulted from HGT events these must have occurred long ago, because clearly orthologous genes are shared by distantly related strains within the *Lactobacillaceae*.

### Phylogenetic analyses of Cro, Eta and Yyc clusters of orthologs

As we have just seen, most TCS of the OmpR/IIIA family have a limited distribution in *Lactobacillaceae *(Table 3) making it difficult to obtain reliable information about their evolutionary history. Only two systems, Cro and Yyc are present in all the strains used in this study. In addition, Eta TCS is also present in all the strains except *O. oeni*. Hence, we selected these three systems to further analyze two points. Firstly, we were interested on the relative roles of coevolution and gene recruitment in the evolution of the OmpR/IIIA family in *Lactobacillaceae*. Secondly, we wanted to determine whether vertical inheritance could explain the phylogenetic relationships of the OmpR/IIIA TCS.

**Figure 7:**
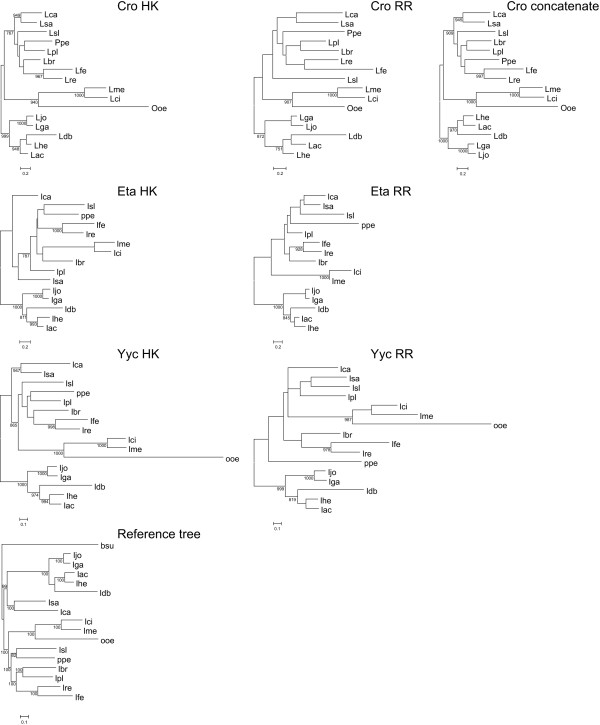
**Maximum likelihood topologies of the Cro, Eta, Yyc and the concatenated reference sequences used in this study**. The trees are arbitrarily rooted with the A subgroup of *Lactobacillaceae *species. Support of nodes is indicated as in Figure 2. Abbreviations of bacterial names are used as indicated in Figure 6.

For this purpose, the nucleotide sequences of the genes encoding the RR and HK of the Cro, Eta and Yyc clusters were aligned resulting in datasets of 684 and 1011 (RR and HK, respectively) sites for Cro, 678 and 1041 for Eta, and, 693 and 1752 for Yyc. The GTR+G+I+F was identified as the best substitution model by jModelTest. Likelihood mapping showed limited phylogenetic signal, especially in the RR datasets (70.4%, 76.5% and 72.9% resolved quartets for Cro, Eta and Yyc RR datasets, respectively; 83%, 79.9% and 83.3% for the HK datasets; Fig. S5 in additional file 3). The phylogenetic reconstructions of HKs and RRs (Figure 7) showed, in accordance with the likelihood mapping results, that only a few nodes of the phylogenetic tree had support values higher than 75%. Comparisons between both trees and the reference tree were evaluated with the Shimodaira-Hasegawa test (SH; see Methods) to determine whether the likelihood of the data associated to each tree was significantly different at an alpha level of 0.05 (a value above the threshold indicating a non-significant difference).

The analysis of Cro sequences showed that the HK dataset rejected the topologies of the reference and the RR tree (p = 0.047 and p = 0.026, respectively) whereas the RR dataset did not reject any of the two other topologies (p = 0.317 and p = 0.18 for the reference tree and the HK tree, respectively). This discrepancy could be partly due to the low resolution of the trees. Therefore, a concatenated alignment of the HK and RR datasets was built in order to increase the phylogenetic signal. The likelihood mapping of the concatenated alignment (Fig. S5 in additional file 3) showed an increase in the phylogenetic signal of the dataset (86.9% resolved quartets) compared to the HK and RR cognate datasets. The phylogenetic reconstruction obtained with the concatenated dataset was similar to that obtained with the HK dataset (although the positions of *Lactobacillus brevis*, *Lactobacillus delbrueckii *subsp. *bulgaricus *and *Pediococcus pentosaceus *changed; see Figure 7). The Shimodaira-Hasegawa test of the concatenated dataset showed that this dataset did not reject the reference, HK or RR topologies (p = 0.089, p = 0.663 and p = 0.297, respectively). Considering that the concatenated alignment included the phylogenetic signal of the HK and RR datasets and that both topologies were not rejected by the SH test, we concluded that both genes share the same evolutionary history in *Lactobacillaceae *and, given that the reference topology was not rejected either, that vertical inheritance can explain the evolution of this TCS within this group.

The analyses of the Eta datasets showed that the HK dataset rejected the RR topology but not the reference topology (p = 0.041 and p = 0.386, respectively). On the contrary the RR dataset rejected both the reference topology and the HK (p = 0.014 and p = 0.008). A more detailed examination of the two topologies revealed that group A in the reference tree (Figure 6) was also found in the HK and RR trees for the Eta datasets, where it was recovered with 100% bootstrap support (Figure 7). However, the relationships among the other three groups changed quite dramatically. Group D still appeared in the two trees, but it was no longer a sister group to group B for the HK sequences and it clustered within them. This makes group B to be paraphyletic for HK. Furthermore, group C sequences did not group in the HK tree and appeared at the base of a B/D clade. A similar case occurred for the RR tree, in which group B was paraphyletic due to the inclusion of group C sequences. Since the RR dataset rejected both the HK and the reference topologies, it can be hypothesized that some evolutionary events, apart from vertical inheritance, occurred during the evolutionary history of this cluster. However, the possibility that these sequences do not hold enough phylogenetic signal for deriving their true relationships cannot be ruled out and in order to derive reliable conclusions more sequences will be necessary.

For Yyc sequences, the comparison of the HK dataset with the RR and the reference tree showed that whereas the topology of the RR tree was rejected (p = 0.000) the topology of the reference tree was not significantly different (p = 0.466). On the other hand, the RR dataset did not reject the HK topology (p = 0.064) nor that of the reference tree (p = 0.111). Taking into account the low resolution of the RR tree the results of these tests indicate that there are no significant differences between the topologies obtained with the two datasets and that these topologies are not significantly different to that obtained with the reference tree. We conclude therefore that both genes share the same evolutionary history and that vertical inheritance explains the phylogenetic relationships between the different sequences.

In summary, the analyses of the evolutionary history of these three TCS in this bacterial group do not provide evidence against a parallel evolution of the two genes, with no signs of gene recruitment and a vertical signal explaining their evolution. Therefore, and taking into account the results obtained from the analysis of the distribution of these systems, our results indicate that Cro and Yyc systems (and possibly also Eta) were present in the last common ancestor of *Lactobacillaceae *and have been conserved during the evolution of this group.

## Conclusions

The phylogenetic analysis of the OmpR/IIIA systems in *Lactobacillaceae *shows that no new TCS of this family has recently evolved in this group by either lineage-specific gene expansion or domain shuffling. Furthermore, no clear evidence for non-orthologous replacements of either RR or HK partners has been obtained. Therefore, our results strongly suggest that coevolution of cognate RR and HKs has been prevalent in *Lactobacillaceae*. Furthermore, no evidence of recent HGT events has been found for the systems present in more than one species of the group. The detailed analysis of three systems present in most strains used in this study indicates that vertical inheritance has been prevalent in the evolution of these systems. However, a different picture might emerge from the analysis of the other 6 TCS included in this work. Their non-universal distribution in the group of *Lactobacillaceae *species considered can be explained by differential gains and/or losses, which at present cannot be resolved. For this purpose, more complete genome sequences of *Lactobacillaceae *strains and species are necessary.

The picture that emerges from the study of the OmpR/IIIA TCS is that evolution of *Lactobacillaceae *from their last common ancestor and the adaptation process to the habitats that they currently occupy did not require the development of new TCS from systems previously present. Instead, vertical inheritance of TCS present in the last common ancestor and lineage-specific gene losses appear as the main evolutionary forces involved. Although HGT cannot be ruled out, it is worth mentioning that no evidence of recent HGT events have been obtained. This view would agree with the genomic analyses of *Lactobacillales *[29,47] which show that gene losses have been a major trend in the evolution of this group.

## Methods

### Sequences, alignments and phylogenetic information analysis

TCS-encoding genes corresponding to 19 completely sequence genomes of *Lactobacillaceae*/*Leuconostocaceae *(Table 1) were identified by using the tools provided by the Microbial Genome Database for Comparative Analysis (MBGD; http://mbgd.genome.ad.jp/) [43]. Briefly, an orthology table of all genes present in the 19 genomes was obtained using the clustering algorithm implemented in MBGD. The orthology table was queried for response regulators and histidine kinases in order to retrieve the corresponding genes. The genes were confirmed as RRs or HKs by checking the presence of typical conserved domains. Due to the low similarity at the nucleotide level observed in both datasets, amino acid sequences were used for subsequent analyses. In order to obtain additional sequences that might have been bypassed in the first search, similarity searches were performed with BLASTP [48] with the genomic BLAST service provided by the National Center for Biotechnology Information (NCBI; http://www.ncbi.nlm.nih.gov/sutils/genom_table.cgi) against the 19 genomes using a representative sequence of each cluster of orthologs previously identified. In order to obtain putative outgroup sequences for each cluster of orthologs identified, a representative sequence of each cluster was used to query the non-redundant protein sequence database at the NCBI using BLASTP. Sequences not belonging to *Lactobacillaceae *that scored the lowest E-values were selected and checked to belong to the same orthology group than the corresponding query sequence in MBGD. At least two sequences were used as putative outgroup sequences for each cluster of orthologs. Detailed information on the sequences used in these analyses is provided in additional file 1. Multiple alignments were obtained with Muscle [49]. Gaps and positions of doubtful homology were removed using Gblocks [50]. The final multiple alignments used for the analyses are available in additional file 3.

### Phylogenetic reconstruction

In order to obtain accurate phylogenies, the best fit model of amino acid substitution was selected using ProtTest [51]. The AIC, which allows for a comparison of likelihoods from non-nested models, was adopted to select the best models [52]. The phylogenetic signal contained in the different data sets was assessed by likelihood mapping [53] using Tree-Puzzle 5.2 [54]. The models selected by ProtTest were implemented in PhyML [55] to obtain maximum likelihood trees for the different alignments. Bootstrap support values were obtained from 1,000 pseudorandom replicates. Congruence among topologies for TCS genes and/or the reference species tree (see below) was evaluated using Shimodaira-Hasegawa's test [56] implemented in TreePuzzle 5.2 [54] and, when necessary, represented graphically using TreeMap [57].

### Construction of a reference tree

The 141 core proteins identified by Claesson et al. [28] were used to obtain a reference phylogenetic tree for the 19 strains considered in the analysis. The nucleotide sequences were retrieved from MBGD. The sequences were translated into amino acids, aligned with ClustalW and the corresponding nucleotide sequences realigned on the basis of the amino acid alignment using MEGA 4 [58]. Gaps and positions of doubtful homology were removed using Gblocks [50] with default parameters. The resulting multiple alignments were concatenated using the tool available in the Phylemon suite [59]. The best fit model of nucleotide substitution was selected using jModelTest ver. 0.1.1 [60] with the AIC criterion. The phylogenetic reconstruction by maximum likelihood was obtained with PhyML using the previously selected evolutionary model.

## Authors' contributions

MZ conceived of the study, participated in the molecular phylogenetic analyses, participated in design and coordination of the study and drafted the manuscript. CLGE carried out the compilation of sequences and participated in the molecular phylogenetic analyses. FGC participated in the design of the study, supervised the molecular phylogenetic studies and helped to draft the manuscript. All authors read and approved the final manuscript.

## Supplementary Material

Additional file 1 **Supplementary tables**. Supplementary Tables list the genes encoding TCS identified in each of the 19 genomes included in this study. Click here for file

Additional file 2 **Alignments**. A zip file containing the alignments used in this study in either FASTA or Phylip format. Details of the sequences used in this study and the tags used to identify them in the alignment files can be found in the files IIIA-seqs.doc and OmpR-seqs.doc (MS Word). A detailed list of the alignments can be found in the file readme.doc (MS Word). Click here for file

Additional file 3 **Supplementary figures**. Fig. S1: likelihood mapping analysis of OmpR and IIIA sequence alignments. Fig. S2: maximum likelihood phylogenetic trees for OmpR and IIIA sequences. Fig. S3: Pho gene clusters of *Lactobacillaceae*. Fig. S4: likelihood mapping analysis of the sequence alignments of Ycl1 and Ycl2, Pho and 872 RR and Eta and Kin clusters. Fig. S5: likelihood mapping analysis of the sequence alignments of Cro, Eta and Yyc RR and HK encoding genes of *Lactobacillaceae*. Click here for file
