# Supplementary material for: Evolutionary history of the OmpR/IIIA family of signal transduction two component systems in Lactobacillaceae and Leuconostocaceae
Source: BMC Evol Biol. 2011 Feb 1;11:34. doi: 10.1186/1471-2148-11-34 (PMC3040137; doi:10.1186/1471-2148-11-34)
Supplement: Additional file 1 — Supplementary tables. Supplementary Tables list the genes encoding TCS identified in each of the 19 genomes included in this study. [file 1471-2148-11-34-S1.PDF]

Genes encoding two component systems present in the strains analized in this study.

Two component systems in *L. acidophilus* NCFM

Acc. N°	Gene tag	Family	Orthologous cluster	Notes
YP_194633	LBA1798	lytR		
YP_194634	lba1799	HPK 10		
YP_193513	LBA0603	lytR		
YP_193512	lba0602	HPK 10		
YP_194287	LBA1431	OmpR	Bil	
YP_194286	lba1430	IIIA		
YP_194375	LBA1525	OmpR	Eta	
YP_194374	lba1524	IIIA		
YP_193645	LBA0746	OmpR	Ycl1	
YP_193646	lba0747	IIIA		
YP_193011	LBA0078	OmpR	Yyc	
YP_193012	LBA0079	IIIA		
YP_194502	LBA1659	OmpR	Kin	
YP_194503	lba1660	IIIA		
YP_194653	LBA1820	OmpR	Cro	
YP_194652	lba1819	IIIA		

Two component systems in *L. brevis* ATCC 367

Acc. N°	Gene tag	Family	Orthologous cluster	Notes
YP_794400	LVIS_0203	citB		
YP_794399	lvis_0202	IV		
YP_794363	lvis_0163	LytR		
YP_794364	lvis_0164	HpK10		
YP_794365	lvis_0165	HpK10		
YP_794807	Lvis_0631	OmpR	Pho	
YP_794808	lvis_0632	IIIA		
YP_795450	lsei_1316	OmpR	Ycl1	
YP_795449	lvis_1315	IIIA		
YP_796144	lvis_2057	OmpR	Cro	
YP_796143	lvis_2056	IIIA		
YP_794236	lvis_0029	OmpR	Yyc	
YP_794237	lvis_0028	IIIA		
YP_794550	lvis_0354	OmpR	Cia	
YP_794551	lvis_0355	IIIA		
YP_795182	lvis_1018	OmpR	Eta	
YP_795181	lvis_1017	IIIA		
YP_79451	lvis_0317	OmpR	Kin	
YP_794513	lvis_0316	IIIA		
YP_796118	lvis_2030	YcbB		
YP_796117	lvis_2029	IV		

Two component systems in *L. casei*

Strain BL23 Acc. N°	Gene tag	ATCC 334 Acc. N°	Gene tag	Family	Orthologous cluster	Notes
YP_001988979	LCABL_30710	YP_808020	LSEI_2868	IV		
YP_001988980	LCABL_30720	YP_808021	LSEI_2869	CitB		
YP_001988485	LCABL_25610					Pseudogene
YP_001988486	LCABL_25620	YP_807570	lsei_2389	lytT		
YP_001988675	LCABL_27660	YP_807763	Lsei_2600	HpK10		No homologs in LAB
YP_001988674	LCABL_27650	YP_807762	Lsei_2599	lytT		
YP_001987383	LCABL_14440	YP_806451	Lsei_1223	II		
YP_001987382	LCABL_14430	YP_806450	Lsei_1222	NarL		
YP_001987821	LCABL_18840	YP_806881	Lsei_1666	II		
YP_001987820	LCABL_18830	YP_806880	lsei_1665	NarL		
YP_001986199	LCABL_02090	YP_805525	LSEI_0220	IIIA	Cro	
YP_001986198	LCABL_02080	YP_805524	LSEI_0219	OmpR		
YP_001986511	LCABL_05270	YP_805756	lsei_0461	IIIA	460	No homologs in LAB
YP_001986510	LCABL_05260	YP_805755	lsei_0460	OmpR		
YP_001986760	LCABL_07770	YP_805994	LSEI_0712	IIIA	Cia	
YP_001986759	LCABL_07760	YP_805993	LSEI_0711	OmpR		
YP_001986993	LCABL_10490	YP_806176	Lsei_0935	IIIA	Pho	
YP_001986992	LCABL_10480	YP_806175	Lsei_0934	OmpR		
YP_001987009	LCABL_10650	YP_806192	LSEI_0951	IIIA	950	No homologs in LAB
YP_001987008	LCABL_10640	YP_806191	LSEI_0950	OmpR		
YP_001987149	LCABL_12060	YP_806278	Lsei_1042	IIIA	Ycl1	
YP_001987148	LCABL_12050	YP_806277	Lsei_1041	ompR		
YP_001987366	LCABL_14270	YP_806436	LSEI_1208	IIIA	1209	No homologs in LAB
YP_001987367	LCABL_14280	YP_806437	LSEI_1209	OmpR		
YP_001987581	LCABL_16420	YP_806639	LSEI_1419	IIIA	Bce	
YP_001987582	LCABL_16430	YP_806640	LSEI_1420	OmpR		
YP_001987834	LCABL_18970	YP_806893	LSEI_1678	IIIA	Eta	
YP_001987835	LCABL_18980	YP_806894	LSEI_1679	OmpR		
YP_001987897	LCABL_19610	YP_806955	LSEI_1741	IIIA	Bce	
YP_001987896	LCABL_19600	YP_806954	LSEI_1740	OmpR		
YP_001988779	LCABL_28710	YP_807841	lsei2680	IIIA	Kin	
YP_001988780	LCABL_28720	YP_807842	LSEI_2681	OmpR		
YP_001988920	LCABL_30120	YP_807963	LSEI_2807	IIIA	Yyc	
YP_001988921	LCABL_30130	YP_807964	LSEI_2808	OmpR		

Two component systems in *Lactobacillus delbrueckii* subsp. *bulgaricus*

Strain ATCC 11842		ATCC BAA-365				
Acc. N°	Gene tag	Acc. N°	Gene tag	Family	Orthologous cluster	Notes
YP_618256	Ldb0026	YP_812153	LBUL_0021	lytR		
		YP_812154	LBUL_0022	HPK 10		Pseudogene (11842)
YP_618325	Ldb0135	YP_812230	LBUL_0111	OmpR	Yyc	
YP_618326	Ldb0136	YP_812231	LBUL_0112	IIIA		
YP_618755	Ldb0687	YP_812688	LBUL_0621	OmpR	Ycl1	
YP_618756	Ldb0688	YP_812689	LBUL_0622	IIIA		
YP_618911	ldb0877	YP_812846	LBUL_0802	OmpR	Bil	
YP_618912	Ldb0878	YP_812847	LBUL_0803	IIIA		
		YP_812892	LBUL_0872	OmpR	872	No homologs in LAB;
YP_618959	Ldb0963	YP_812893	LBUL_0873	IIIA		pseudogene (11842)
YP_619319	Ldb1493	YP_813340	LBUL_1389	OmpR	Eta	
YP_619318	Ldb1492	YP_813339	LBUL_1388	IIIA		
YP_619677	Ldb2046	YP_813730	LBUL_1893	OmpR	Cro	
YP_619676	Ldb2045	YP_813729	LBUL_1892	IIIA		

Two components systems present in *L. fermentum* IFO 3956

Acc. N°	Gene tag	Family	Orthologous cluster	Notes
YP_001842831	LAF_0015	OmpR	Yyc	
YP_001842832	LAF_0016	IIIA		
YP_001842923	LAF_0107	OmpR	Cro	
YP_001842924	LAF_0108	IIIA		
YP_001843213	LAF_0397	OmpR	Ycl1	
YP_001843214	LAF_0398	IIIA		
YP_001844046	LAF_1230	OmpR	Pho	Orphan
YP_001844114	LAF_1298	OmpR	Eta	
YP_001844113	LAF_1297	IIIA		
YP_001843887	LAF_1071	NarL		
YP_001843886	LAF_1070	II		
YP_001843113	LAF_0297	YcbB		Orphan

Two component systems in *Lactobacillus gasseri* ATCC 33323

Acc. N°	Gene tag	Family	Orthologous cluster	Notes
YP_813914	LGAS_0064	OmpR	Yyc	
YP_813915	LGAS_0065	IIIA		
YP_815198	LGAS_1398	OmpR	Eta	
YP_815197	LGAS_1397	IIIA		
YP_815065	LGAS_1261	OmpR	Ycl1	
YP_815064	LGAS_1260	IIIA		
YP_815511	LGAS_1735	OmpR	Cro	
YP_815509	LGAS_1734	IIIA		
YP_814540	LGAS_0710	OmpR	Bil	
YP_814541	LGAS_0711	IIIA		

Two component systems in *Lactobacillus helveticus* DPC 4571

Acc. N°	Gene tag	Family	Orthologous cluster	Notes
YP_001577210	lhv_0790	OmpR	Ycl1	
YP_001577211	lhv_0791	IIIA		
YP_001576655	lhv_0095	OmpR	Yyc	
YP_001576656	lhv_0096	IIIA		
YP_001578007	lhv_1855	OmpR	Cro	
YP_001578006	lhv_1854	IIIA		
YP_001577811	lhv_1588	OmpR	Eta	
YP_001577810	lhv_1587	IIIA		

Two component systems in *L. johnsonii* NCC533

Acc. N°	Gene tag	Family	Orthologous cluster	Notes
NP_964619	LJ0766	lytR		
NP_964617	LJ0764	HPK 10		
NP_964474	LJ0449	lytR		
NP_964473	LJ0448	HPK 10		
NP_965466	LJ1659	NarL		
NP_965465	LJ1658	II		
NP_964773	LJ0918	OmprR	Ycl1	
NP_964774	LJ0919			
NP_964081	LJ0065	OmpR	Yyc	
NP_964082	lj0066			
NP_965391	LJ1587	OmpR	Bil	
NP_965390	LJ1586			
NP_965692	LJ0563	OmpR	Cro	
NP_965691	LJ0564			
NP_965437	LJ1631	OmpR	Eta	
NP_965436	Lj1630			
NP_964988	LJ1132	OmpR	Kin	
NP_964989	lj1133			

Two component systems in *L. plantarum* WCFS1

Acc. N°	Gene tag	Family	Orthologous group	Notes
NP_786831	lp_3638	AraC		No homologs in LAB
NP_786832	lp_3639	I		
NP_784991	lp_1356	LytR		
NP_784990	lp_1355	HPK 10		
NP_786780	Lp_3580	lytR		
NP_786781	Lp_3581	HPK10		
NP_786393	lp_3087	lytR		
NP_786394	Lp_3088	HPK 10		
NP_784214	lp_0418	lytR		
NP_784213	lp_0417	lytR		
NP_784212	lp_0416	HPK 10		
NP_786066	lp_2665	lytR		Orphan
NP_785480	Lp_1942	NarL		
NP_785481	lp_1943	II		
NP_785095	lp_1487	NarL		
	lp_1488	II		
NP_784099	lp_0283	OmpR	Kin	
NP_784098	lp_0282	IIIA		
NP_784495	lp_0743	OmpR	Pho	
NP_784496	Lp_0744	IIIA		
NP_785146	lp_1544	OmpR	Eta	
NP_785147	Lp_1545	IIIA		
NP_786469	Lp_3191	OmpR	Cro	
NP_786468	lp_3190	IIIA		
NP_783896	Lp_0036	OmpR	Yyc	
NP_783897	Lp_0037	IIIA		
NP_785945	lp_2506	OmpR	Ycl1	
NP_785944	lp_2505	IIIA		

Two component systems in *Lactobacillus reuteri*.

Strain JCM 1112 Acc. N°	Gene tag	DSM 20016 Acc. N°	Gene tag	Family	Orthologous cluster	Notes
YP_001841846	Lar_0850	YP_001271504	Lreu_0904	lytR		
YP_001841845	Lar_0849	YP_001271503	Lreu_0903	I		
YP_001841840	Lar_0844	YP_001271498	Lreu_0898	NarL		Orphan
YP_001841943	Lar_0947			NarL		
YP_001841942	Lar_0946			II		
YP_001841014	Lar_0018	YP_001270632	Lreu_0019	OmpR	Yyc	
YP_001841015	Lar_0019	YP_001270633	Lreu_0020	IIIA		
YP_001841051	Lar_0055	YP_001270670	Lreu_0058	OmpR	Bil	
YP_001841052	Lar_0056	YP_001270671	Lreu_0059	IIIA		
YP_001841107	Lar_0111	YP_001270726	Lreu_0117	OmpR	Cro	
YP_001841108	Lar_0112	YP_001270727	LREU_0118	IIIA		
YP_001841407	Lar_0411	YP_001271022	Lreu_0416	OmpR	Ycl1	
YP_001841408	Lar_0412	YP_001271023	LREU_0417	IIIA		
YP_001842157	Lar_1161	YP_001271822	Lreu_1228	OmpR	Eta	
YP_001842156	Lar_1160	YP_001271821	lreu_1227	IIIA		
YP_001842471	Lar_1475	YP_001272149	Lreu_1569	OmpR	Pho	Orphan
YP_001842668	Lar_1672	YP_001272363	Lreu_1787	OmpR	Bce	
YP_001842669	Lar_1673	YP_001272364	LREU_1788	IIIA		
YP_001841414	Lar_0418	YP_001271030	Lreu_0424	YcbB		Orphan

Two component systems in *Lactobacillus sakei* 23K.

Acc. N°	Gene tag	Family	Orthologous cluster	Notes
YP_395175	lsa0563	lytR		
YP_395174	lsa0562			Pseudogene
YP_396109	lsa1500	lytR		
YP_396110	lsa1501	HPK 10		
YP_395161	lsa0549	NarL		
YP_395160	Lsa0548			Pseudogene
YP_395980	lsa1369	NarL		
YP_395981	lsa1370	II		
YP_394688	LSA0077	OmpR	Yyc	
YP_394689	lsa0078	IIIA		
YP_394891	lsa0277	OmpR	Cro	
YP_394892	lsa0278	IIIA		
YP_395112	lsa0500	OmpR	Pho	
YP_395113	lsa0501	IIIA		
YP_395827	lsa1215	OmpR	Ycl1	
YP_395826	lsa1214	IIIA		
YP_395994	lsa1384	OmpR	Eta	
YP_395993	lsa1383	IIIA		
YP_396063	lsa1454	OmpR	Bce	
YP_396064	lsa1455	IIIA		

Two component systems in *Lactobacillus salivarius* UCC118

Acc. N°	Gene tag	Family	Orthologous cluster	Notes
YP_536799	LSL_1912	lytR		
YP_536800	LSL_1913	HPK 10		
YP_534987	LSL_0083	NarL		
YP_534988	LSL_0084	II		
YP_536690	LSL_1803	NarL		
YP_536691	LSL_1804	II		
YP_536073	LSL_1182	OmpR	Pho	
YP_536072	LSL_1181			
YP_536052	LSL_1161	OmpR	Ycl1	
YP_536051	lsl_1160			
YP_534941	LSL_0035	OmpR	Yyc	
YP_534942	lsl_0036			
YP_535103	LSL_0205	OmpR		
YP_535104	lsl_0206		Cro	
YP_535414	LSL_0522	OmpR	Eta	
	LSL_0523			

Two component systems in *Leuconostoc citreum* KM20

Acc. N°	Gene tag	Family	Orthologous cluster	Notes
YP_001727311	LCK_00034	LytR		
YP_001727310	LCK_00033			Pseudogene
YP_001727462	LCK_00185	OmpR	Yyc	
YP_001727463	LCK_00186	IIIA		
YP_001728532	LCK_01263	OmpR	Pho	
YP_001728530	LCK_01261	IIIA		
YP_001727678	LCK_00401	OmpR	Eta	
YP_001727679	LCK_00402	IIIA		
YP_001727532	LCK_00255	OmpR	Cro	
YP_001727533	LCK_00256	IIIA		
YP_001728754	LCK_01486	OmpR	Cia	
YP_001728753	LCK_01485	IIIA		
YP_001727873	LCK_00596	OmpR	Bce	
YP_001727872	LCK_00595	IIIA		

Two component systems in *Leuconostoc mesenteroides* subsp. *mesenteroides* ATCC 8293

Acc. N°	Gene tag	Family	Orthologous cluster	Notes
YP_817618	LEUM_0093	lytR		
YP_817619	LEUM_0094	HPK 10		
YP_817553	LEUM_0008	lytR		
YP_817554	LEUM_0009			
YP_818613	LEUM_1141	NarL		No homologs in LAB
YP_818614	LEUM_1142	II		
YP_819394	LEUM_1950	OmpR	Yyc	
YP_819393	LEUM_1949	IIIA		
YP_818072	LEUM_0585	OmpR	Pho	
YP_818075	LEUM_0588	IIIA		Pos. pseudogene; inserted C between 558497-98 in CP000414
YP_818315	LEUM_0834	OmpR	Bce	
	LEUM_0835	IIIA		
YP_819270	LEUM_1810	OmpR	Cia	
YP_819269	LEUM_1809	IIIA		
YP_817847	LEUM_0351	OmpR	Cro	
YP_817848	LEUM_0352	IIIA		
YP_819192	LEUM_1732	OmpR	Eta	
YP_819191	LEUM_1731	IIIA		
YP_818435	LEUM_0956	OmpR	Ycl2	
YP_818436	LEUM_0957	IIIA		

Two component systems in *Oenococcus oeni* PSU-1

Acc. N°	Gene tag	Family	Orthologous cluster	Notes
YP_810468	OEOE_0885	lytR		
YP_810467	OEOE_0884	I		
YP_809765	OEOE_0105	OmpR	Yyc	
YP_809766	OEOE_0106	IIIA		
YP_809779	OEOE_0119	OmpR	Cro	
YP_809780	OEOE_0120	IIIA		
YP_809801	OEOE_0142	OmpR	Cia	
YP_809802	OEOE_0143	IIIA		
YP_810107	OEOE_0488	OmpR	Kin	
YP_810108	OEOE_0489	IIIA		
YP_811278	OEOE_1773	OmpR	Ycl2	
YP_811277	OEOE_1772	IIIA		

Two component systems in *Pediococus pentosaceus* ATCC 25745

Acc. N°	Gene tag	Family	Orthologous group	Notes
YP_803637	PEPE_0084	lytR		
YP_803636	PEPE_0083	HPK 10		
YP_804199	PEPE_0696	lytR		
YP_804198	PEPE_0695	I		
YP_805196	PEPE_1733	NarL		
YP_805197	PEPE_1734	II		
YP_803969	PEPE_0431	OmpR	Pho	
YP_803970	PEPE_0432	IIIA		
YP_804217	PEPE_0714	OmpR	Eta	
	PEPE_0715	IIIA		
YP_804835	PEPE_1354	OmpR	Ycl1	
YP_804834	PEPE_1353	IIIA		
YP_805180	PEPE_1717	OmpR	Cro	
YP_805179	PEPE_1716	IIIA		
YP_805260	PEPE_1797	OmpR	Yyc	
YP_805259	PEPE_1796	IIIA		
YP_805095	PEPE_1632	YcbB		Orphan
