# Supplementary material for: Evolutionary history of the OmpR/IIIA family of signal transduction two component systems in Lactobacillaceae and Leuconostocaceae
Source: BMC Evol Biol. 2011 Feb 1;11:34. doi: 10.1186/1471-2148-11-34 (PMC3040137; doi:10.1186/1471-2148-11-34)

Figure S1. Likelihood mapping analysis of the sequence alignments of OmpR and IIIA encoding genes present in *Lactobacillaceae*. The regions at the corners of the triangles correspond to the three possible tree topologies for a quartet; the lateral regions to partly resolved trees and the central region to unresolved trees. The numbers indicate the percentage of quartets falling in each region.

Figure S2. Maximum likelihood phylogenetic tree for OmpR RR (left) and IIIA HK (right) sequences used in this study. The species and the locus tags of the corresponding genes are indicated. The main groups derived from the analyses are indicated. The trees are arbitrarily rooted with cluster Bil. Support values for the bootstrap analysis are given for those nodes with at least 50% of bootstrap support.

Figure S3. Schematic representation of the genetic organization of gene clusters present in *Lactobacillaceae* encoding for the phosphate uptake system and the cognate TCS. Colors indicate homologous genes or domains.

Figure S4. Likelihood mapping analysis of the sequence alignments of Ycl1 and Ycl2 HK encoding genes present in *Lactobacillaceae* (A), Pho and 872 HK (B), Pho and 872 RR (C), Eta and Kin HK (D) and Eta and Kin RR (E). See Fig. S1 for interpretation of the diagrams.

Figure S5. Likelihood mapping analysis of the sequence alignments of Cro HK, RR and the concatenated alignment (A), Eta HK and RR (B) and Yyc HK and RR (C). See Fig. S1 for interpretation of the diagrams.

Supplementary Fig. S1

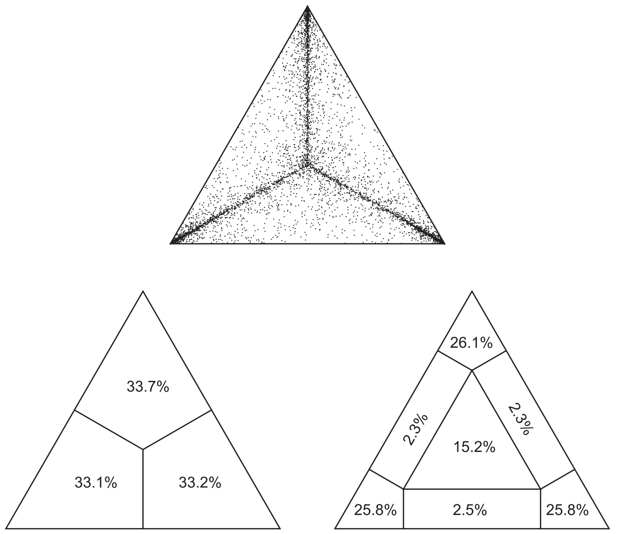

OmpR RR

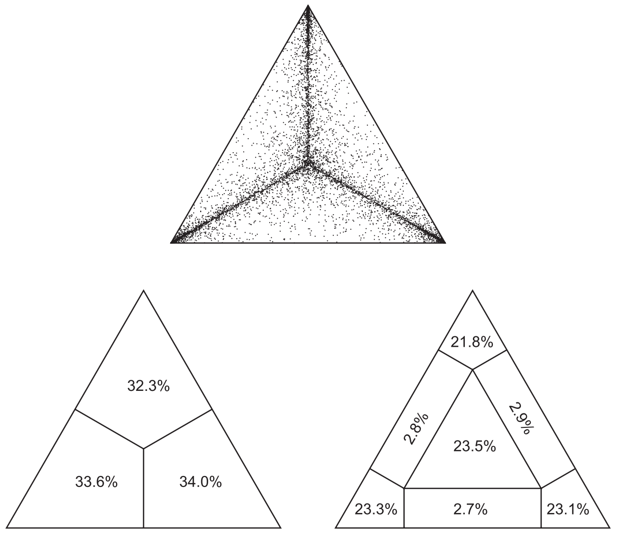

IIA HK

RR HK

Supplementary Fig. S2

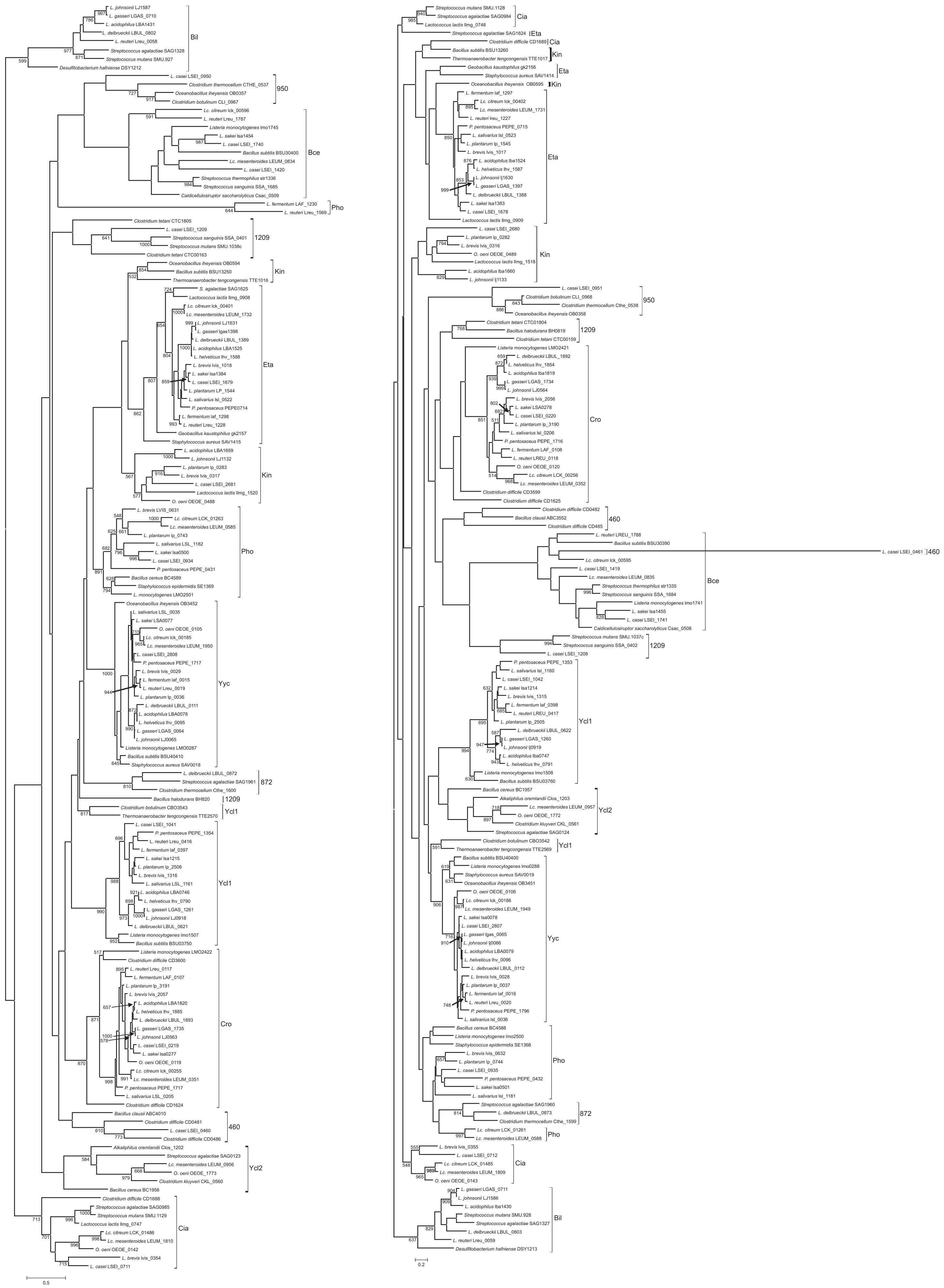

Supplementary Fig. S3

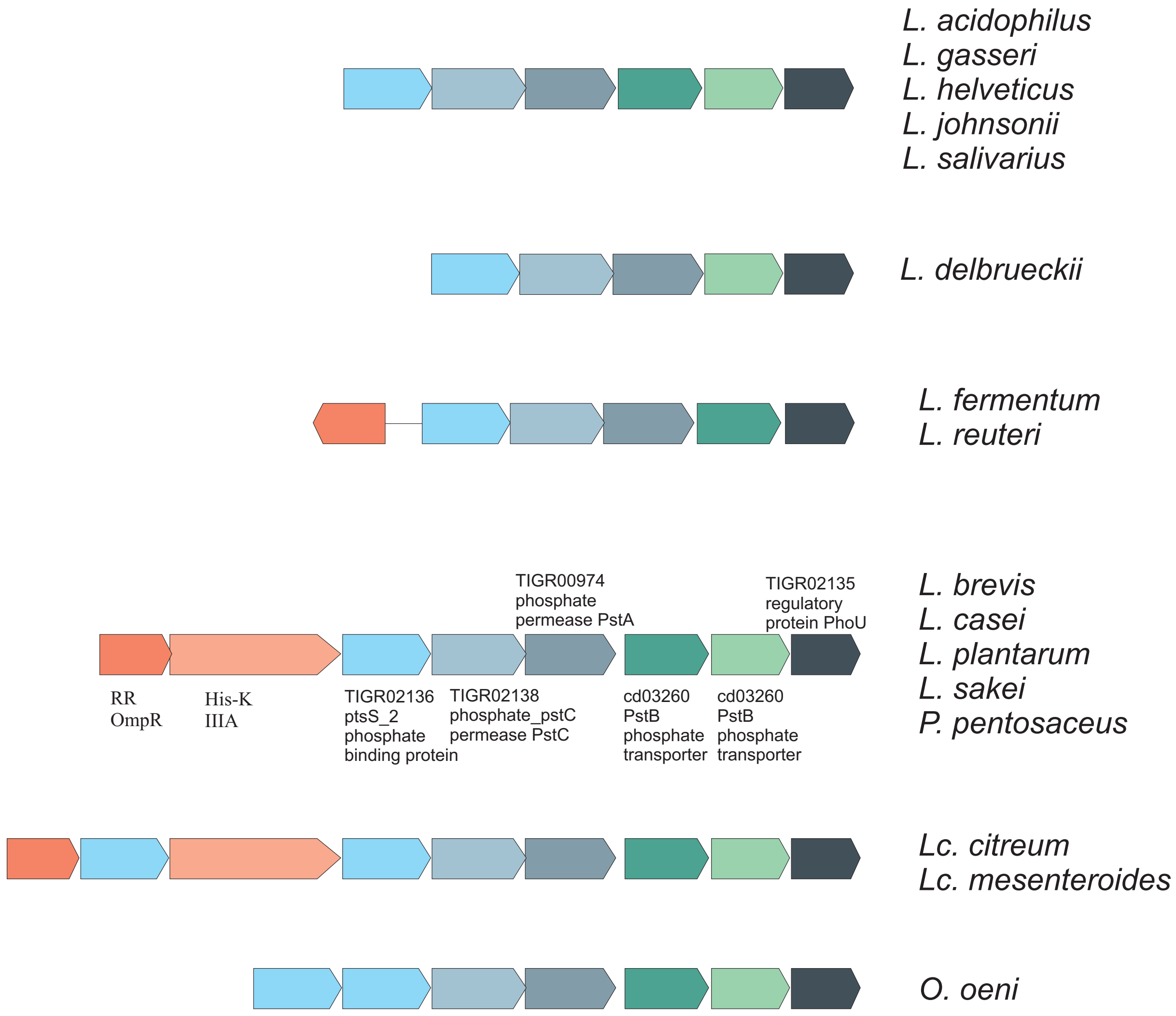

Supplementary Fig. S4

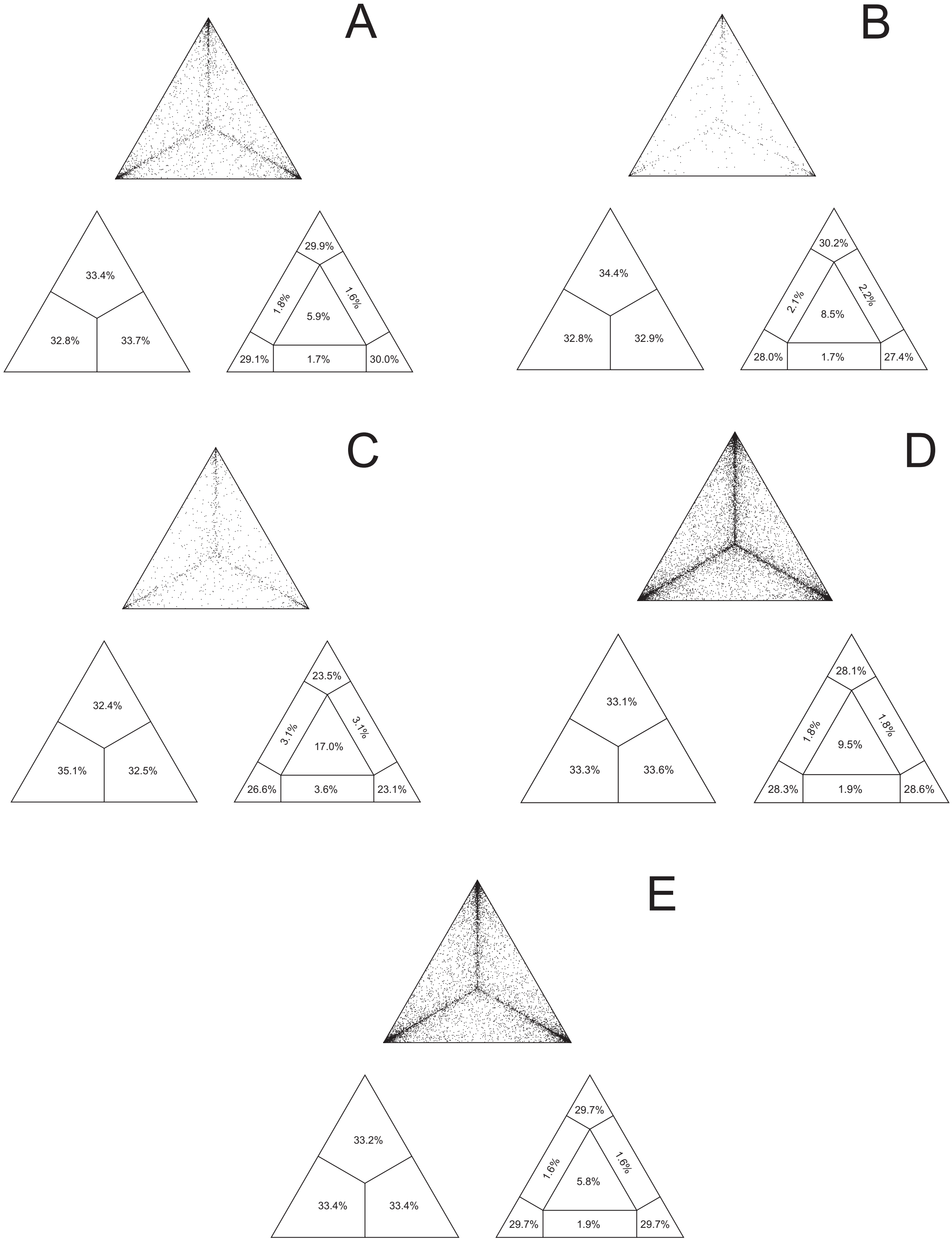

Supplementary Fig. S5

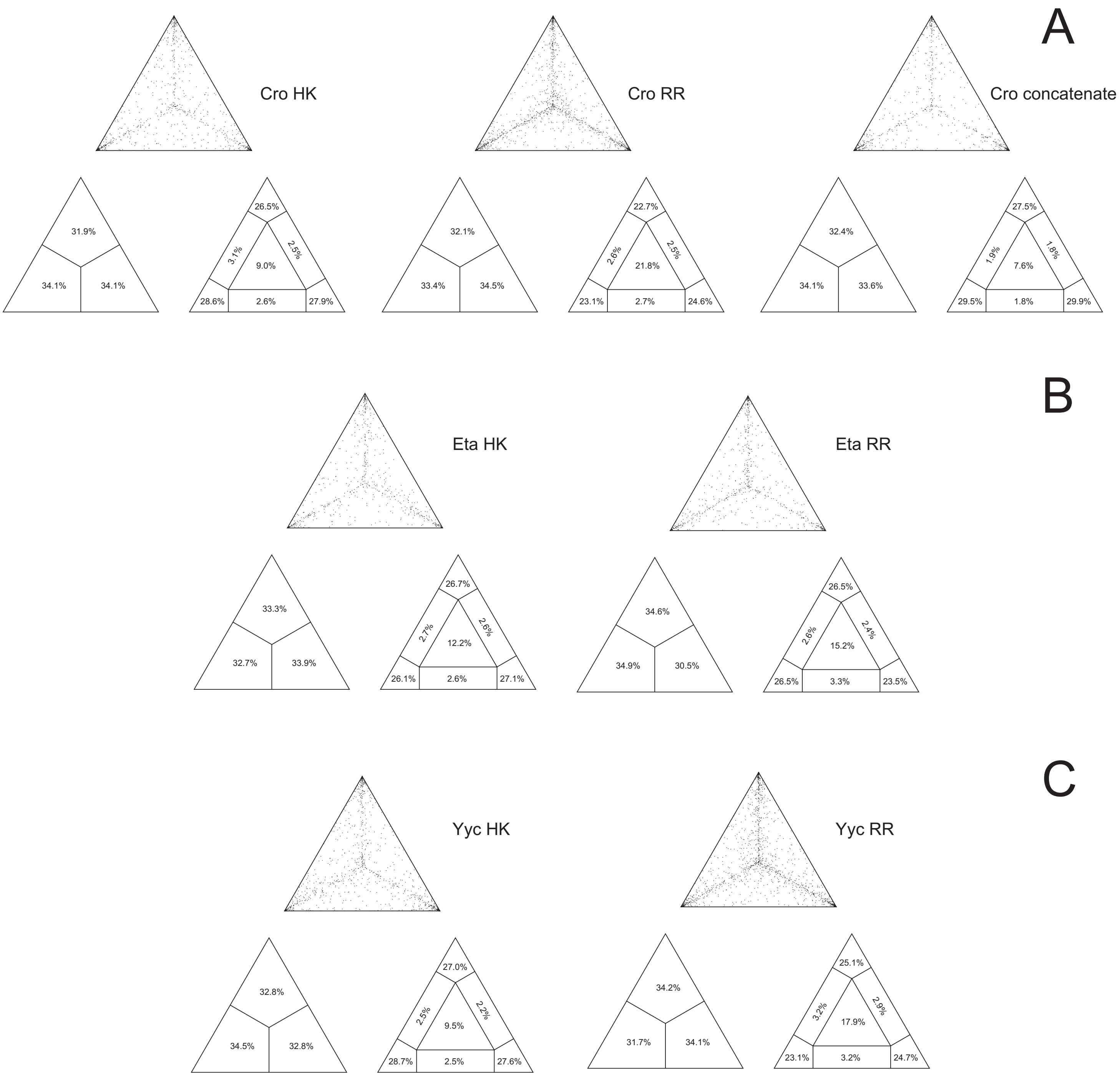

Supplement: Additional file 3 — Supplementary figures. Fig. S1: likelihood mapping analysis of OmpR and IIIA sequence alignments. Fig. S2: maximum likelihood phylogenetic trees for OmpR and IIIA sequences. Fig. S3: Pho gene clusters of Lactobacillaceae. Fig. S4: likelihood mapping analysis of the sequence alignments of Ycl1 and Ycl2, Pho and 872 RR and Eta and Kin clusters. Fig. S5: likelihood mapping analysis of the sequence alignments of Cro, Eta and Yyc RR and HK encoding genes of Lactobacillaceae. [file 1471-2148-11-34-S3.PDF]
