# Supplementary material for: Evolutionary history of the OmpR/IIIA family of signal transduction two component systems in Lactobacillaceae and Leuconostocaceae
Source: BMC Evol Biol. 2011 Feb 1;11:34. doi: 10.1186/1471-2148-11-34 (PMC3040137; doi:10.1186/1471-2148-11-34)
Supplement: Additional file 2 — Alignments. A zip file containing the alignments used in this study in either FASTA or Phylip format. Details of the sequences used in this study and the tags used to identify them in the alignment files can be found in the files IIIA-seqs.doc and OmpR-seqs.doc (MS Word). A detailed list of the alignments can be found in the file readme.doc (MS Word). [file 1471-2148-11-34-S2.ZIP › alignments/IIIA-seqs.doc]

>gi|116493791|ref|YP_805525.1| Signal transduction histidine kinase [Lactobacillus casei ATCC 334] lsei_0220 lsei220

MSEKVKLTGKEKSELWIEGIVTVILLLMLNFALLVLINQMIAHNPGLENAIWGVKTNLTFGSRGFHLWSW

SNLFLALMAIADVIVVYWRLARRYRQMQMRHVIAELHFIADGHLDHRIKFEVNTELQKVVSSINALVDST

VNSMAEERRIEQSKDELITNVSHDIRTPLTSIIGYLGLIEDHQYRSEEELLKYTHTAYLKAKQMKVLVED

LFEYTKVKSTTTKLNPVRFNMIAMLEQLAASFELEAHKKHMRILVSGEPDPLMMEADTEKLGRVFNNLIV

NALKYGQGGRHIFLDAQRIGSEVVVKVSNDGAQIPNDALSQLFDRFYRVEESRSQETGGTGLGLAIAQSI

IALHGGYIYADSSPEITSFVIHLPLKVGASLTDNKNKSNEEAGKSK

>gi|116493444|ref|YP_805179.1| Signal transduction histidine kinase [Pediococcus pentosaceus ATCC 25745] PEPE_1716 pepe220

MKLTGREKYELFGEGVITVILLLLLNLSVFILIDSALKSNPALSNAVFMVKNSITIGGVQIWSYQKIFIL

IMFLADAGVLYWRLERRYKQMQLRHIIAELHYIANGHFDHRIPFELSGDHQRVVDSVNSLVDSVISSMEE

ERALKQSKDDLITNVSHDLRTPLTSIIGYLRLIEDRQFKNEDDILKYTHTAYLKSIQMKSLVEDLFEYTK

VSQSNPHLTINTINVDSMLQQLAASFELEAKQKHFVITSQCTPKDLEITGDAEKIGRVFNNLITNALKYG

NGGKNIFLSAKQINDTVIFEVANDGEKVPAEALGKLFDRFYRVEGSRSKATGGTGLGLAIAQSIVEMHNG

TIEAHSNDKRTSFVIQLPLHQPVSKDLP

>gi|116490236|ref|YP_809780.1| Signal transduction histidine kinase [Oenococcus oeni PSU-1] OEOE_0120 oeoe220

MKLTAKEILHLSFYGAAAAIVLLFLDWSVFSFFTAILSGNFRISRGGFYSLARITFNSHNFTFKNQQWWG

FVAILLIGIVLWIFLLLREYQQLQLKRLSNEITRINNTNDNDQIKMTSIDPMVTNLVSQINQMTQAKQEI

LDTELDNEKKQQELVTNVSHDLRTPLTSIIGYLGLIESNPKLPEEQMRKHVSTAYSKAQQLVSMVEDLFS

YSQTQTNGENMNFQPMEISEFFDQLIVQFSLDAHKHNITMSSLTNPDQIKIVADPEKLARLFMNLINNAF

KYGKSAMTFIKLTAVKKDDRVEIRVQNDGEQIPKSSLNKLFDRFYRVDQSGNKDISGTGLGLAISQGIVK

QHHGAIRVESNTQLTSFIIELPLDASLGMPRD

>gi|161508039|ref|YP_001578006.1| Two-component sensor [Lactobacillus helveticus DPC 4571] lhv_1854 lhel220

MKKERVKLTGAEKSELFAEGIVTIVLLLLLNLSIIILIHLAILQDESLVNGIYFLKKTMTFAGGYHLWSW

QNTFIILMGVGDIVVLYWRLIRRYHQMQLRHVILELHYIASGHFDHRINFRVRPDLQRVVDSINSLVDST

VHSINEERAIEQSKDELITNVSHDIRTPLTSIIGYLGLLKSGVASPEDQQKYVDIAYTKAEQMKSLANDL

FEYTTLKSTKLNLTSLHIYSMLEQVAAGFELEAEKKGIEIKIEARPKDLTIRADAEKLVRVYNNLISNAL

KYGIGATQIKLVANLVNKQEVELRVENNGEPIPKKSLKKIFDRFYRVETSRNTKTGGTGLGLSITEGVVD

LHHGTIRCESDKNWTSFIIRLPLNPKKTNSKG

>gi|116617477|ref|YP_817848.1| Signal transduction histidine kinase [Leuconostoc mesenteroides subsp. mesenteroides ATCC 8293] LEUM_0352 leum220

MTKVALILKHNRTKIYYLVVTILLLLLFSWTIMTGFDALRHRFSFISFNIWNVLEFVTFVSGNIGIVFWQ

YLKWRRDESLYRLIREMRQVARENSDMTISWRAPYAPTTQALIDVSNTIAENTRRIREEERQSERSKDEM

ITNISHDLRTPLTAIIGYLGLVEMGQALSEKDRKKYIHTAYSKSNQMKVLVEDLFEYSQTQAQDAALNIT

SLSLNDLFGQLLAGYELEAQEKNIALNQLTNPEMIVIEADSDKLARVLMNLVSNAFKYGDGATFIKLTAQ

IDDKNVVIRVTNDGVKIPDEAIGDIFDRFYRVESSRNSKTGGTGLGLAIVKGIVVQHHGSVAVESDDDMT

SFIITLPLKQT

>gi|148543357|ref|YP_001270727.1| integral membrane sensor signal transduction histidine kinase [Lactobacillus reuteri F275] LREU_0118 lreut220

MKLTGREKSSLILEGVVTVILLLLLNMAIIVIIQDAIQSNPGVTNGIFMIKQSLKIGPLQAQIWSYQRIL

IAFLVIIDVWVVWWRLRRRYHLYQMDHIIAELHYIAQGHLDHRIPFRLKGNQQHVITSVNALVDSAVRSM

DDERKIEKSKDELITNVSHDLRTPLTSIIGYLGLIEDKQYRSEEDILKYTHTAYEKAKQMKTLVDDLFEY

TKVQQHGAPVNIMKIDLNQLIEQLTASFELEAQHRGIEITSSVIPNPLMIEADPEKLGRVFNNLVSNAFK

YGNGASYIRINARQENDMVVVKVANDGTPIPEKALDHLFERFYRAEASRSRATGGTGLGLAIVKSIVDLH

HGSVKVTSTEDETAFIVTLPLKQEVKENKQK

>gi|90961188|ref|YP_535104.1| Two component system histidine kinase [Lactobacillus salivarius subsp. salivarius UCC118] lsl_0206 lsal220

MKFHITGREKSELLGEGILTIILLILLNMSLYVILNQLLVSNPDLASGIFLIKTSLDFKYAHIQLWSWGW

IFIILMAVVDVIVVYWRLVRRYHQMQLRHIINELHYIADGHFNHRIPFQLKGQMQRVVASVNALVDSTIK

AMEDEREIERSKDELITNVSHDIRTPLTSIIGYLGLIEDKQYRSEEDILKYTHTAYLKSKQMKSLVDDLF

EYTKVRQTDAPLQIQSLELNAMLEQIGASFELEANKKGMEINTILPDSPVKIEADPEKLARVFNNLITNA

LKYGSDGKNIYLQLTKVDEKEIEIRISNDGEPIPEKSLRQVFDRFYRVESSRSKETGGTGLGLAIAQSII

ELHHGYIYVESNKNLTSFIMRLPIKHDNVVKKS

>gi|42519761|ref|NP_965691.1| two-component system histidine kinase [Lactobacillus johnsonii NCC 533] LJ0564 ljoh220

MKKQKVILTTKEKSELFGEGVITVILLLLLNLSIIILINLAVLNDPRLENGIFFLKKTITFANGMHLWSW

QRLFISFMLVGDAIVVYWRLIRRYRQMQLRHVIDELHYIADGHFDHRIPFVVKTDLQKVIDSINALVDST

VASMEEERQIEQSKDDLITNVSHDIRTPLTSIIGYLGLLKSSELNEDQTKYIQIAYDKALQMKALAEDLF

EYTTLRSSTNNKLVLTPLHVNSMLEQVAAGFELEAEKKNITFNVITRPRDLVIDADAKMIVRMLNNLISN

ALKYGHGATEINLIANKVNNEFVELRVENNGEQIPKKSLQKIFDRFYRVESSRNLKTGGTGLGLAITKSI

VDLHGGNIKCQSTAELTSFIIQLPLHSPKKKDGVL

>gi|116630288|ref|YP_815509.1| Signal transduction histidine kinase [Lactobacillus gasseri ATCC 33323] LGAS_1734 lgas220

MKKQKVILTTKEKSELFGEGVITVILLLLLNLSIIILLNLAVLNDPRLENGIFFLKKTITFANGMHLWSW

QRLFVGVMLVGDAIVVYWRLIRRYRQMQLRHVIEELHYIADGHFDHRIPFVVKTDLQKVIDSINALVDST

VASMEEERQIEQSKDDLITNVSHDIRTPLTSIIGYLGLLKSSELNEDQAKYIKIAYDKALQMKALAEDLF

EYTTLRSSTNNKLVLAPLHVNSMLEQVAAGFELEAEKKNIAFNVVTRPRDLVIDADAKMIVRMLNNLISN

ALKYGHGATEINLIANKVNNKFVELRVENNGEQIPKKSLQKIFDRFYRVESSRNLKTGGTGLGLAITKSI

VDLHGGTIKCQSTSELTSFIIQLPLNSPKAK

>gi|104774696|ref|YP_619676.1| Two-component system, sensor histidine kinase [Lactobacillus delbrueckii subsp. bulgaricus ATCC 11842] ldb2045 Lactobacillus delbrueckii subsp. bulgaricus ATCC BAA-365 YP_813729 LBUL_1892 lbul220

MKLEQDKEKQVVQGKRPRVKLTAAEKSELFAEGVVTVIIMLLLNMSVIILYHLAVLQDKSLVNGIYFLKK

TMTIGPGYHIWSWERIGIILMGIADVIVLYWRLIRRYHQMQLRHIISELHYIANGHFDHRISFSVNNDMQ

KVIDSINSLVDSTVGAINEEKAIEQSKDELITNVSHDIRTPLTSIIGYLGLLKNGAVTSQEDMLKYINIA

YDKAEQMKSLANDLFEYTTLKSTKTKLNVTPINIKGMMEQVAAGFELEAEKKGIAFSVKARPDDLIVNAD

VEKLVRVYNNLISNALKYAAGASRINLVANLINHEQVELRVENNGEPIPKDKLKKIFDRFYRVESSRNTK

TGGTGLGLSIVQGGVELHGGTIRCESNKDWTSFIILLPRDPQANNFALRPVV

>gi|58338067|ref|YP_194652.1| two-component sensor histidine kinase homolog [Lactobacillus acidophilus NCFM] lba1819 laci220

MKKERVKLTGAEKSELFAEGIVTIILLLLLNLSIIILIHLAILQDESLVNGIYFLKKSMTFVGGRHVWSW

QNIFIIIMAVGDLAVLYWRLIRRYHQMQLRHVISELHYIANGHFDHRINFRVRPELQRVVDSINSLVDST

VNSINEERAIEKSKDELITNVSHDIRTPLTSIIGYLGLLKTGISSKEDQQKYVDIAYTKAEQMKSLADDL

FEYTTLKSTSTKLNLNELHIYSMLEQVAAGFELEAEKKGIDIEIEARPKNLTIQADAEKLVRVYNNLISN

AFKYGTGATKIKLVANLVNKSEVELRVENNGEPIPVSAQKKIFDRFYRVETSRNTKTGGTGLGLSITKSV

VDLHHGTIRCQSDENWTSFIIRLPLDPNKAK

>gi|28379576|ref|NP_786468.1| histidine protein kinase; sensor protein [Lactobacillus plantarum WCFS1] lp_3190 l lpla220

MKLTGREKSELFFEGVVTVILLLLLNLSIVVLINSTIAHNPGLQSGIFQIKRSITIGPNNYQLWSYENIF

IGLMLIADGFVLYWRLIRRYKQMQLRHIISELHYIAAGHFDHRIDFNLTGDTQRVVESVNALVDSAIHSM

DEEREIERSKDALITNVSHDIRTPLTSIIGYLGLIENKQYQSEEDLIKYAHIAYLKATQMKSLVEDLFEY

TKSRQTETPLAINRLNLTAMLEQLAASFELEANKKHLKIEVESGTQPIYIEADAEKLGRVFSNLITNAFK

YGTGAKNVVLQAEQRGEEVVIHVTNDGQRIPAESLGRLFERFYRVEESRSKATGGSGLGLAIAQSIVDHH

GGYIYAQSDDEQTSFVIHLPVKHDHPLTKKQSDMIN

>gi|116334616|ref|YP_796143.1| Signal transduction histidine kinase [Lactobacillus brevis ATCC 367] lvis_2056 lvis220

MKLTTREKSALFMEGVVTVILLLMINLALLVMISQAIESNPGLRDGIFIIKRSVVFGPNHFQLWSWENVF

ISFLGLADILVVWWRLIRRYRQMQLHHIIDELHYIASGHFDHRVPFRVTGDVQRVVDSVNALVDSVIHSM

DEERAIEKSKDELITNVSHDIRTPLTSIIGYLGLIEDKQYHSQADLLKYTHTAFLKSKQMKSLVEDLFEY

TKVRQTDTPLSLTNIDLAAMLEQLAASFELEAQKKGMTIGASSTPSVLMMEADPEKLVRIFNNLVANALK

YGDGGKQINLIAKQINDNELEVRVTNDGPKIPKESLSMIFERFYRVEESRSKETGGTGLGLAIAQSIVAL

HGGYIYVESDDDLTSFVIHLPVKHDQPLAIPQRLKTTN

>gi|81427893|ref|YP_394892.1| Two-component system, sensor histidine kinase [Lactobacillus sakei subsp. sakei 23K] LSA0278 hpk31 lsak220

MNEYQVGQDFKKVQLTAKEKSELFAEGIITVILLLLLNLSIMVLLQQAIVNNPQLRGGVWMIKNAIAIGP

NGWHIWSWQNWFVILMGIADVLVVYWRLIRRYHQMQMWHVIGELHYIADGHLEHRINLRVNRDLQRVIDS

VNTLVDSTVRSMEEERKIEKSKDELITNVSHDLRTPLTSIIGYLGLIEDNQYQTKDELTKYTHTAYVKAQ

QMKVLVEDLFEYTKVRQTSTPIAKTTFDMEQMLEQLAASFELEAQKKNMQITVECQPTPLMMEADTEKLG

RVFNNLIANALKYGKGGKQIILSAEKVGQEAIIKVSNDGQQIPSDSLNQLFDRFYRVEASRSQETGGTGL

GLAITQSIVALHGGYIYADSTPQLTSFVIHLPLKLGRQIEPQNGAAKNS

>gi|170016614|ref|YP_001727533.1| Signal transduction histidine kinase [Leuconostoc citreum KM20] LCK_00256 lcit220

MNKLSLFFKHNRTKIYYAVGTVLLLLLFSWFVLSATVLLGQAAHIALRWRFWLSMLILILGDLVIVFWQY

VRWRQAESLIHLIRDLHQIAKENSDMTIDWHAHYAPRTQALIDVSNKIAKNTRKIREEERASERSKDEMI

TNISHDLRTPLTAIIGYLGLVEMNATTLSPEDRAKYIHTAYDKSNQMKILVEDLFEFSKTQARDAVLNLT

TISLGDLFAQLLASYEIEAHEHQIEMMQITNPELIILEADSDKLARVLINLITNAFKYGEGATYIKLTAQ

VKAQEVEIRVINNGVQIPQEALLEIFDRFYRVESSRNSKTGGTGLGLAIVKGIILQHGGTVRATSDAELT

TFIMTLPLKQSRGNRDEVD

>gi|184154584|ref|YP_001842924.1| two-component system histidine kinase [Lactobacillus fermentum IFO 3956] LAF_0108 lfer220

MLKLTGREKSSLFFEGLLTIILLGLINIALITIIQTMIQSNPGVQSGIFIIKQSLVIGPFHAQIWSYQRI

VMALLVLIDIGVLWWRLLRRYHLYQLDHIIGELHYIAQGHLDHRIPFRVNGNQQHVITSVNALVDTITQA

MQEERASEKSKDELITNVSHDLRTPLTSIIGYLGLIEDHQYQSEEDIVKYSHIAYDKAKQMKNLVEDLFE

YTKVQQHGAPVNLMTVDLGQLLEQVGASFELEADKKGMAINVSCEPTPLSITADPEKLGRLFSNLVANAL

KYGHGASYIHLTAKQLGEKVVITVADDGEKIPAESVKHLFERFYRVESSRNKATGGTGLGLAIVQSIVEL

HHGSVTARSDDQETAFVVTLPVKQVQKEVR

>gi|16804459|ref|NP_465944.1| hypothetical protein lmo2421 [Listeria monocytogenes EGD-e] LMO2421 lmon220

MKSKLELLFTKISHVMNVWQATFFAAVSWVLALITIQTIYLWSISIRDDSLFLREVSQAIVQLFGTNKYT

RIMQSFWMMSLKFLIIFLIACLFFAFFYRLMRQRILKKQLRTINFALNDGKPVDTESFVPEIQELERNIE

NMRERQKKLMQQEEQAQQARNDLITNVSHDLRTPLTSILGYLSYIHEDRYRDEIELRYYTELVYGKARHL

HKLIDDLFSYTRLDSVDYQLKKDELDIVELLRQLVAEYDGRAAERNMQIVEHFDANKLIIGGDGNQIMRL

FENLFSNALRYGEGNKQIDVSAKKEDNMAVVRVTNYGEEIPNVDLPYLFERFYKVDKNRTTTGTGLGLAI

AKSIVEKHGGIVTAESKKRQTSFIVKLPLI

>gi|126699229|ref|YP_001088126.1| two-component sensor histidine kinase [Clostridium difficile 630] CD1625 cdif220a

MKSKNIKVNPDFTQVRNKIFMRTALMAFIAIIFIWILYSFILYGKFSIWIVSFFENAFRMEYKAALNLYQ

QIFRNSMELFMILAMVIVFFIIFRVYLNWFTKYFSEINQGIDSLIKEDVGEVALSPELLAIEKKINSIKH

ILEQRKFETQMAEQRKNELIVYLAHDLKTPLTSVIGYLTLLRDESQISEELRKKYLSISLDKAERLEDLI

NEFFEITRFNLSNISLEYSTVSLTRMLEQLTYEFKPTLMSKNLKYTLNVTPNMMIRCDVNKMQRVFDNLL

RNAVYYSFEDTTIEITAVQEDDYIKLKFINHSNMIPEEKLERIFEQFYRLDTSRNSNSGRAGLGLAIAKE

IVELHNGTITAHSENNIITFKVIIPIVGNL

>gi|126701226|ref|YP_001090123.1| two-component sensor histidine kinase [Clostridium difficile 630] CD3599 cdif220b

MDIKLKNKSLLNHMFRPILDIIISCALTSMLILTSMYIIKSLYFNSGISVINQLVYQLRDLRSLIGESLF

YPFLFTITSLAIYSMISYKRNKQIVDFIRKTNRKNQKLEIIKSAVNLMDEGNLEKSIDIEYDLDILKDKE

KDDIDELAHKINNIVHQLRNITIEERQAQQTKTDLITNVSHDLRTPLTSIMGYLGVIEEGKYKDEVQMMY

YVDIAYEKSKNLNVLINDLFELTKMQNNTIKLNKIEINLVELLSQVVSQFEIYFKQEFMVSRINFNEEKL

IVKADPNKLVRAFENLITNAVKYGKDGHYVDIVTEKKEDMAVVKVINYGEPIPVLDLPNIFDRFYRVEKS

RNRNVGGSGLGLAITKNIVNLHDGEITVSSDKYQTVFEVQLPIIQSA

>gi|116494022|ref|YP_805756.1| Signal transduction histidine kinase [Lactobacillus casei ATCC 334] lsei_0461 lsei461

MDKLKQRLTRLPIKKALPHLILIGTIAAALVVLVYLLIWHVVMMPIATALAKRWFIGGMSLYFGGAIIGS

FAMLYAVMNVTARLFYRVKLGPPLDQLQYGVTHIQAQDLDFTLQPTSDDELGQLVAAFEEMRQALRKALE

TAWQLADDQKQVNAAFAHDLRTPLTVLQGNLELLTFNEEGQPPDQHLLDEMQRQLDRMNNFIKTMSHVTS

IQNIRLNHHAITEAQVEQQLRSEVDKLPTAKTIGWQVDSTRLTDNKLSVSPDAILEVFDNQINNALRFAK

TKVGIHLTFNDQQITLQVCNDGQPLTATEQRKVKLPYFSNDKAAHHLGVGMYICNQLCAAHRGYFKIQNR

EHAAGVCSTAVFGYFDGSAES

>gi|126698062|ref|YP_001086959.1| two-component sensor histidine kinase [Clostridium difficile 630] CD0485 cdif461a

MSLKKAFFVLTLFSLIIASVLTTFAYILLNNLYNSIQDKYLKTPINTGIITIVQNNVNQFSDYDKTLLNI

INILQLVLPLIFFIGLLLLADIIFYKVKLKTPIEILNKGAMEISNNNLDFCLEYNNNDELGNLCNAFEKM

RSELNKNNIKMWTMINDRKQLNAAFSHDLRNPLTVLKGYSNYLTKYIPTGKLSDEKILSTTQLMSEHINR

IEYYVENMSNAQRLEDLVVSKSMSNINKFIENLDENISIIAKQEGKSFTLKNQINNINLFFDENIILRVV

ENIISNAFRYARNTVSILIYLEQELLTFVIEDDGIGFSKESLKLALRPFYRDKTLNDSNAHFGMGLYISK

ILCEKHGGSISIENNSTNSAKITVKFSTRN

>gi|126698059|ref|YP_001086956.1| putative lantibiotic resistance two-component sensor kinase [Clostridium difficile 630] CD0482 cdif461b

MKNQSLITQFRHTFIFIIIASIVATVITYVFALYLYIHSLNKDIYPPNYYERQVPRIEKYINEKNITLLS

QSNEEGLKRTIRGDDMLYQVVDNNGNILYGTNPKKLFKTKEELFNNFINKTVRKGGYIHTVPIKGDNGKI

EGAVILFYQVKITFANIRGRFVFAVIIMALFSPFLYIVGFTRWLSKRFVKNINQPLHLLIDASKKIKEKD

LDFEIDYYSDNELGKLCSAFSEMKDELKGSLSAQWKMEQERVEMVEALAHDLKSPLSIILGYTDALIGNN

TDDNEKLHRYLTVIRENTEKSAALVQKMQYTSDLEKSNIQLNLVPINLPEFLRQKVQDYELQAHQKEVEL

ILKMQGNIQSPIQIDVDRLTRIFDNIISNSLQYTPSGGNISITVKDEKNCISYEICDSGRGFSSKDLKKA

LDKFYRGDEARQTKGGHSGLGLYIVKQLVEQLGGSVKIENSKSGGACVKFWHSI

>gi|56965314|ref|YP_177046.1| two-component sensor histidine kinase [Bacillus clausii KSM-K16] ABC3552 bcla461

MNKKRTSIKQQIRLTIAKTILLSFFATIIVVLASWAIVMSIDDRYYANYYESQVPQITDQVQSLNRELVN

SQASGAKQLEEIIPTPGMEYQVLDEQGTFLYGSFSANVNPEGVHLLNKVNKSFLEENTATSFIPIVDQNG

TFAGSVVLQYKLESEVEALFNIFFVCLFLTPFFFIMIFTYYYASKLGKNYYARIQKLIVATELVRKQNLD

FEIEADGNDEITKLSTSFNIMKQALQDALYQQWNLEKNRSEFISSVSHDLKTPLTIMKANAQMLERKIKD

EQHKTYIANMIGEIERTEKLVQELGTKMNDEESFFAIETKTVEPGHFFKTEFAKYKDFVQNEGLTFHTSV

LDYRKTANTIQIDVQKITQVIDNLISNSLRFAEPYDGHIDCHAECHDDYVKVTVSDNGKGFTESDLHYVF

QKFYQNNDSADPHKGLGLYIVKHIIEKHGGNVQAWNDKGAVVSFQLPAIPKNSH

>gi|116494260|ref|YP_805994.1| Signal transduction histidine kinase [Lactobacillus casei ATCC 334] LSEI_0712 lsei712

MNAEKGIKHQQRRLFIGQLLSFAGLFVVLGIIVFFLYERSIYQDIDHTLEQQQAMILNPNEETTKLGPQQ

PGTRVTHPAPFRTNMVVFNQKGRIINQAMLGERFYAYFKNLKLDRSAQNKLQTLTTSTGTFRTLLIKAPK

YSADPQYAGHYVLILQNTDAQEAAIRSFRQVLIVTIILFWALALGLSYWLSTRAMRPIVRSWQRQQDFVA

DAAHELRAPLAVIQSKQEALLTKPQATIIDESEAIATSLGETKRLRQLTDDLLTIAKADSNTIEIELVDQ

PFEKAFEPQLSSYQDIAVSQQKSFTTTLPKAGHALFDTARMKQLLVILLDNAFKYTQAGDSIWVEVTQTP

RDWQLQVGNSGPSIADADKARVFERFYREDASRSRQTGGSGLGLAIARWIVTMHHGHIEVKDVHPHGALF

IATFPNKPRMR

>gi|116490258|ref|YP_809802.1| Signal transduction histidine kinase [Oenococcus oeni PSU-1] OEOE_0143 oeoe712

MSKTKINSKQYIKLFLLEAIGFLMIFSFVGSYIYFTYTRTVQRNADQILNSMVLRIQKSFRSGSKQLMIP

GLVGQGQGSTTVGGTPSASSPQEGSLLKSNVSGQDTIYYSKSGKIVNSYSLGLRIWTYENLYKKISGELN

NIQTIKLSGSYFRVRLIKLKKPIFLIYGANTILGTAKYAAVVYNFDTERSNMDSFRSVLFWTLMFAGMFS

LVVSWLVTLRVMRPILQSWKQQQEFVNNAAHELRTPLTIIQNKMEGLLRKPTSSVADVSENIVVSLSEVR

RLNQLTSDMLTLARTGSNMTQLDPKMVDIENFIKEVIEPYSEIAASKKRKFAEEIDIKGKMVIDTRRVHQ

LIVILLDNALKYTEAKDLIKIKARREKNNLVIEVADNGRGISAEAKKHVFDRFYREEKSGNRSTGGTGLG

LSIAKWIVNAFQGKISVSDNSPKGTIFKVVLPQLKLNK

>gi|116618898|ref|YP_819269.1| Signal transduction histidine kinase [Leuconostoc mesenteroides subsp. mesenteroides ATCC 8293] LEUM_1809 leum712

MPNAINKKQQVRGFIWLLASFFVIFSILASVIFVSYTRTVFNSADSAINQTLSMYDSNGLLSTSDKDVKK

PQPLYNNTSTRTETWLFDKNGKLIIDSNTEDTRQAALQKALQQAIKFDKATMTVKPNTYKFYGNYYRIIG

FQFKKNAAKGYNQIAVHGMVTVNVTDTMDNLNNFKKVLFWSFGLFGILLVIVSYLIARRNMKPILKSWQQ

QQDFVNNAAHELRTPMAVIQGKLETMLTKPESTVRDQSNAIILSLSEVRRLNSLTNNMLTLAKTGSNMTT

IEKESTDVKQFLENIITPYQEMAEFEEKAVTLEVDVNQAIFIDQKRIHQLLVLLLDNALKYSDAGATIAV

KAGIEKKKFVLTIADSGRGISDEAKKHVFDRFYREDKTGNRQTGGTGLGLSIAEWVVHAHGGKITVADNQ

PQGTVFKVILPL

>gi|116333024|ref|YP_794551.1| Signal transduction histidine kinase [Lactobacillus brevis ATCC 367] lvis_0355 lvis712

MSNKVNRAQRRRLFLSSFIGFTIIFILLGVIVFVLFRRAILTSTDQALRVERSARLAGPPKKQRLQKPFG

QATRDLQRGQQQTTHPFMTTTVVFNRNGKILNRSQLGSRYDTLKTLTLNKSETGKIRSVVVNGKYNFRTL

LVKIPKDNGDKTVAGHYLMILENIDPQKAAMSSFTRVMLITMGIFWLLSILMSVWMSRLTMRPILRSWER

QTEFVGNAAHELRTPLTIIQNKLELLLTKPNDKIIDQAENIAIANSESQRLQKLTQDLLALARSDSNTVQ

TNFEVTRLARFMTDIVEPYSEIAASQGKSLSLTPVTDFKATLDQNLIHQLMNILIDNALKYSPQGRAITI

SARLIGRKWQLIVADQGIGIQAGDRRRIFDRFYRVDTSRSRQTGGNGLGLSIAQWIVGLHHGTIVVKDNL

PQGTQFVATFPLNPSASLTGRRDKKQ

>gi|170017834|ref|YP_001728753.1| Signal transduction histidine kinase [Leuconostoc citreum KM20] LCK_01485 lcit712

MPNVINKQQQIRGFLLLIASFFVIFSVLGGVIYWSYTRTIFQNSDSAITQQIKQFNLAGALRDSEDTNRK

SPMLLQSNTLADVWVYDKDKHLLLDDRIPESLQEIYRKQFKYRGNFISSRPKTVQIEGNYYRVASVEFID

NTRNNSGQLVKYGIITVNVNDTIFNLNHFKKVILWSFGTFGLLALMVSYWISLKNMKPIIKSWQQQQDFV

NNAAHELRTPMAVIQGKLENMLTHPESTVREQSNAIILSLSEVRRLTSLTNNMLTLAKSESNMTRIEKEP

TNIADFLSRILAPYQEMAEFDGKQVLLSAHVNQPIYIDQKRIHQLIVLLVDNAMKYSEAGATVAIAASIE

KRKFVLTVADTGRGISDEAKKHVFDRFYREDKTGSRETGGTGLGLSIAEWIVRAHGGKITVADNQPKGTV

FRITLPL

>gi|22537144|ref|NP_687995.1| sensor histidine kinase CiaH [Streptococcus agalactiae 2603V/R] SAG0984 saga712

MSKPIKISKFKKNVSDSHFIHFFTVFSGIFLVMTVIILQVMRYGVYSSVDSSLKYISTHPKNYINMVMSR

TAAYLDNSNIASVKLKPGGQTVANTDIILFTSEEEVINYFDAFSNYQFLKPNKKNLGGISELTLTNIFGQ

DETYHAVTVKVNNPAYPNVTYMTAIVNIDQLVNAKERYEKIIIFVMTTFWIISIGASIYLAKWAQKPIIE

NYERQKAFVENASHELRTPLAVLQNRLETLFRKPNATILENSENIASSLDEVRNMRILTTNLLNLARRDD

GIKPELAVIKPTLFDSIFENYDLITQENGKNFTGHNMIQDSFKTDKTLLKQLMTILFDNAIKYTDNDGSI

DFTISETDKYLFLEIADNGPGISEEDKVRIFDRFYRVDKARTRQQGGFGLGLSLAQQIVNSLRGNITVID

NKPRGSIFKIKLPKVKG

>gi|24379560|ref|NP_721515.1| putative histidine kinase sensor CiaH [Streptococcus mutans UA159] SMU.1128 smut712

MLIKLRKQKQDENISFFSHFFAVFTGIFFVMTVIIIQVMHFGIYSSVDNSLKIATENVNDYVNMTLSHNN

PITEAENGDASFKVKSNGQMAANTDTILYDKKHNVINNVNAFSSLYHIRPKLKDIGDIVEQEAKNIYGQT

EKYRTVTVKVNNDFYPQIKFATIVINTTQLEEANTRYVTIIISVMIVFWLISVIASVYLAKWSQKPILEN

YEKQKSFVENASHELRTPLAVLQNRLESLFRKPEATILDSSESIASSLEEVRNMRMLTANLLNLARRDDG

IKPEITEIQPRFFDDTFKNYEMIARENGKKLHIINKVNRQIKTDKTLLKQLMTILFDNAVKYTDNDGEIW

LDVKTTDRSLIFSVADNGQGISDEDKKKIFDRFYRVDKARTRQKGGFGLGLSLAKQITDSLKGSIIVKDK

KPQGVMFEARLLANK

>gi|125623602|ref|YP_001032085.1| Sensor protein kinase kinF [Lactococcus lactis subsp. cremoris MG1363] llmg_0748 llac712

MKNIIKKVKSSAIVKNDGKNFLHFFLAFTVIFVALAVIIIQVMQLGMYRATDQNLHNLAQNRVFLEEAAN

NQFGTSDSQDSGFYGPNNSVIFYDSEGKSFMPSSESNGRISTNFVMSRLQKAMKFNEKQINTIQTVSVKN

PYGDNWHYRYLTTSQFIITNSDGTVTPVYVQIFSNVDQIQDAMSRAMWVIVTTMITFWILSVIISLYLAN

WTLKPILAAYEKQKEFVENASHELRTPLAILQNRLELLFQKPTATIIDESENISESLSEVRNMRLLTSNL

LNLARRDSGIKIEPEPTTATYFENIFNSYEMLTENAGKKFSGNLKLEGTVNLDQALIKQLLTILFDNALK

YTDSEGEISVDVIKNGGFLTFAVADNGEGISDEDKKKIFDRFFRVDKARTRQKGGLGLGLSLAKQIVEAY

NGKITVEDNKPKGTKFIVKIRVDSTISNPAKIFQKL

>gi|126699295|ref|YP_001088192.1| two-component sensor histidine kinase [Clostridium difficile 630] CD1689 cdif712

MINKNVFTSTKNHLIKMYIIVVGSFLIIFSIFIYSYFRGLTYSGIDSEINDELEYIVSQFKRTSFLNPIR

LKDPKDMVYVYEDGRISYYTQNEYFDELLPDRRLDKKNSFFKYTENGYTFRELNVDVGRYQIQIIRNIDS

EMNSLRQLTSVLIIGILISVIITYFVAVYLTRKALIPIETAWKNQAKFIQDASHELRTPITIVSSKLESM

LKSPESTVNDEVETIATAMKETRRLKKMITDLLSLTKEDSIVKVNLEEIDLEKLLEEISEDYIDIAEFQE

KRFVFNSKLKNKVIITDKNKLRQLILIFIDNAFKYTKLGDEIALELKEDIEDEVTLLISDTGIGIKKEEI

PLIFDRFFRSENVRNKDLEGSGIGLSIARMISLNLSIDINVTSDVDIGTTFELSIPKKLK

>gi|116494442|ref|YP_806176.1| Signal transduction histidine kinase [Lactobacillus casei ATCC 334] lsei_0935 lsei935

MNKRIIRRALIISLLSLAGFFVLVTTVNQQIVHQQARDLRRVGRTYAASSHDGVNRQALAESEAAFITVI

PNNPRTTRQKVMADALRGNRDAAFVTTVNVNGRTEQAYLFQNKGQWVAVSRFKSSVWTTAPEQFWLLTLA

FAALLAAILIWLFRSEHRYQEAIEVMSHNLDRIRRKKDPDPVILAPDSPFVPVARRINALAAEQAHLREK

VAVRQSSFDRLIDNLPLGVMLIDTDKDVILHNHAMSQLLGHQIPQPRHSYLDDVKTYALARMIEHTFRHE

KTHHQELTILTTQKSVDASVIPLNQGISRLQVLVILYDVTYLRQVEKMQLDFVGNVSHELKTPVTAISGF

AETLLGGAKNDPATLDRFLGIIYDESKRLTQLINDILTLSRPDSNQNVAASVALKKLVDDALHNLAKVIQ

DKQVRVEVAITPDLLVVTDERKLTQIVRNLLNNAVFYNRLAGHVTISAQVVDHQLSLAVADTGIGISETE

QARIFERFYRVDKARSRHNGGTGLGLAIVAELVKSMDGHVAVHSQVGVGSTFTVTLPVGEGPHRG

>gi|116492235|ref|YP_803970.1| Signal transduction histidine kinase [Pediococcus pentosaceus ATCC 25745] PEPE_0432 pepe935

MSSSRKKAIKRTVQFGFASLIGNAFMAFLVYKLIVKENPRVTKVDMSLLIAVILLITVIEIIVFWSNQKK

IIERQKLFRKNLEEVIKGNHVPKIQLDPEDPFYKLSKVVNEIGTHERHQIHVLRNQQSELTAIMDNLPVG

VLVINRHRELQVANTYAINHLGITGLDVPHPYTMDIRDHELSELVDNVFRTRQSTTKTIKLDGDQPYIYE

TTVVYSPKVHHKFEIIVLLYDATETVRSKQIERDFVNNASHELRTPITSIAGFADTLLNGAKDDPETLDN

FLRIIKAESDKLVQLTDDILTMSRVKSGERERKEINLHKYVDQQLKLVESNISSMNLHVENNIPADVNEE

VIENDLFQIVKNLITNAISYNRKGGKIKIDYGSHHDRWKLVVSDTGIGIPLGQTDRVFERFYRVNNATKG

GTGLGLAIVKEAVDDMNGSVRIESDEDSGTTVIVKFKRDIN

> gi|116617704|ref|YP_818075.1| Signal transduction histidine kinase [Leuconostoc mesenteroides subsp. mesenteroides ATCC 8293] LEUM_0588 MODIFICADA leum935

MKKRIGKITSIYSLNILLLFIVTRVIHVSHNQLYILVIIFFVVANFEVWFLNFWQNKETA

ELTIMKNRMIATSAGETPRGVLAEPTSPYYRIIRTFNELQSYVRHSQYSANREINNYQTL

LASLPVGVINVSRHHMIDVFNQTAADMLGVEMPEVPILDSLVIRQFTLSEIINHTFNTNQ

RQHSILNLMIDGEARQYDVSTLFHQSGSNAEVMVMLYDLTEVLKLERMQADFLANASHEF

KTPLTAITGFVETLQGDAGADEETRTQFLQIVADEAKRLSALVNDILSLSRMQHKVDEKI

SNLEISAIVEQQLEKIDTKTITVHNDIAPDFSVSGIASDVSTIVQNLLTNSVKYNKIDGE

VWISAYKDTQQWQIKVSDSGIGIPLNQQSRIFERFYRGDESRQRKIASGTGLGLAIVNEI

VNQHNGEINIKSQVGVGTTISVILPL

>gi|170017611|ref|YP_001728530.1| histidine protein kinase; sensor protein [Leuconostoc citreum KM20] LCK_01261 lcit935

MKKNSLKFLSFLCVNMSVILLFSVLLFHLSVKQWLLLLLAIFLATIGEYAVSLFWNGRRQREIDVMTNRM

LATQNNEEPRGVLAEPQSPYYDLINAFNQLQSYVKHMQSTMQRDLANYQSLLTSLPVGVIHVNRRHIIDV

FNQTAADLLAVDRPETPIAESLVIRQFALSELITQTFNTQQNQQAILNLAVDNGMKQYEVSTFYQKGDVQ

HAEVMIILYDLTTVLQIERMQSDFLANASHELKTPLTAITGFIETLQGPAGEDATTRQQFLQIVSDEAQR

LSLLVNDILSLSRAQQRDDTLQKELTISDMIVKQWEQIGTLYTDKAITLRNDVPRDFVVHSIYADVNTIL

QNLLVNAVKYNVQGGSIQVTAFKDHQHWQLNIKDTGIGIPTSQQSRIFERFYRGDESRQRKIANGTGLGL

AIVNEIVSKHNGTIKVQSQVGVGTVMSMTMPL

>gi|28377604|ref|NP_784496.1| histidine protein kinase; sensor protein [Lactobacillus plantarum WCFS1] lp_0744 lpla934

MHNRWQQTTRLLLMENVLLVLIVLVVLQHFSPIKWSWRLVGCLVGAWLVLAVIEGLIAWRQQSERQRTID

RMEAKLRQMSSQQFPPHILLPQSDPLYSLAQAVNQLESYQKSQTRRAELQEKELMMIMRYLPVGVMVIDH

RRQVQLSNPAMSELLQVSVNTVAHIYTKDIQLYALTKLIEDTITSGKNQRATITLIPAPNTQVVEASTVY

IQGNRSQFQIVLMLYDITEVYMIEQMQDDFVSNASHELKTPITAISGFTETLLSGAKDDPDTLDQFLHII

ADESQRLIDLIQDVLSLSRIRAQNTTMLTLAPLAVRPLIEQELAVVQQAIERQHLTINNQVDADLMITSD

SQKLSQIIKNLLTNAIKYNRPHGSVTIATMVSHQTWSLIVKDTGIGISADDQQRIFERFYRASPSRSQQL

VSGTGLGLAIVQELVNAMHGTIDVVSQRGVGTTMTVTLPLVASASEQ

>gi|116333281|ref|YP_794808.1| Signal transduction histidine kinase [Lactobacillus brevis ATCC 367] lvis_0632 lvis934

MIRRLGEAFVISGVVNSFLVWAVQPAHLEPALLIAWSLTLLETVVIGYGWRWAERRITILTKKVEGIRHA

ETPAHVLLSPRDPFYRLALQINYLQTHQRKVQRQVAGQNQEFATLLDYLPIGVMVIDRYRKVELANPAME

QFLQQPISPRRHPFTQDVRQFELASLINSALSERQTQHQTLKLPGSPHERTVDARAIYTATSQDYFQVLV

LLYDVTEVYAVEQMQMDFMSNASHELKTPVTAISGFAKTLLAGAKDDPEKSVEFLQIIDQQSERLVELIN

DVLTISHIESGNNVEMTTVPLGHLVASQVNLLTPLATVNHITLHNQVASELQVTTDQRKLDQILKNVLSN

AIKYNRPQGRVTVTATVTAHDWQLIVQDTGIGIAPQDQQRVFERFYRADVSHSNQLVSGTGLGLAIVREL

VQSLNGKIDLQSELGTGTTVTMTFSRQ

>gi|81428114|ref|YP_395113.1| Two-component system, sensor histidine kinase (possibly involved in phosphate regulation) [Lactobacillus sakei subsp. sakei 23K] lsa0501 hpk2 lsak934

MTKHDQVRFYKLATILVAIYIAFILLINLVIYRQQSATLKERRADYALIVKDADFKRQKWLQQNKLQVVT

SQSPQHTKLERSVADVLNRDHTTRNFSVRQKIDGRWYLVAVHQSSQPTFVLLMPQQRFWHTWPEIALTVT

VLYLLFSLIVLWQFKRQRSHFYEHLKILVANIHHIRYEETPEPIIFQKDASLYVLAHEVNQLNKDMRHMR

QKIAIQQGSFDRLIDHLPVGVMVINENRQVVLHNEAMGQLLETAIEPQKHPYIDDIKTYELTRMIEHTFR

YRRSHHQEIQLIQNRERFVDANVVQLNTVKSKFQALVILYDLTDIRRVEQMQLDFVSNVSHELKTPVTAI

TGFAETLLAGAKDDPETLQQFLKIIYEESTRLTALIQDILALSHLDRQDQTQAQDIGLKAIVEANLELMQ

QKIQQKEIHVSVNIPETLTFKTQKMKLNQVLKNLIANGITYNKQAGSLVIAAQVDHQQLIIRVVDSGLGI

PSDLQERIFERFYRVDQARATHKSGTGLGLAIAKEITESIGGQLTVESQLGVGSQFILTFPLT

>gi|90962156|ref|YP_536072.1| Two component system histidine kinase [Lactobacillus salivarius subsp. salivarius UCC118] lsl_1181 lsal934

MSKIRLVLLNIVLVLISMLGAVLIMVDFKKSVLSEFLERESWIQIIIAVVVLVVLDTGIDLWNSRKQRKE

IERLNKKIIEDIKLTDNVIDKKNPYYELSQSIGYVQDKQNKLSKKYDKRKREYLSLLEYIDIGILVIDYK

NRLTMSNELGKDFFDFDNEWKNQDYQNYIYNLDVRKLIEQVKLTKEDKKKQIVLKIDTVQKIVETSAIYI

PIDSKRYLIMLMLRDLTAQKEMEKKQLDFVGNLSHKIKTPITAITGFSETLLDGALDDKEVSKQFIEIIH

DESTKLSNLVDDMITISRLNEHGSLNIELVNIFKLVKDTLQLFSHEIEKKGLNVRIEIDEELSLYCDKLK

LKHIITNLIQNAIRYNLENGEIKIKVQNDKKNEWVFVISDTGIGISDKDKERIFERFYRVDKSHSKTVGG

TGLGLAVVKESVDALSGRIKVKSQINKGTTFYVILPRFKAR

>gi|27468286|ref|NP_764923.1| alkaline phosphatase synthesis sensor protein [Staphylococcus epidermidis ATCC 12228] SE1368 sepi934

MLKFHLRLLLLISTITIISFIGLGAIIHNTIYQTLTSNQIKSLDSEARNYVNLFNNNKEKEITNIAHNEK

NIILIKEKDKDKIIYSSGNIKDIDHRIDNEANPSKLINKNTKLGMRYTYKNTIDDKTIYISGINNEIIDL

QKDLWKYLSIVGVIVLFTVYLASRSINRTYIRPINEVTYATSLLADGYYHVRVPESNVKETRALFVTTND

LARRLQKLNNSQKIQSNRLKTTLENIPSSVLMIDKHGEIVVANHAYYQVFNPDQMVENKSYIGFIDDSIE

KLIIESFRTEKVIYEQLEVAINNVHTKYFDVSCIPILTKSKKNLQGMVVVLHDITNLQKLENLRREFVAN

VSHELKTPITSIKGFAETLIEGAKNDEQSLDMFLNIILKESNRIESLVTDLLDLSHIEQQKELEINYMNL

SELAINIIDNLQTQAYNKRIKIQSEIEKDVIIEAHENKIAQVITNLLSNAINYSSEDNKVIVRVYRNDNK

VYLEIQDYGIGISETDQKRIFERFYRVDKARSRDSGGTGLGLSITKHIVEAHNGRIDVKSAPGKGSIFKV

LFNDN

>gi|16804538|ref|NP_466023.1| two-component sensor histidine kinase [Listeria monocytogenes EGD-e] lmo2500 lmon934

MKKLWLKIGLSFFILFFVVMVIVGIFSGELMKSTYLNMKENQLEDDAKILLQTTNMENLDLDKDAATIQK

SLDPLGEDIDARITVIDSKGDVVADTKKDPEKLDNHMNRPEVTDILKKGESVGISIRESDSLGYSMLYVA

VPVKHQSKTDGVLRISISLESVDAAVAKLWGNLALIFGIALVIIAAISVFIARKITRPVREIIEVSTDLA

NHKYDSRIHGKISGELQDLSISVNTLAESLETQMFEIKQNEQRLNAIVQNLVSGVMLINVDKQVIMTNRT

MYQILGETEITGKPFYEVIKSFALSQLIEATFETKTIQQKEIILYFPREMILDASVSPILGENGEITGII

LLLHDITQIRHLENVRSEFVTNVSHELKTPVTALKGFAETLLDGAMYDEMLLKKFLTIIKEESDRLHRLI

MDILALSRIEQNPVPENVELVEVDEVIEQSARTIFEMATEKNIQVIIPEKTIPSVTIETDRDKLQQILIN

LLSNAINYTPVDGKVEVKLIEQEAEVIIEVTDNGIGIPAKDIDRVFERFYRVDKARSRHSGGTGLGLSIV

KHLVENCGGRIEVESQEEVGSTFRVTLPKKA

>gi|30022663|ref|NP_834294.1| Phosphate regulon sensor protein phoR [Bacillus cereus ATCC 14579] BC4588 bcer934

MNKFRSRLLFTFVSLIVFILVGLGLLLETVFENYYIDHAKERMVKETEYVATLAEEQGFDNVLKNPYVFE

KLEEKIPASIVFLDEKKKVQYRKGQELAFSPETIKELSSEVAKQRSKVITKETDRKNEFYHAVFVQDVEG

KQGYILVKSIIDPLRDVHQKTWGLLIIGFVIACLVVVFLGMKITGQYIRPIESVTKVAIELAKGNYKARA

YESHSDETGMLSKAINILARNLQEMTLEQEMQQDRLHTLIENMGSGMILIDSRGYINLVNRSYKETFHVT

DEEYLDRLYYESFHHTEIIELVEEIFMTEVKVRKQMLLPLGIERKHFEVYGAPIIGTNHEWKGIVLVFHD

ITELKKLEQMRKDFLANVSHELKTPITSIKGFSETLLDGAMDNKKFCEHFLHIILKESERMQGLIEDLLD

LSKIEQQGFKLNMGTVDMKGLLEDIHMVLDNKAGEKEISLQVNVLKRASVIGDPSRLKQIFINLINNAIV

YTPAGGVVSVELAEDKYNAYIKVSDTGIGISKEEIPRIFERFYRVDKARSRNTGGTGLGLSIVKHLVEAH

QGTITVDSEVGEGTTFTVVLPKSATEK

>gi|116513987|ref|YP_812893.1| Signal transduction histidine kinase [Lactobacillus delbrueckii subsp. bulgaricus ATCC BAA-365] LBUL_0873 ldel873

MFKKIFRASFLLTSLVLFFAVLLSAIVLSIDFDHTEMSALASQADLAASGVNTSGKKYIKNLKGENYRIT

WINSKGKVLYDNEENPKKMGNHSRREEVAEAMKKGTGTSERYSTTLSTVTMYYAERLNDGGVIRIAITRN

SFWRILVRFLSPLLLIMLLSVLVSWFLAKTISQTIVKPLNEMDLDKPLESNAYDEIKPLLTRLDSQNKQI

ADQMEQLHHREKELKTVTDAMLEGLILIDLDDQILLSNPAVVSLLGLHDTDPLPDDVKEIIDKNSRLAHA

EGIVERNGRNIQISASPITSRKILKGTSILVVDVTKEYHEEQVRREFTANVSHELKTPLQSIMGSAELLE

NHLVKEEDRDSFYKKIHESSAQLLTLIDDIIRLSHLDENQRFEEIKLNLKSPVTEALEALQSSADRHGIK

LETNLSDTMILANFRLVYEIAYNLIDNSIRYNKEKGKTKITVKEHRGQAILQVADTGIGIPQEAQNRIFE

RFYRVDKSHSRKTGGTGLGLSIVKHAVQQCHGTIRLESELGKGSTFTVIFPALPAGADDNSK

>gi|22538096|ref|NP_688947.1| sensor histidine kinase [Streptococcus agalactiae 2603V/R] SAG1960 saga873

MTKKIFRTTLSASLGIVLVTILMIMGFLYNYFNHIQREQLRTQTALASQGISFEGKDYFENLKTSNVRIT

WVDNKGQVLYDTQSDAKHMKNHANRQEIKEAIKSGYGESTRWSATLTEKSIYAAQRLNNGTIVRLSVAQQ

TIFYLLLGMISPLAIIILLAIILSVLIARYIAKKVSEPLNNIDLDHPLSNDSYEEITPLLRRLDSHQAKI

QHQKLLLQKRQKEFDTIISKIKEGMILLDDQARIVSINAEALKLFQINDDWHGRFMMEVSRDLTLKDLID

QGLKGKKKEANIGIENNHYRVLVRPTTDNNRVTGLVVLLFDVTDQLQMEQLQREFTANVSHELKTPLHVI

SGYSELLANQMVPNEEVPQFAAKIHKESERLVKLVEDIINLSHLDEQEKLPQETVNLYDLTQKVLEGLQA

KADKKHIQINFNGEEAILRGNPVLLNSLVYNLCDNAITYNHEKGQVNVTLKNSPDTITLEVSDTGLGIAE

KDKKRIFERFYRVDKSRSKIVGGTGLGLSIVKSALDFHNGSIKVDSHLGQGTTMTVLLHKQ

>gi|125974108|ref|YP_001038018.1| multi-sensor signal transduction histidine kinase [Clostridium thermocellum ATCC 27405] Cthe_1599 cther873

MKKSIYKSMLVLALTTILLTSFLITGVMYRAFYLRMQQEIRNEAIFISSAYNLIGQEFFSSIADQESSSR

ITWVASDGTVLFDNMADAEKMENHLNRPEIADALKNGFGEAVHLSKTLGTQTFYWAVRLNDGTVLRVSAT

TNSVFKSVLGFFPYVALITLMVILLTMIIANLLTKKIFLPLNNLNLEDPLSNDVYDELSPLLIRMAKQND

QIKSQFKKLKEQKEEFNAITENIREGIIVLNNKGLILFINKSAADIFNVSTQDIINKHILTLDRSITLQK

AIETAMGGHLFEDIFAIGENSFNLLASPVKDEVVVKGVILFILDVTEKQSAEKMRREFAANVSHELKTPL

TSILGYAELMKSGMVKPEDISEFSDRIYNEARHLIDLIEDVIRISRLDEKNVQLPFEEIDLLELAKETVG

RLSSLAQQKRIKLSVGGDNAIIFGVRQILEEMIYNLCDNAIKYNYENGKVDVNVKTFSDQVVLTVADNGF

GIPREHQSRVFERFYRIDKSHSRETGGTGLGLSIVKHSAEFHNAKIRLMSKPGKGTTITVIFSREQ

>gi|116494458|ref|YP_806192.1| Signal transduction histidine kinase [Lactobacillus casei ATCC 334] LSEI_0951 lsei951

MVKHDYWAMPETKSVLRLTVWLIVILWAALLTVMVLVPVTRLWLVVIGLGSTLASSWVVLRWAVKFSQRF

DLALRQLDLTKAGASNYAIDRNDEGLFSDLNNRLYQYARQMQAERTAVKRDRDQLSVAITDIAHQLRTPL

AANNNLLEMMTAANWEVTRQELLAQHTRQAELVEQLILLAKVDTHTLSQTRQDVSVAALGKEALKPLLRM

VADNQLTIDWQVSPDLMLHVNPALVKEALVNVLKNAVEHTAFGGQIQIIGIADPIRVRLKIINTGSPIAE

ADMPHLFERFFRGQYATANNVGIGLAIAAGITRENDGRLSAENTAEGVQFTFEFFR

>gi|23097813|ref|NP_691279.1| two-component sensor histidine kinase [Oceanobacillus iheyensis HTE831] OB0358 oihe951

MIRNNEVRRYLFFAITILIISTVSIAFISSVAAGIVFVSTSLIIIVFLWMTHWRYKQMEKLSHYLRQISA

GNTSLDVRDNREGELSILKNDIYKMTLMLSEQGEDLKKDKQKLTDAISDISHQLKTPLTSMTVMADLLSD

ETMDDEKRLEFTHNIRKQLERMDWLVSSLLKLSKMDAGTIPFAMERIFVSDLIDEVIAPLEIPMELKDID

FQKFGNREVTFLGDSNWTKEALINIMKNAIEHTPEGEHITIHFQENALYTEIIVQDTGKGIPKQDIPYIF

KRFYKGGNATDGSVGIGLAMAHSIVTNQQGDIEVVSEEGKGTTFTLKFYKQVI

>gi|125973057|ref|YP_001036967.1| periplasmic sensor signal transduction histidine kinase [Clostridium thermocellum ATCC 27405] Cthe_0538 cthe951

MIKVSFFRNPEIKKGFLIFIVLSVVSTSIGFLYDKSVGFFVLCLCFVFTLVHFALTYLRYRKIAEFTREV

DRVLHGAESINFGSYSEGELAILESEISKLTIRLREQAEALKKDKTFLADSIADISHQIRTPLTSINILV

SQLLKPKLSDEHRIEIAKELELMLMRIDWLITALLKMSKIDAEVVRFQRKTVSVAELVRRAIEPIAIPME

LKEQQIDVFMEGNETFIGDLSWSVEAIGNILKNCMEHTPKGGRIVIKACENAIFTEIEITDNGPGIDRED

LPHLFERFYKGKNSGEHSMGIGLALARMIIVSQNGTIKAQNNHDGGAKFTVRFYKGVV

>gi|153941226|ref|YP_001390236.1| sensor histidine kinase [Clostridium botulinum F str. Langeland] CLI_0968 cbot951

MLRNREIRIFFIATLSISIVAIICIFMTNILGGIISITAFSMLIGCGFLFIKREYEEIENLSGYLRRISN

GEYSLDIRDNAEGELSILKNEIYKVTLMLSKQGELLKKEKVQLAKAISDISHQLKTPLTSMMVMADLLSD

NNLNEEKRIEFTKNIEMQLDRMQWLLTSLLKISKIDAGTVNFKKDKVVVSTLIEKAIKPLLIPVEIKEQT

LNIKGDSSVNFTGDLNWTIEALINIIKNCIEHTQEGGTISISFDENPLFTEIKISDNGNGIPKEDLPYIF

KRFYKGKNASEESIGIGLAMAKSIITSQNGDINVTSRKNEGTCFNIKFYKVTI

>gi|116494544|ref|YP_806278.1| Signal transduction histidine kinase [Lactobacillus casei ATCC 334] lsei_1042 lsei042

MKMLYQQLIGFFSVIMVTLVIVSILFIRSTTNTVWENGFKQLEQYTDVLKNNAVDENTYVINARFIQNAE

EILSDQHVHFVIYDANNVAKYPVSQKGQMPAIKASYWKKLKQGAAVAVPEMMTNPISGHAQSMTIYYQPV

FLSEKLLFVIAAFAPVEQIQSSLSKTEHNLLIAFVLSTLAAFIISYFIASYQVRRINQLRRATHQVAEGN

YDVEVKIKSRSHDEVAELADDFRDMVTSLKASQQEIQRQEDRRRQFMADAAHEMRTPLTTINGLLEGLAY

DAIPEESKGKSIELMRNETNRLIRLVNENLDYEKIRTNQILLSKQTFNARTDLDNIAEQLAQKSEEAGDK

ITIDAPADLPTYADHDRFVQILFNIVQNAVQFTKNGQITIKAKVGYHQTEFAISDTGIGMSKDQMKNIWE

RYYKADPSRKNTKYGESGLGMAIVHQLMGQHGGTIHVDSTLGKGTTFTLTFPDEATAPKQKQPGADQQTS

S

>gi|161507257|ref|YP_001577211.1| Histidine kinase [Lactobacillus helveticus DPC 4571] lhv_0791 lhel042

MKLIYQNMLGFLLIIVTTISIIGYSEIGYARNQAYMQNYQRMESYANSLGELAETDEKNGTVLLNDNFLN

QLEFVLRGDDVHLRIFNDQNMQIYPRVNAKVQLSDKIFSTLKNGQEIRIQNNHAEHSPIGSTKDAYTGVL

VPWMNGKNLVGVAWISSKVTHVERPIYMSKRNLLRALLVTVAVGLILSFIISYYSTKRIKRLSRATQKVA

LGNFNVQIAHKDSDEIDQLAENFNKMVKALKQSNEEVRAQENRRDQFMADAAHEMRTPLTTINGILEGLQ

YDAIPEESKPKSIDLMQRETKRLVRLVNENLDYEKIRNNQIMLIKTDFDAMPILRDLKSQLKQNAGKAND

EISVTGPEKLPIYADRDRFTQVMVNLVQNAIQFTHDGQIEISGKRLEHASEIFVKDNGIGMSKDQTKYIF

ERFFKADPSRARMGTGESGLGLAIVSSLIKQHGGKVKVDSEPGKGSTFTIIFYDKGYEQFISKDQ

>gi|116493099|ref|YP_804834.1| Signal transduction histidine kinase [Pediococcus pentosaceus ATCC 25745] PEPE_1353 pepe042

MKLFFQQMMAFLLVTLSSLLIVGLVFIQFTKDSVYRNKWHSLQEYADSIFQQAIVLDTETADVKSVQVDQ

LQGVEKLLDNQHIHFTIYTNDGNTISYPNRNIASRITKAQRRELKNGDTVKTKSRMTFDKSSRQVTMTEM

DKPYFNRNGNLVCVISIGSPLANVQADIEKVKKNLFMTFAFAIIVAVIASYFLSRYLVRRIQAIREAAHK

VTEGNFNVQLVSGRKDEIGELTNDFNIMTASLKESNDEIKRQEERRRAFMADAAHEMRTPLTTINGILEG

LEYDAIPEDSKEESVHLMQRETKRLIRLVNDNLDYERLRTNKISLNRHQINLKQATQRISKQLAKKAKLE

NDVIIIDMADDVMVYADTDRLIQILFNIIQNGIQFTQNGKITIRAQNAKDGTDIQISDNGIGMTEEQIKN

IWERYYKADASRIQTKGESGLGMAIVHELMQVHGGQITVESTPEKGTTFKLHFPEQQKKLGH

>gi|90962135|ref|YP_536051.1| Two component system histidine kinase [Lactobacillus salivarius subsp. salivarius UCC118] lsl_1160 lsal042

MKFIYQQMLAFLSIIIILSGTLAMVFFHLTKEMTYENAWQQMQGYAYTLRKEALKLTTDKAGKTIIVLNV

SQLKATEKLLSAQHTHFTIYTSRTDVLYPDNSVIRENRSISKADWKKLTAGNVVKRKQDFRPRKSKSEVD

GSDVKITDIYAPCFDYQGNLVAVIKVGEDLSNITQNLQIIRRNVVLTVAFAILVGVILSIIIAHFLTKKI

ARLRNATDLVAAGNFDVHLDTNGDSELDKLTLDFNSMVQSLRESNEEIKRQEERRRQFFADAAHEMRTPL

TTINGLLEGLAYDAIPEESKEKSIELMRNETKRLIRLVNENLDYEKIRSGQIELSKTEFDVVQVLNNIIK

QLRNKAEQVGDKFELYTPDKLMVYADYDRLVQIIFNITQNAIQFTENGVISISAEKGNEETIIRISDNGI

GMNEEQLRNIWERYYKADPSRKNTKYGESGLGLSIVHQLMQLHQGSVTVESKEGKGTTFTLRFPNKNKLS

KI

>gi|42518844|ref|NP_964774.1| two-component system histidine kinase [Lactobacillus johnsonii NCC 533] lj0919 ljoh042

MKLFYQEILGFLLVIITSIVIIGSSTIHFATIQAYSQSYTRLEEYGTSIGKLALAKDNPKHELDAKFLKN

LQVVMEGEDVNLRIFNNKNRQVYPETTMNWALPQKLFKQLKNGKKIRIRNDHEDGRVSSFSNKDAYTSVI

VPWRSKGKLVGVIWIGSRVQNVEQMIVREKHNLLTALLSSLVIGGLISFILSYYISRKIKLLSNATKKVA

QGDFNVHLKHKDTDEIDDLARNFNIMVNALKESNDEIKAQEKRREQFMADAAHEMRTPLTTINGLLEGFE

YDAIPEEAKPKSIALMHSETKRLIRLVNENLDYEKIRNNKISLIQSKFNATEVLANLLTQMKQKAAQKND

ELILNCPQQVEVYADKDRFTQIMVNLVQNALQFTENGKITISAKRIEHGAQFKIADTGIGMNKKQMKYIF

ERFYKADPSRAKIGSGESGLGLSIVLSLIKQHGGKINVDSEPGKGATFTVTLYDKGYKEFIKD

>gi|116629892|ref|YP_815064.1| Signal transduction histidine kinase [Lactobacillus gasseri ATCC 33323] LGAS_1260 lgas042

MKLFYQEMLGFLLVIITSIVIIGSSTIHFATIQAYSQSYSRLEGYGTSIGKLALAKDNPRHELDAKFLKN

LQVVMEGEDVNLRIFNNKNRQVYPETTMNWALPQKLFKQLKNGKTIRIRNDHEDGRVTSFSNKDPYTSVI

VPWRSNGKLVGVIWIGSRVQNVEQMIVREKHNLITALLSSLVIGGLISFILSYYISRKIKLLSSATKKVA

QGDFNVHLKHKDTDEIDDLARNFNIMVNALKESNDEIKAQEKRREQFMADAAHEMRTPLTTINGLLEGFE

YDAIPEEAKPKSIALMHSETKRLIRLVNENLDYEKIRNNKISLIQSRFNATEVLTNLLTQMKQKAIQKND

KLILDCPSEVEVYADKDRFTQIMVNLVQNALQFTENGKIKISAWRIKHGAQFEIADTGIGMSQKQMKYIF

ERFYKADPSRAKIGSGESGLGLSIVLSLIKQHGGKIDVDSEPGKGAKFTVTLYDKGYKEFIKD

>gi|104773777|ref|YP_618757.1| Two-component system, sensor histidine kinase [Lactobacillus delbrueckii subsp. bulgaricus ATCC 11842] Ldb0689 Lactobacillus delbrueckii subsp. bulgaricus ATCC BAA-365 YP_812689 LBUL_0622 lbul042

MKLIYQHILSFLIVILTSVMIIGFSTINYQKEQAYQSNYTRLESYAENLSTLQLYSKKTQSKNSPPFFSA

DFLQKFSFVMRDENLKLFLMDKNFKTTYPLGGYDILKKSAKSMLKKGQVVRIKDDHSSEQQDLAPKVQGR

VKRDGLRKRINRLFAAESYTWVVVPQVQGSGSNKKVVGAIMIGSRVKDVLQTVSQAKANLARALVITFVV

SLILNFILAYYQTGRIKKLSLAARKVADGDLDVQVDHRHADEIDDLAENFNEMVRSLKASNEEVKAQEER

RDQFMQDVAHEMRTPLTTINGLLEGMKYDAIPEESIPESIDLMSRETKRLIRLVNENLDYEKIRSGKIQM

MKSQFNAWTIMNDVQQQLTQNAAKNGDKIILDAPEDLPVYADQDRFKQMLVNLTQNAIQFTNNGTITLRG

RRFQHGATFSVQDTGIGMTQEQSKYIFERFYKADPARARYGGTGESGLGLSIVLSLVRQHGGEIKVISKP

HEGSTFMITLFDQGHEKHYDKKDKKEYKDKE

>gi|58337061|ref|YP_193646.1| histidine kinase [Lactobacillus acidophilus NCFM] lba0747 laci042

MKLIYQNMLGFLLIIVTTISIIGYSEIGYARDQAYMQNYQRMEGYANSLGNLAAAEGKDDTAILSNSFLN

QLEFILRGDDVHLRIFNEKNEQIYPKTREKIQLSKNIFATLKNGQEIRIQNNHNENAPIASTKDAYTGVL

VPWMNGKNLIGVAWISSRVKHVERPIYMAKRNLLRALLTTVAVGLILSFIISYYSTKRIKRLSRATQKVA

SGNFNVQIKHKDSDEIDQLAENFNQMVLALKRSNEEVKAQENRRDQFMADAAHEMRTPLTTINGILEGLQ

YDAIPEESKPKSIALMQRETKRLIRLVNENLDYEKIRNNQINLIKTNFNATPVLLDLKSQLMQNAKKAGD

KLIFEVPTDLPIYANRDRFTQVMVNLVQNAIQFTHDGKIKVSGKRLKHGAEFSVKDNGIGMSDDQIKYIF

ERFFKADPSRARMGTGESGLGLAIVSSLIKQHGGKITVDSKPGQGATFTVTFYDKGYEQFIEK

>gi|28379052|ref|NP_785944.1| histidine protein kinase; sensor protein [Lactobacillus plantarum WCFS1] lp_2505 lpla042

MKLIYQQMLAFFAVIITVIVVLGFSFIRTTRTMLYQNSWQQLQQYAASIEKEAIRYNTKTNQVVGFNQQF

LNDGTVILRDQHIHFSVYDANKKLVYPSGMGTTMSGTPGGSIYDPKISAKDWKKLKRNKIITQSPHVTFR

TRNRDQPVNTPSLRTIDVLVPLFYKNGATKKFVGAISIGSFEQTLQANEQQIEKNLFIALLVSGIAALLL

SYILARYSVRRINRLRYATHSVANGDFDIQVDSNHKDEIDDLADDFNGMVNSLRTSNEEIKRQEKRRREF

MADAAHEMRTPLTTINGILEGLAYDAIPEEDKGHSIELMQNETKRLIRLVNENLDYEKIRTGQIALNKTN

FDGAEVLHNLVEQLTKKAAAADDEFKLTVPEKLPVWADYDRFVQVLFNIMQNAIQFSQANVIEVTGSRTD

HATVVAIQDHGIGMTPDQVKNIWERYYKADPSRKNTKYGESGLGLAISHQLVQQHGGSIKVDSVENEGTC

FTVTFPDEGFDKPIIDD

>gi|116333922|ref|YP_795449.1| Signal transduction histidine kinase [Lactobacillus brevis ATCC 367] lvis_1315 lvis042

MKLMYQQMLGFFAAITIILVLMGVSYSQMTRHMVYSNTWNSLEKYSNSLIEQSLRISSQSPKTVNFDTMA

LENSEQLLQNQAISTTIYSSSNRIVFPANIYQQAINQTDWKKLKNNQIIHKVVDRRVRNKNGRVSPAVIE

VMKPYFYNHKLVAVVVMGAFVSDINTNVNQINRNLIRSLLVSCLVAIVASYILARYYTSRINRLRKATNQ

VAKGNYDVQMASRNRDEIDDLINDFNGMTHSLKASQEEIQRQEERRREFMANASHEMRTPLTTINGLLEG

LAYDAIPEESKEESIELMRSETSRLIRLVNENLDYEKIRSNQITLNLHEFNAVDALHNIVEQLKQKADDS

GNVLELQAPKEIEVYADYDRFVQVMFNITQNAIQFTQNGTITLSAQRGYEETIVKVADTGMGMSDEQLKN

IWERYYKADPSRKNTKYGESGLGLSIVHQLVQLHHGKISVTSKEGVGTTFTVIFPDQHQTHSQRAKTNE

>gi|81428826|ref|YP_395826.1| Two-component system, sensor histidine kinase [Lactobacillus sakei subsp. sakei 23K] lsa1214 hpk48 lsak042

MKLIYQWMLAFFGVIMTTIVIVGIAFTQYSTKTAYNNTWDQLEGYASVIEREAFKQGSQATLSKQFIDDS

QAILSQQRVSFNFFNAQNEMVYPAPNSNVSVKKKYWKYLKKGQVQRLSMSKDSQQFNNDQDQLVVFKPVF

YNDKLIYVIGVGSSIKNVKSGLVTMRKNLLIAFLLSTIGGILLSYVLARYQIYRISRLRNATHLVAEGDF

DVHIDNPGKDELDDLATDFNEMVHSLNESEAEIKRQEDRRRQFMADVAHEMRTPLTTVKGLLEGLAYDAI

PEEMREKSISLMQNETNRLIRLVNENLDYEKIRTNQIKLNQQTFNAVEALQNIQEQLQKKAADAGDTIVL

EAQEEVAIYADYDRFVQVMFNIMQNAIQFTNNGQITVTAEAGFHETILTVSDTGIGMTSEQVKNIWERYY

KADPSRKNTKYGESGLGLAIVHQLVQLHHGTIEVQSEPDQGTTFILTFPDETVTTDAN

>gi|16077444|ref|NP_388258.1| hypothetical protein BSU03760 [Bacillus subtilis subsp. subtilis str. 168] BSU03760 yclK bsub042

MMKIKYLYQLLLSHISILILAFVIIISLFSHFVKEFAYQNKVEELTSYAVQIANEFQSGQVDMRRLYPYQ

DILSTRKTQFIIFNEEQQPYFLPEGFHHPPREQLKKSEWNKLKKGQTVTIRADGRFDDEVSLVAQPIFVQ

NEFKGAVLLISPISGVEQMVNQVNLYMFYAVISTLVITILVSWLLSKFHVKRIQKLREATDKVASGDYDI

HLENSYGDEIGVLASDFNIMAKKLKQSRDEIERLEKRRRQFIADVSHELKTPLTTINGLVEGLNSHTIPE

DKKDKCFSLISEETKRMLRLVKENLDYEKIRSQQITLNKLDVPLIEVFEIVKEHLQQQAEEKQNKLMIQV

EDHVIVHADYDRFIQILVNITKNSIQFTQNGDIWLRGMEGYKETIIEIEDTGIGIPKEDIEHIWERFYKA

DISRTNTAYGEYGLGLSIVRQLVEMHQGTVEIKSEEGKGTKFIIRLPLTAKQQ

>gi|16803548|ref|NP_465033.1| hypothetical protein lmo1508 [Listeria monocytogenes EGD-e] lmo1508 lmon042

MKQMKFKYAYQLFFTQFIILLIACIMIGVLVSHSLKDYFYQSQVDDLTSYGQTISRDIRYSPQDATMQVL

NTYQRILDVKKIHYTIKNANDETIYPTQMNQPLPKDFSISADDKKKLESGETVSKKIDNRFNKEMTIVYV

PIMNGDKFVGSIVLNSPISGTEQVIGTINRYMFYTILLSITVALILSAILSKLQVNRINKLRAATKDVIQ

GNYNARLKENNFDEIGALAIDFNKMTQTLETSQEEIERQEKRRRQFIADVSHEMRTPLTTISGLTEGLVN

DIIPKSETDRCIALIDTEARRLTKLVNENLDYEKIRSNKIKLQKTRFNGKEFLELIKEQLDYVASEKGNT

ITVAIDKDMAIYADYDRLTQVFINIVKNSVQFTENGQITLTGTQDYKESVLTITDTGIGMNTEELEQIWE

RFYKADMSRTNTAFGESGIGLSIVKQLIEYHDGSITVTSEPNKGTSFTIRLPFFQDNEQ

>gi|20808918|ref|NP_624089.1| Sensory transduction histidine kinase [Thermoanaerobacter tengcongensis MB4] TTE2569 tten042

MKKNRLFKKLFISNIIIVAITLAVVSIMFYIMFQNYYFAEKEKIMLEEAQEINAILNEFAVGDINLDKWN

QELNVVTRLINATVWIVDKEGLVYSQSQQGKPWMKITLSKADIQNILKGESIVKKGYFGGAFTSPVLTVG

VPLVIKGHIEGAIFMHASLEEMNRTVLNIFLLMLLSGGMAFVVSFILISYTSNRISEPLKKMSLAAREIA

KGNFSARVDHFEEDEIGDLARSFNVMARELAQLEDMRKEFVANVSHELRSPITSIQGYIDGILDGTIPKE

KTYDYLKVVQEETRRMSRLINDLLEMTKLESGEFPLNRTEFDINELIRICIIKFEKRIVEKDLTVKVDFE

EDRRFVFADRDKIEQVLTNLIDNAIKFSKPKGIIHLFTEVKGDKVFITVKDNGIGISPEDLEHIWERFYK

ADKSRGKDGAGLGLYIVKKIIEAHKEEIWAESELKKGTSFTFTLPIKKV

>gi|148381482|ref|YP_001256023.1| two-component sensor kinase [Clostridium botulinum A str. ATCC 3502] CBO3542 cbot042

MKKGLFAKLVATFTIIISVSFIIIATFLSYWFESYYFDQRKNQLFTESQFVGNAAVRYIEGSISSERINE

VINYISNYLSVNIWITDKYGYVYAVSNPKHKNLVGVQVLTDELEELRRGNNLENKGQYKSAFSTPVHTFE

VPIFYKGVFSGAIIMHTSIDEIKDPLKKVYNIIWISAIFAIIISCIVIYYFSQRIIIRPLAQINYVADKI

SKGEVGKRVDIESDDEIGALASSFNSMAESIEQVESNRRLFISNVSHEIRSPITSIKGFIGGILDGIVPE

EKQNYYLKLTYEEIQRLTRLINDLLDLSAIEEGHLKLNLEEIDLNEIIRASVIKFETKIRDKKLKVNVSL

EDDKVYVVADKDRLTQVVINVLDNAIKYANEGGNVKLNTKIKGSKVIVSIFNDGPVISDEDIKHIWDRFY

KADKARSSKVSTGLGLSIVRSILTQHGEDIWVNNSPEGGVTFNFTIKRA

>gi|148543653|ref|YP_001271023.1| integral membrane sensor signal transduction histidine kinase [Lactobacillus reuteri F275] LREU_0417 lreu042

MKLMYRLMLSFFAIILTLMVIVSISFINVTNNTMYHNTWQQLKSYSDSLIQDSIRYNMATQSFEGFATES

LNSNANLLTRQNVHFAIYDTTHRKIFASNGFAPSMSKSDWKKLKKGETVCTKLITPKINSRVSKGASPRM

TEVSRPYFYKGKMIAVVSIATFVSTIEQNMHQIKINLIMALLTASLVTLAVSYFLARSITKRIDRLRMAT

HQIAQGNYNVEVDDNGRDEVADLGRSFNQMTASLRESQQEIRRQEERRRQFMANAAHEMRTPLTTINGIL

EGLQYDAIPEEDKKHSIQLMQNDTRRLIRLVNDNLDYEKIRTNQIAMERKVFDASAVLENLREQLAKKAK

EKKDTLHLDVEKNLRVYADYDRFVQIMFNIIQNAIQFTDNGIIDIRGKRVEKGSQFEVQDNGIGMTPEQL

ENIWERYYKADRSRMNTKYGESGLGLAIVRQLVLLHGGKIDVKSQYGKGTTFTIFFPDREYAPHNIQPTN

NKDKK

>gi|184154874|ref|YP_001843214.1| two-component histidine kinase [Lactobacillus fermentum IFO 3956] laf_0398 lfer042

MRLMYRQMLGFVAVILSLMAILTVSFISVTDQTIYQSTWRQLTKYSDSLVWDSIRYNPKTNDFAGFENSA

LSSNARLLSRQDVHFAIYNAAKKELYASNGFTPKLTASDWKHLKNGDIVKKKADYPAFRRVNVEGKESYK

TSSEMTLIIHPYFYKQKLVAIVTIGTFVSTIRQNVKQLLKNFVFAFLLALLVALVISYMIARSLTKRIDK

LNTATHLVAQGNYQVHLDNKNKDELDELSANFNQMVGSLRTSREEIRRQEERRKQFMADAAHEMRTPLTT

INGLLEGLAYDAIPEEDKMHSIQLMQNDTKRLIRLVNDNLNYEKIRTNQISMERKVFDAAAVLENLREQL

RKKAAANKDELKLVVPDSIPVYADYDRFVQIMFNIIQNAIQFTTGGQITISAQRVAQGTEVKVADTGIGM

TEEQVQNIWERFYKADRSRMNSQYGESGLGMAIVRQLVELHGGKIDVDSTYGQGTTFTVFFPDREYAPHN

GSKHDHYDDA

>gi|116491733|ref|YP_811277.1| Signal transduction histidine kinase [Oenococcus oeni PSU-1] OEOE_1772 oeoe772

MRNYILTLLLFSIIGGIPGILYASNAQNWQDVLISGYTGWYLLYWVIFSAIFSAFAAFRRYRDFDKPIKQ

LSQGANQVANGDFSVYLPAIHDAEHYDYLDILFEDFNLMVSELGSTETLKSNFISNVSHEFKAPLAVIQG

YANSLNDDSLSVKERQKYLRAIVEATNNLASLVTNILRLNKLENQNMPTKLSYFDLSRQLADCALQLEPN

WEKKEIDFQADLLDHCPILADEDMLAIVWNNLLNNAIKFTPEKGKVSLIQKKQDQGVLISVVDSGIGMES

SVIDHVFEQFYQGETSHSNSGNGLGLAMAKKIISLSNGTISVQSKVNLGMTFSVWLPTDQKNDPD

>gi|116618065|ref|YP_818436.1| Signal transduction histidine kinase [Leuconostoc mesenteroides subsp. mesenteroides ATCC 8293] LEUM_0957 leuc772

MKKIKKHKITFQKVSIKELIAAYVIFLLFATIPNIVLMHGGQLALFTNSNFIWYLIYWFIVTLIFIGLIS

YQKHVLFDQPVSQLNEATRQVAQGDFSVYLEPGHAREKYNDLDYVFENFNLMIEELGSTETLKNDFIANV

SHEFKAPLAVIRSYAEALKNETDEEQRDMYTQIIVDETDKMATMVTNVLKLSKIENQALQPHIKKYNVSR

QLVETLLNFEKIWASHNISLDINIPDDIEIAADPEMMELVWQNLLSNAFKFVPSDGQVTLRLQQNNEGTE

ITVRDNGEGMDHNTLNRIFDKFYQGETSHTNQGNGLGLSLVNRIVTLMQGSISVESKLGKGSAFTVWLPL

HHLNN

>gi|153953198|ref|YP_001393963.1| Two component sensor kinase [Clostridium kluyveri DSM 555] CKL_0561 cklu772

MKIQYKEPRDKRVNAKHSFGKEYLVTLGTVLLCCSGAVLINDAYMAEQANPWSMVLGISSYLAMICFIVS

GVIRWIKYTAYTKPMGKIGHAARKVAVGDFTVRIASERKDGKKDEMELLIDDFNKMVEELATIETLKGDF

ISNISHEIKTPLSVIQSYATVLHNGNISEERRQEYLETILESTRKLSALITNILRLNKLENQEIIHGETY

SLDEQLRCCILALEEKFEEKSIEFDANLEEVVITTDESLLEIVWNNLLTNAIKFTELGGSVMAELKVENG

IAIVSISDSGCGMDEETIRRIFDRFYQGDTSHSREGYGLGLALVKRIIALVDGNITVKSKLGNGTIFMVT

IKL

>gi|158320238|ref|YP_001512745.1| integral membrane sensor signal transduction histidine kinase [Alkaliphilus oremlandii OhILAs] Clos_1203 aorem772

MKSIKTKLILFFILLMFLSASLSSFLYTFIPFNNIRDEFKLNQQAIAISILELRQKTDLPLADIIKMASN

FMHDVSVIEDIHKIQISEEELEKVQNSEIVFLSSKKIPVSTTVVKVEEDYMQISLQPHNTMVQIVFSRIG

FTLVLYVGIGACLIALFSKKAVENIVKLTSAAREVAKGNFDVQIENNSKDEIGQLTKNFNTMVNELKGIE

YLRKDFINSVSHEFKTPIASIQGFAKLLQSEGLSEEERQEYARIIVEETSRLSRLSSNILRLSKLENQDI

FAEKKKFDLDEQIRKSILLLEPEWNKKNIEFCIELDKIAFVGDEELLQQVWINLIGNAIKFSETNGNITV

KLQQLGSFAKVKIRDEGMGMSEDVQTRIFEKFYQGDKAHSIYGNGLGLSIVKKIVDICGGRIFVKSASGK

GTTFIVELPLEKEEIKK

>gi|30020096|ref|NP_831727.1| Two component system histidine kinase [Bacillus cereus ATCC 14579] BC1957 bcer772

MKMLKVFGAVLALFSILTIIWSIAFYVATSLLNAFDVNVSPFVAFLISDMVGFVFIILIWTLIGILMRPK

REAMIWTIIEPIQKIAKGDFSVKIRNEEKYDGEIGVLVKSINDMTDELNTMEKMRQEFVSNVSHEIQSPL

TSIKGFARALQDDNLSEEKRKHYLTIIETETTRLSKLSQNLLKLTLLESEEYTPERVSYRLDQQLKQIVL

NSEPLWAEKEIELELDLEKVYVIADQESMSQVWINLIHNSIKFTPSGGTITIQLKEHETVVEVRIRDSGI

GISEEQKQHIFERFYKADSSRNRAYGGSGLGLAIVKKVLDLHHGEIKVESEEGKGTEFIVRMPNHEEK

>gi|22536309|ref|NP_687160.1| sensor histidine kinase [Streptococcus agalactiae 2603V/R] SAG0124 saga772

MTKLRRFRFPLRFYFTLMFVLTMLFSVLASLLLVAAIVFTFFQGVLTTHVLQVSALAVVFLSLVIASISM

WYGSYHLTKPILDISHIVSNVADGDFEGHIYRNSNRRKSYEYYNELDELSESINQMIVSLSHMDHMRKDF

ITNVSHELKTPIAAVANIVELLQDPELDEETQSELLGLVKTESLRLTRLCDTMLQMSRVDNQETIGELSS

VRVDEQIRQAMISLTERWQAKRINFQLDSKPYTVYSNSDLLMQVWINLLDNAIKYSEDIVDLSVRMEETN

NHYLRVIISDKGRGISQYDVQHIFDKFYQADQSHNQQGNGLGLAIVKRIIVLCKGRISVSSQLEIGTEFC

VELPLS

>gi|116494702|ref|YP_806436.1| Signal transduction histidine kinase [Lactobacillus casei ATCC 334] LSEI_1208 lsei208

MLGIIIGLSVICLVLAITLLLIIIDIRRINHELTYINHIETNAGVTTNTNFPLVRKLAAGINDNLNATRQ

LRLEQIAQEKKIHQMLLNLTHDIKTPLTVATGYVQLLNRDPHADAKQSLARVAHNLRSVNYYLHYLMDFN

LIQEKSTALKLKPINLSKLLETELFDYFDQLTANGMQVTPKIAPNLVLTTDETMMRRVFQNLIGNWLKYA

KNQASVTLVRQDEQHLTITFTNNTDKPLANADQLVDRFYTTDSARTTQSVGLGLSIVQSLTTTLGGKMTL

QADRQQFAVTLTFRDHNV

>gi|125717270|ref|YP_001034403.1| Histidine kinase, putative [Streptococcus sanguinis SK36] SSA_0402 ssan208

MLVLVILFALTSLSLAIGFIRYHLAVKDVAQQIRRKRKTGSQNRLAPAAHAPSILELTEEAELLFGELDK

TTFVVQQEKKTLDMAISNIAHDIRTPLTIASGYTQQLLKDDSQESQQLVKIADSLGMVSKRLEALMEYRR

LMEGAIRANVQPTDISQLVTQQLFTYYDAFQKAQIDLQVELAEGLELETDPEILERIVQNMISNVLKHGR

KAASISLKKDGEHCIFTVKNIVQQPIQYLDKLTNRFYSENLSSSEESSGLGLYITQQLVEILGGDLTMKA

EDDWFELVVTL

>gi|24379475|ref|NP_721430.1| putative histidine kinase [Streptococcus mutans UA159] SMU.1037c smut208

MIVILFFGLSLAFILAIIYLIRYHLAVKSLSQQIEEKINNTSQRRFTLKNQPKSLIELTNKIENLFEKIE

KTSLIALQEKKTLDMAISNIAHDIRTPLTIASGYTQKSLHKETVDAADLEKISENLTIVSKRLEALLEYR

RLTEGTIRPQVQKIALSHLVTKSILSYYDMFQQVGITLDLQIEEKIPFETDPEIFERFFQNIISNILKHG

KNQAQLILKKTDQKIVLQTKNIVRQPIQHLDQLTTRFYTENMSDTEKSSGLGLYITQHLVDILGGELILT

TEGNWFILTVTL

>gi|28209930|ref|NP_780874.1| sensory transduction protein kinase [Clostridium tetani E88] CTC00159 ctet208a

MTFMQGLIIVFLIIITVVFATLYILLHKELKNITKQLKEINNTKTNSKILISFSNNLVKNLALEINKSLE

KKSATEAEHKRMDLEIRQSISNISHDLRTPLTSIIGYIQLMEDDSLSQNERSQYIDIIKKRAKSLQTLIC

SFYDLSRLEGREYKFDLRALNLYNILCDLIAVSYNDFISNEIEPIIDIDEQIPMIIADENAVRRIISNLI

QNMIKYSNEFISISLKYNKDHIILNFTNDAPNLKEDDVKYLFQRFFTADRTRSGESTGLGLAIAKELVEQ

MGHEIFAKLSKDKLSIIIKWKVINDSYIK

>gi|15613382|ref|NP_241685.1| two-component sensor histidine kinase [Bacillus halodurans C-125] BH0819 bhal208

MTLLLSITLVLLLFICLMQSKQNLERNKQLAYVTHKLDEIRTSQSAETVMVFTGDEQLRELLRAINDLIE

EHNQVTATYQRTELSMRKMLANISHDLKTPLTVIHGYVETILAGKGSANDRERLEKVQTKTIEIIELINK

FFDLAKLEAGDQNLTLKKVAVNELCKNRILSYFDLIQNEKLQVEIEIPSQPFYVLADEKALDRILDNLIS

NAIRYGKDGKMVGLRLYHDDAYVSIEVWDRGKGISDKDREKVFERLYTLEDSRNKQYQGSGLGLTITKRL

TERLNGHIHLESRPYDRTSFIVKLKRITG

>gi|28211447|ref|NP_782391.1| sensory transduction protein kinase [Clostridium tetani E88] CTC01804 ctet208b

MNYVILIDIIFILVAVIINLNKKIHNISQAIKNIEQGNYNERIRMQNSNKNLNNLIESINGLVDKFQNSM

KLNKEYEDERRNMITNISHDLRTPLTSLLGYIEFIKDNKNLSEEEREEYINIIEEKGNNLRNLMSEFFQL

SKLEGNDIKLNIKKINLSEIIRQSIILFYNDFNNKEIEPVIDLPSKEIYVMADKIAVERVLNNLISNAIK

YGYEGKNIGINLEYNKKNVQVTVWDQGKGIPEEELKLIFERLYTLEKSRNKNFQGSGLGLTIVKKLIEMQ

KGTITVKSIPYKRNEFIFTLPSL

>gi|116495221|ref|YP_806955.1| Signal transduction histidine kinase [Lactobacillus casei ATCC 334] LSEI_1741 lsei741

MRFRDYLKDHLSPIGGYILCVGLAALIIWLDPQHRVKAMTLWYAVLLVTLLASAFLIARYWRVRRFAARL

QERIDAQEAALDWPLPGGESATEQLVTKTYNQILTQHRQQVTALLAQQQDQKDFIDSWVHEIKVPLAAVT

LLAESFEGQVPDDKLDNLNLQLDQIEFYVEQVLYYSRLDSFSKDYLLHETSLRPLINDVVANQRNGFINK

RLSFRLNGTDQIVLTDNKWLRFILAQLISNAVKYTPEGGRITALIRNTGKETQLQITDTGIGIPKDEQHR

VFEKGFTGSNGRLSNHKSTGLGLFLAEKLAEKLGHHLSLTSVINQGTTVTIHFPYVSYYGEADQTVHHPE

SHAIAVQTQTRSHPDS

>gi|116494905|ref|YP_806639.1| Signal transduction histidine kinase [Lactobacillus casei ATCC 334] LSEI_1419 lsei419

MMKAYCRSRWAIWLLLATLLATGFLASWLLANSFEIVLNMWFFATAPLAVIVSIDYWRFRREWQQLQDDH

PIDPVQITDPLSLAYYQKIQTLTQKLRTTRDTAHEQEQETLDTLQLWTHQIKTPLTALDLLLQVEPVNAK

DARLEISKIDRYLAVMLNYLKLTTLNTDLVLTELPLKPLVNETVRDLAKLFIAKDLTVTVGPLPTVISDS

QWLHFIFEQLLTNAIKYTPHGSIKIYAKGDAVMVADTGIGILAEDLPRIFEQGYSGYNGRANQKASGLGL

FLSKQIAEKLGLTLTVTSEIGQGSTFAIHFPQTRWQVE

>gi|81429064|ref|YP_396064.1| Two-component system, sensor histidine kinase [Lactobacillus sakei subsp. sakei 23K] lsa1455 hpk1 lsak741

MTFKKFIRDHLFHIVFFFGGMFVLDIVLWLDPHMRLAKETLMYLDFLLTIFFCAFLIGLYLYHRKWFRTI

QIRLNAKEDALNWPLTGATSAEKQYFQKYVNSLLDYHQQSIERLMHAQQDQKDFIDGWVHESKVPLAATQ

LLVESIEDQIPEEKFNQLTDELVQIEHYVEQVLYYSRLDSFSKDYLVQEYALKPLINQTIRQNRNYFIQN

RIQFKLTGEEQTVLTDAKWLVFILNQIVSNALKYTPQNGQITIDLAHDEQGVWLSVSDSGIGIPAEDLPR

VFDKGFTGQNGRQSNQRSTGLGLYLAKSLSNKLGHATLCQLNARKWCDFQITLYLFKLL

>gi|148544994|ref|YP_001272364.1| integral membrane sensor signal transduction histidine kinase [Lactobacillus reuteri F275] LREU_1788 lreu741

MNKRYFKLFWRSILFPTIGYLFLLGLIGLLVILYNLPTVFWGDVIRFSLPFFIVWLIGSGAIKYYRLRHL

NISQLDKFTPTNMTEGKLVELLQDEQAKNIDTIRQLQATQHEQLDHLELFTHEIKNYLTSLNAAAENSPT

VASPEVKKNIHQANYYLNLLLNDERLAINNHDYDFQWIDIANLINNILQQNSAAFIHKQLIPELTGLDGI

KVLTDRKWLLFCIEQLLSNAIKYSAPNSTINISWEMNSLRIADHGCGISMNDLPRIFDNGFTGKNGHQTT

AATGMGLYLVKKVTEHLNFKVSISSVQSKGTTAILSFLPDNIRSSN

>gi|116617945|ref|YP_818316.1| Signal transduction histidine kinase [Leuconostoc mesenteroides subsp. mesenteroides ATCC 8293] LEUM_0835 leum741

MIKKIFGLALQFLSDFWYLYASFMLTFLLLMWSVIMRSLSLDSMFDVLWGGCYILIVITCILFWKWYRAH

KGLIQLLCEEEVHTLSSIATLSSHERMYQQLIKKQQLHQEKMQAQLNEDANDIQDYYAMWAHQIKVPLSV

LDLMNQTGTIEKYETSNQLLVINQYLDMMLQFIRLKNFNQDIAYQDMSVQKSLRSVIKDYKYWFIHKDLA

VSINEFDFSVVSDAKWLRFVFEQVIFNAIKYTPQGKINIICQNDNQVIIKDTGIGIAQNDLPMIFNQGYT

GFNGRINSNASGLGLYMVKRILNNLGHDITIQSEVNVGTTVIINFQQTVIK

>gi|170016953|ref|YP_001727872.1| Signal transduction histidine kinase [Leuconostoc citreum KM20] lck_00595 lcit741

MSTVKLYAKISISEWPIILAYSLLMLLLSFLGYLYKIDLVIIIDLIRYSIIPLLIIVSTRIYQKIIDTKQ

VQKAIIEQKATNIKMHGFYGHLYAKTLTTFMNKAQADNRQLIEAFKERQDYLTLWSHEMKTPLTTLTLAA

ENNTVVPSDLVSEKADLLTYHIEQLLTIDRLADFNKDLYFEYISLSTCVTDVVKQNTSYFLSHKVVPIIT

LPEVMVLSDQKWLCVMLTQLISNAIKYSPQNSRIALTFDDHTLWIKDFGVGISQSDLPRIFDQGFTGTNG

RLNNEATGMGLYIVQRIAAILDIQVTIQSHQQKGTSVAVQFNPKKLKYNKL

>gi|16803781|ref|NP_465266.1| hypothetical protein lmo1741 [Listeria monocytogenes EGD-e] lmo1741 lmon741

MNWWSYLKDKRFFLLFFCSIMFFVGVLISIDPNSKLTLGNFIYLYVFVLVFLLSYLVLGYFFKYSYWREM

KELVSGEIEENIIELLPKPRTREQAFFNQLMAKKHKEELRTISKLQDKQQEYHDFILYWVHEVKTPVVAS

KMLINNPDLNDTETIFKQIDEELTTIDKLVMQALYFSRLDTFSKDYFIQEQNLGVVVRESVKRHSKLFIG

KKMKLDLQNVDMDVRTDSKWLGFILDQILSNALKYTKSGGEVKIWCDTEASGKKVLHMKDNGRGIKEEDL

PRVFEQGFTGNIGRQEKKATGMGLYLAKQMAKKLGHEICIQSESGVGTEVKIYFEQKDDYLLIAKD

>gi|146295564|ref|YP_001179335.1| integral membrane sensor signal transduction histidine kinase [Caldicellulosiruptor saccharolyticus DSM 8903] Csac_0508 csac741

MKDYINIIGAYLKRNMLLIIYLLVSSCIFFSISFLYSLPLEPTVYSLVLTYIFAFIIGITDFFSFYKQHI

TLEKLKRNITIADFSFPIAKDLIEKDYQELIKIINESKIEILTNNEKVYRDMIDYYTAWAHQIKTPIAAI

KLVLQAEQSKISSELLEQLFKVEQYVEMVLQYLRMENMSNDLLLKKYSLDHIVKQALRKYSLIFIRKKIK

LNYKELNCHVLTDEKWLTFVIEQILSNALKYTNPGGQISIYMENNLPNTLVIEDTGIGIAKEDLPRVFEK

GFTGYNGRLDKKSTGIGLYLCKRILDKLSHKIIIESEVGKGTKVKINFDTVDIAPD

>gi|125718485|ref|YP_001035618.1| Histidine kinase, putative [Streptococcus sanguinis SK36] SSA_1684 ssan741

MDTQDKFFFLKSYLYSRRFFLALLILLLGFILIFAFVFDAYRSLLEYVALLLAFLSFLFIGADAWTSYKA

YRSQKLQASAQAQTPLEKLLQERVEELEYEQKNQLLVEQEKYNDLLDYYTLWVHQVKTPIAASSLLIGDL

KDKEAKSQLEQELFKIESYVHLVLQYLRLESFHDDLVLKQENLADLVREVVKKYALFFIQQGLSLNLHDL

DHTIVTDKKWFLVILEQVLSNSLKYTKEGSIEIYFHEGSLYIKDTGLGIQNADLLRVFERGFSGYNGRLT

QQSSGLGLYLSKKIADQLGHKIAIDSQVGQGTTVSIAFPEKKLIFE

>gi|55823250|ref|YP_141691.1| sensor histidine kinase (homolog to HK01Spn & Sth) [Streptococcus thermophilus CNRZ1066] str1335 sthe741

MMDKRQFVKYQIRSRLPFYLLFIFNEIILVLFAFLFEEAADILHYALTILTFVAIIIAIWDLVSSYQSYN

LAYLYEKMTPSSPLEFYLAQALENAKESQMVQTEEERKRYTDLMDYYTLWVHQIKTPIAASQLLLGDVTD

SKTRALLEQEVFKIDAYTNLVLQYLRLENFHDDLQLRQVALDPLVKEVVKKHSIFFIQKGLTINLHDLDV

EVVTDEKWLLVIVEQVLSNSLKYTKSGGIDIYFKDNRLYLKDSGIGIKDSDILRVFERGFSGYNGRLTQQ

SSGLGLYLSKKIADQLGHDISISSQVGQGTTVSIHFQKQKLAID

>gi|16080091|ref|NP_390917.1| hypothetical protein BSU30390 [Bacillus subtilis subsp. subtilis str. 168] ytsB BSU30390 bsub741

MIKAFLIERRSWIAAFLFQQALMLFIAFVDPSISFGNVLYMVYLCILFFIIFLWFRYRKETAFYKSLKTW

ENNLDVTAINEPETPFEAMVERSIAGQTEHLKQTAARHRLALENEKDELMAWIHEVKTPLTAMHLIIDRM

EEKALKSQLSYEWLRIHLLLDQQLHQKRISFIENDLSVEFIQLQPLIFKEIKDLQSWCIQKGIGFDIQLE

AKEVLSDAKWLAFIIRQLLTNAVKYSEASEIEIKSFQKGEQTQLQVKDCGRGIDPKDVPRIFDKGFTSTT

DHHDQASTGMGLYLAKKAAAPLLIHIDVESEFGAGTVFTLTFPIRNQFEHVISV

>gi|116495159|ref|YP_806893.1| Signal transduction histidine kinase [Lactobacillus casei ATCC 334] lsei_1678 Error en el sitio de inicio: la secuencia correcta es (sitio de inicio en 1676428 de NC_008526 lsei678

MIPKKQTLEQTRKSPRISLKVKWALTVGVGIFVTFVTFSMILYGAMRQILVNQERRTVTD

TLNTVVQRLSPVSGDLTMAQVVPRLEGAMPSGQASIPAPSESQNRIFSDSVIQKLAQADV

SVSVFSPDRTNIFQSRDTPITLGKVHGVEVQEANIDDFHGLVGTAPIYSATSSDLIGYVQ

VTDKLTAFHATMHEITLLIMGLSFAAILISILIGYALATRFLKPIKLITNAIDVVNEEPQ

STVRIPALKRNDELGDLVLEFNGMLDRIQRYIDQQGEFVQDVSHELRTPVAILEGHLQLL

NRWGKDDPEVLAESLTASLQEVTRMKSLIQEMLDLTRADQVDIQFPNAVVDVHQTVQQVV

GDFRMIHPEFTFTLDDDLEQKTFVRMYRNHLEQVMIILLDNAVKYSTDRKEIHVSMAENS

VSGVAVAVQDFGEGIAEADRRRVFNRFYRVDKARSREKGGNGLGLSIAQQLVESYHGQIE

VDSALGHGSIFRIDLPTITEAKAQALAAQETTKRAEAGKTDTHIKSNL

>gi|81428993|ref|YP_395993.1| Two-component system, sensor histidine kinase [Lactobacillus sakei subsp. sakei 23K] lsa1383 lsak678

MITNDLETPSENNNKGRKISLKVKWAAAVGLAIFITFAIFSIVIFSSISNILLHKEVDNVHDTVTVIQQR

LGRPEKTLTSEFVAEQLDPAPRDSKAIYQDSVLMRLAQQDTTVKVYDQNRQGLFASRGRATSFPKQDTDV

LKREHIFGKIHLMGIRPIKTANTNRTIGYVEVTNNLRDYNHAMLELRIVLVVVVLFAMLFSAVIGYLLAN

RFLQPIEAMKQTIEAINVEPNSNVRIPETRRSQDELSDLVIAFNEMLNRIQHYFEQQDQFVSDVSHELRT

PVAIIEGHLQLLNRWGKDDPEVLSESLAASLQEILRMKNLIQEMLDLTRADQAVTQGTTEVTDVGKVVLQ

NYHNFEMLYPEFTFILDDDLHGQALVNINRNHLEQIMIILLDNAVKYSTTRKEIHIAVANDERYAQVAVQ

DFGEGISEEDKKKVFHRFYRVDKARSREKGGNGLGLAIAQQLVEGYDGNVSLESSVGHGSVFRIELPLIR

ETKAAPEEDAD

>gi|28378255|ref|NP_785147.1| histidine protein kinase; sensor protein [Lactobacillus plantarum WCFS1] lp_1545 lpla678

MTDADTNQVTPKPRRRWSLKWKWALGTAIGSALIFICFSLLVYKSFTNLLLRQEQRNVASAVTTVQQSLR

TESKGLTIKSVAAKLQPEASVEPATTMSERKHGVLQSKIFSDSDLTALSRTNLAVTVYDPHGNTLYLSRK

DAHEFSKNKTRQVVVIGRGSDAELVGRAPIYAASNHRLIGYTQVTDNLAEYHSTTQNLIWIFIVMTMITI

FGATLLGYFLAAFLLRPMRKIKQTINAVNDDPQTDSRVPDLKRNDELSDLAVVFNDMLDQMQRYINQQQQ

FVEDVSHELRTPVAIIQGHMELLNRWGKDDPQVLSESLAASLSETKRMQSLVQEMLDLSRAEQLEINFSH

ETSDVQKLVTQAFNDFTMIHPDFHFTFDDDVKKPVYAQIYRNHLEQILIILLDNAVKYSTRRKEIHLSLS

TDYRYIEVAVQDFGEGISKDNLDRVFNRFYRVDKARSRDKGGNGLGLSIAQRLVEGYHGHISVESVVGQG

SIFRFQLPILRDAKLLAKAEAETNDVEKIAKSELPAGIRPATRDEDEAPLDDHEHDH

>gi|116333654|ref|YP_795181.1| Signal transduction histidine kinase [Lactobacillus brevis ATCC 367] lvis_1017 lvis678

MTQTDKTPELTGDSVAPKKAGFRFSLKWEWALSTAFGVLVIFAAIVFLIFNSFMTVLMRQEKTHVSDTLN

VVVEKLNSQKQLTSTNVNQLLRPQAVVSATKQPTPYNNAVIASLARDSQAVTVYDADGQSLFATRQGDVS

FRRASKQSISTQTVSGRKNLVGRSPIRAKQGNRILGYVQVTDNLRDYRATQVKLLQVEVVLGLMAGLAAA

VLSYSLATFLLRPVEEIRETIHAVREDPQTDKRVPEMHHQDELSDLGSLFNDMLDTTQRYMDQQQQFVED

VSHELRTPVAIIQGHMEMLDRWGKEDPEVLDESIKASLQETKRMKSLVQEMLDLQRAEQIEVNYPNEVTD

VSEVVEQVFDNFKMIHPDYVFILDDDVHQPLQAQIYRNHLEQILIILMDNAVKYSQTRKEVHLTYESDLR

NLEVAVQDFGEGISQENRKRVFNRFYRVDKARSRTKGGNGLGLAIAKRLIEAYHGQITIESALGHGSVFR

ISLPILSPEAAQALNAKKEQTQAANDIPGIQSELLVDDSEDSTKSE

>gi|90961499|ref|YP_535415.1| Transmembrane histidine kinase [Lactobacillus salivarius UCC118] lsl_0523 lsal678

METTNEKKKKKFFSINLKWSLGTGLGVVIIFAVFATLLFQSFSSLLIRQEKGHSEEAMNAVTLRLAQSSN

TLTDTGVRDILSSSWNDDIQNRKQAAIYSDSVLTATSGKNIGISVYSVNKELLFASRGVLVDFSSKNKQK

ITKINGQTVYIVRRPVYSNKSNKLIGYVQVTNKLADYDATRHKLILIFIVFGVTAGLASALVGYVLSAWL

LRPIELINETIGKINTDDERDSLANVRVPILNQNDELSELSNLFNDMLDRMERYIQQQQQFVEDVSHELR

TPVAIIQGHLDLLSRWGKDDPQILEESISASLQEITRMKSLVQEMLDLSRAEQVELQFGNEVSDAREVGL

QVFNNFKMIHPDFTFVLDNDLKNNTPVKIYRNHLEQVLIILMDNAVKYSQDRKEVHMSISKNSKLVEFVV

QDFGVGISQENIDKIFDRFYRVDKARSRDKGGNGLGLSIARKLVEGYHGSMSVESVEGQGSLFRISLPLV

DLDNKK

>gi|116492483|ref|YP_804218.1| Signal transduction histidine kinase [Pediococcus pentosaceus ATCC 25745] pepe_0715 pepe678

MENKVIKKKRMSLKWKWAIGTGVGVLLIFALFSFFVFKIVTDSLYAEEHRDVESTVTQVRSRLQNANEIN

YKTTDIFRPDLNQKSAVNNNQGGFNDSLLASLTRRDLVVSVYNLEKESVFSNQRISMKTTLSERKSIKRS

KFHHKDIIVGQAPIINKDNQVIGYVRVVDLLVKYNNRYQRLIWLFTLTSLLIFMVISIFGYGLTAYLLKP

MNLINQSMRNLEEDPQTDLRVPEIDTNDELSDLAHEFNDMIDRMQRYIDQQRQFVEDVSHELRTPVAVVE

GHLKMLDRWGKDDPQVLDESIKASLEETQRMKSLVSEMLDLTRADQIEINYADEKTNVVQLVNQVFNDFK

MIHPEFNFVMDDDITGEPIVPIYRNHLEQILVILLDNAIKYSKDRKEIHISTANTKKTVEIAVQDFGEGI

AEENLKQVFNRFYRIDKARSRDQGGNGLGLSIAKKLTEEYHGNISLESSVGSGSIFRIKLPIIEDQQIEI

SEK

>gi|116630025|ref|YP_815197.1| Signal transduction histidine kinase [Lactobacillus gasseri ATCC 33323] lgas1397 lgas678

MKMMKHVKKQNKSTKETLKSSLTFKWVSLVAATITVSFVIFSIAIYSLVKQQIVSQERNLTESVATTFQR

RLVEIPTSLQISNVVPQLSPNTNRILEGQTPITSNDGGNVFNDDVLATLSNRDTSITIYNPSGDVVFSNG

NVPDNGMPHFSKTENHVLKVETTKGKIHMKIYQKIFSDKTRRLTGYLIVDNSMDQQNHVLKSIRNWMIGL

SIIAIIVFVGLSYLIVNSVVQPIKKMSQISHDINEDPTNKRRVPDLHRNDELGELAISFNEMLDRMQAYM

QQQKQFVGDVSHELRTPVAVIEGHLNLLERWGKDDPQVLEESIQASLQESKRMKHLIQEMLDLTRAEQVD

LQYPDKTADVNEVLNRTVNDMRMIHQDFTITYDDSDLAPDTIIKIYRNHLEQILIILIDNAIKYSTDRKE

ILVDAATEKDKVKISVQDFGEGIAPDEQDKIFNRFYRVDKARTREKGGNGLGLAIAQKLVESYHGKISVS

STLGSGSKFMIEFPLLKSKSENK

>gi|116514433|ref|YP_813339.1| Signal transduction histidine kinase [Lactobacillus delbrueckii subsp. bulgaricus ATCC BAA-365] LBUL_1388 lbul678

MPKQKAASGKKGTRHVSLIVRWVSIVAMTIMVSFLVFSAVVYNTVKQEALSQQKYMSNEVVNVLRKRLIG

IDDELEITNVVQQLSPNTRRILTGGPALSENDNSNSSVFGYSVLSTVTNPDMTVAIYNLRNEIVFHNGDS

APKLVKFTSESKSVTKEGKDGQSSLYVYRKVRNRKSHKLTGYIVVKNQMTGYNRLISRTLKAMLWLSILG

ISLAIVISYLVVRSVVRPINKMSKVAREVNADPNSTARIPELHRNDELEELALSFNMMLDRMQKYIEQQK

QFVSDVSHELRTPVAVIEGHLSMLKRWGKDDPQVLEESIDASISEAERMKHLIQEMLDLTRAEQISVHYP

NAIAEPMEVLTRVVGDIGMVHPDFKISLEVEDLDPDTKIQIYQGHLEQILIILIDNGIKYSTDRKEIAVA

AGQSGQEMQIVVQDYGEGISDEDKKKIFNRFYRVDKARTREKGGNGLGLSIAQKLVESYHGTIDVESVLG

QGSQFRITFPVLTDEEVKELESREEQKQAGTESPKQDPTI

>gi|42519506|ref|NP_965436.1| two-component system histidine kinase [Lactobacillus johnsonii NCC 533] lj1630 ljoh678

MMKHVKKQNKSKKETLKSSLTFKWVSLVAATITVSFVIFSIAIYSLVKQQIISQERNLTESVATTFQRRL

VEIPTSLQISNVVPQLSPNTNRILEGQTPITSNNGGNVFNDDVLATLSNRDTSITIYNPAGDVVFSNGNV

PDEGMPQFSKTESHVLKVVTTQGKIHMKVYQKIFSERTHRLTGYLIVDNSMDQQNQVLKSIRHWMISLSV

IAIVVFIGLSYLIVNSVVQPIKKMSQISHDINKDPTDKRRVPDLHRNDELGELAISFNKMLDRMQAYMQQ

QKQFVGDVSHELRTPVAVIEGHLNLLERWGKDDPQVLEESIQASLQESKRMKHLIQEMLDLTRAEQVDLQ

YPDKTTDVNEVLNRTVNDMRMIHQDFTITYDDADLKPDTIVKIYRNHLEQILIILIDNAIKYSTDRKEIL

VAAATEEDKVKILVQDFGEGIAPDEQDKIFNRFYRVDKARTREKGGNGLGLAIAQKLVESYHGKISVSST

LGSGSKFMIEFPLLKSESDK

>gi|148544451|ref|YP_001271821.1| integral membrane sensor signal transduction histidine kinase [Lactobacillus reuteri F275] lreu_1227 lreu678

MKQKDERRFVSLKIKWAAGTALGSLIIFCIVAMALFSAFTQNLFHQERQLLNQGMTNISTQLSTVDKPLT

KKNVSHLIDPDRNRSNIISGEEYKRPVIKELSDGHLVINIYNPDGKNILSTGRYIKKPQFTTNRNIHIAA

GPDHDVLVGQMPIYGQNNRQLIGYLQVENNLDAYMQSYHQLALVCILALCLVVIASGLLGYFLSLFLLRP

LDGIHDTVKEISADPTKDVRVPTTNRNDELAELITMFNEMLDRMQRYIDQQSQFVSDVSHELRTPVAIIQ

GHLEMLQRWGKDDPKVLDDSIKATLIETRRMKNLVQEMLDLSRAEQVEINFRNQHTIVNDVVHQVYNNFK

MLYPDFTFRLDDDLKEPIMVDIYRDHLEQVLVILCDNAVKYSTDDHKEIHIILSRGMNTVEIGIQDFGEG

ISPENVKRVFDRFYRVDKARSRKKGGNGLGLSIAKRLIEGYHGSITLESSVGAGSLFRIILPIIEDPETK

K

>gi|161507846|ref|YP_001577810.1| Two-component sensor [Lactobacillus helveticus DPC 4571] lhv_1587 lhel678

MKTMTKNKNKETKHSSLIVRWVSIVALTITVSFVIFSVVVYQIVSRQSLDQQEEMSLNVVTTLNRTLSSI

PDELEISNVIPSLSPATRRILKGEPAISDKDSTNNAFSDNLIASISNPDINSAVYNKHGEIVFTNGNVTP

KFKSFKEVSKNELVKRNGHQILLTYQKVYSSVNNKLTGYIVVSNNMTYYNSLMKNLLHSMVAISIIAIVV

FVVTSYILVFNVVKPIKRMSKVAQEVNADPNSGARIKELNRDDELENLATSINQMLDRMQSYIEQQKQFV

GDVSHELRTPVAVIEGHLNMLERWGKDDPQILDESIKASLQEADRMKHLIQEMLDLTRAEQIDVQYPHEV

TNVYDTVKRVVADLAMVHPDFEIRLDEDDLPQDTEIQIYHGHLEQLLVILIDNAIKYSTNLKQINVSAGL

TKKEVNIIVQDFGEGISKEDQKKIFNRFYRVDKARTREKGGNGLGLSIAHKLVDSYHGEISVESVEGQGS

QFKLTFPLISKKKAAKLKKLNENSELPKSNLE

>gi|58337789|ref|YP_194374.1| two-component sensor histidine kinase [Lactobacillus acidophilus NCFM] lba1524 laci678

MKMMIKNNKEAQEETKHSSLIIRWVSIVALTITVSFVIFSVVVYQIVSQQSLNQQEETSANVAVTLDRTL

SSIPNELEISNVIPSLSPSTRRVLKGGPAISSKDANNNAFSDNLISSISNPDISVAVYNKHNEVVFANGD

TTPKFKPFKGDSNVVKIKQNRRLLLITYQKVYSSVNNKLTGYIVVSNSMRYYNNLMNNLLRLMLVLSLIA

IVAFIGISYILVVSVVKPIKNMSKVAKEVNADPNSVARIKELNRDDELEELATSINKMLDRMQSYIEQQK

QFVGDVSHELRTPVAVIEGHLNMLERWGKDDPQILDESIKASLQEADRMKHLIQEMLDLTRAEQIDVQYP

YEVTNVNETVKRVVSDLAMVHSDFKIQLDEDDLPPDTEIQIYHGHLEQLLVILIDNGIKYSTDIKQINVS

AGVTKKEVNIIVQDFGEGISQEDQEKIFNRFYRVDKARTREKGGNGLGLSIAQKLVDSYDGEISVESVEG

QGSQFKLRFPRLTKKQAAKLRKLSENAKKPKSNLE

>gi|116618820|ref|YP_819191.1| Signal transduction histidine kinase [Leuconostoc mesenteroides subsp. mesenteroides ATCC 8293] LEUM_1731 leum678

MDNNEKETTKKAVRRFSLRWQLALGMASGFFVIFIVFALLLSGLIKHHYGALPNEVIFWLWVIIAFGGLA

IFIFALILTKVILRPVRLIETTIEAFEDDPMTEVRASEVGANDEFSDLTRILNRTIDRLQALISAQQQFV

SDVSHELRTPVTIVKGHMELLNRWGKDDPEVLEDSIKSSLAEVQRMETLINEMLNLTRAEQIPVDNVKEI

TDVGGTMHRVYDNFKMIHPDFSFTLDDDLSTKARVKIRQDHMEQILIILADNAVKYSLDRHEIHFALSQT

ANRVEIGVQDFGEGLKPEDAKRVFDRFYRVDKARSRAKGGTGLGLSIAQRLVEAYGGTIALESAEGSGSI

FTVYLPLYVEEKNESN

>gi|184155773|ref|YP_001844113.1| two-component sensor histidine kinase [Lactobacillus fermentum IFO 3956] laf_1297 lfer678

MVAMDERKRGNRFVSLKLKWALGTALGSLIISLAVVMVLFSSFTQDLLGQERQTLTQSLMAISQKLGQAS

ESELTASKVDQTLKDNALTPKEAGSGKVYRRPVVQGLSDGHLTVTVYNRSGKDLFATGEENQGFQKVSEQ

TIKTVSGKKHQYLIGRVPIRSSKTHQVIGYLQVENNLDNYFQHYRRLQLISILALSLVVIFSGLMGYFLS

YFLLRPLNDIQETVEVLSDDPTKNRRVPVTSRNDELGELAWMFNEMIDRTQRYIDQQSQFVGDVSHELRT

PVAIIQGHMQLLERWGKDDPKQLEESIQAALAETNRMNTLIKEMLDLTRAGQVEVNFRNATTPVKKLVNQ

VYDNFRMIHPDFHFSLDDDLDEDVLVPVYRDHLEQILIILCDNAVKYSNERKEIHLSLSRNLRAVEIGVQ

DFGEGISQEDLHRVFDRFYRVDKARSRKKGGNGLGLAIAKRLVEGYHGTVTVESQLGSGSLFRITLPIVE

PGDEKEAKNKESGK

>gi|170016760|ref|YP_001727679.1| Signal transduction histidine kinase [Leuconostoc citreum KM20] lck_00402 lcit678

MTETKKTTEKTLRRFTLSWQLALGMASGFFVIFIIFAFVLSQILKQYYGRLPETLYQWLGITILLGGLFV

FGFAFMLTRYILRPVKSIKTTIEAFEDDPMTNVRADRANVNDEFYDLVNILNRMIDRLQSLIGAQQQFVS

DVSHELRTPVTIVKGHMDLLNRWGKDEPEVLEDSIKSSLAEMQRMETLINEMLNLTRAEQIPIENVQEVT

EVGGLVHRVFDNFKLIHPDFIFTLDDDLTQDANVKVRQDHMEQILIILADNAVKYSLDRKEIHFALSQTT

NRVEIGIQDFGEGLSSEDAARVFDRFYRVDKARSRAKGGNGLGLSIAQRLVKAYGGEITLESALGSGSVF

TIRLPLQRENKNDQKLSN

>gi|125623752|ref|YP_001032235.1| sensor protein kinase kinA [Lactococcus lactis subsp. cremoris MG1363] llmg_0909 llac678

MNANKKMKKLFTFNKKKETVEESSAKRSIMLRWAFANTVFCFITFTLFATLTYQLTISSFIKEEQRLLTG

SMDSAEEVLEQADAPLNSTNLNTYIEATSKIQNGESEGMSLGSIIGTRKAFYIYDLNHKLLYSTNRHDFG

FQKQGNNEMKEIQGENPGYLVQRKIVSKGTGQVVGYLQAFYDTTTYHRISNLLLVVLLILEIVALIVAQM

IGYFMANYFMKPLEKLYQGMQEMASDPTNDFKPIEIESGDEIEELAHVYNDMMLKMKAYLEQQNRFVSDV

SHELRTPLAVLDGHINLLNRWGKNDPEVLDESLQASLDEVDRMKKMLEEMLALARLENVDLSSEELDCDV

GKVSNHALKNFQLLHEDFEIVLDNQLIYPVHARISENHFEQGLRILLDNAVKYSPDDRKEIVITVSEDEQ

FVITSVSDKGIGIAEEDINHLFERFFRADKARNREIGGTGLGLPILARLAENYQGEIEVSSELGIGSTFT

LKFPKM

>gi|22537764|ref|NP_688615.1| sensor histidine kinase CsrS [Streptococcus agalactiae 2603V/R] SAG1624 saga678

MKNKKDQFIGVKQPLSKKLSQLVFILFFSLFTVFSVLVYTSATRYVLHREKINVGRSLEKTRVRLSQANS

SLTSDDILEILYNQVFADDIYPHKRQNGIVRTGESIDSILYVNQEMTLYDVNRKPVFSTLRTGMPTIGKS

MGKVIISKVADMEGFVGTKAIYSQKTGQLLGYVQIFYNLGRYYSMRQNIIVFLIMMEVLGTVLALVVINS

ATKRIVRPVKNLHDLMHQISENPSNLEIRSKVRSEDEIGELSRIFDGMLDQLEDYTRRQSQFISDVSHEL

RTPVAVVKGHIGLLQRWGKDDPEILEESLAAAYHEADRMSLMINDMLNMIRVQGSLELHQDEVTDLSSSI

SVVIENFRILREDFQFIFENNISDIVWGKIYKIHFEQALMILIDNAIKYSPSYKEVSVVLSVDNDFATVV

VKDKGEGISDEDIEFIFDRFYRTDKSRNRESTQAGLGIGLSVFKQIMDAYHLKVDIKSELNQGTEFIVRI

PIKKFEETKEI

>gi|56420691|ref|YP_148009.1| two-component sensor histidine kinase [Geobacillus kaustophilus HTA426] gk2156 gkau678

MKREAIRRLSLKGKLTVLSAGAIFITYFVFTFLQYHMVKQWLLKEEEKTMEQTVAEIETYYAEKRNLSWE

GIRRSRAFLEKLNERYQLIRVTDEKGNVIVSVSNGAAVSLSPSEMPDKRKMDEHFINNERFLLLRQPLHI

GNLHGTIEIARRLARFQQVTNTLFLIMTAIGFLAMAASAVAGRFVAQSFVRPLQTLAKTMADIQNNGLKR

RIDVPPARDEIAELMVMFNGMMDEIERSFAMQRQFVEDASHELRTPLAILQGHLSLLQRWGKHNPEILEE

SLEAAVKEAERLKRLVLELLDLSRAEAITVPDEVAPMDAAAAVGQVVKNFRVLHPDFQFLIDQPDKPIAR

AAMAKHHFEQLLFILLDNAVKYSQRVKQIAISLREEGPFVIVSIRDYGIGIPKEELKNVFLRFYRVDKAR

SREQGGAGLGLSIAKEIIDKYGGQIALESEVGKGTTVELAIPKAE

>gi|15924404|ref|NP_371938.1| putative protein histidine kinase [Staphylococcus aureus subsp. aureus Mu50] SAV1414 saur678

MTKRKLRNNWIIVTTMITFVTIFLFCLIIIFFLKDTLHNSELDDAERSSSDINNLFHSKPVKDISALDLN

ASLGNFQEIIIYDEHNNKLFETSNDNTVRVEPGYEHRYFDRVIKKRYKGIEYLIIKEPITTQDFKGYSLL

IHSLENYDNIVKSLYIIALAFGVIATIITATISYVFSTQITKPLVSLSNKMIEIRRDGFQNKLQLNTNYE

EIDNLANTFNEMMSQIEESFNQQRQFVEDASHELRTPLQIIQGHLNLIQRWGKKDPAVLEESLNISIEEM

NRIIKLVEELLELTKGDVNDISSEAQTVHINDEIRSRIHSLKQLHPDYQFDTDLTSKNLEIKMKPHQFEQ

LFLIFIDNAIKYDVKNKKIKVKTRLKNKQKIIEITDHGIGIPEEDQDFIFDRFYRVDKSRSRSQGGNGLG

LSIAQKIIQLNGGSIKIKSEINKGTTFKIIF

>gi|116496107|ref|YP_807841.1| Signal transduction histidine kinase [Lactobacillus casei ATCC 334] lsei2680 lcasei26

MKKRKQNQSSAAIMQRSLVSMVATLTLALGLLIVAAVGHQLYEQVVLSTDHIAASLKQTVIDGDDDWENW

RKNSTLDTSSTYVHVHNMRQDAKVKNYYSPATKKVQLAKSFPFLTQLRYQANQGLFYYQEVHAKGIRYEI

WQSMNSYLAVLIRVLVVTMIVLVLALVVSPIFIRRLAHRLTGPLSDLSQSTKHAAEHAKTGVEMLPVPDK

PTEVTDLAKDFNDLLKQLHDRQEQQKLFIMNAAHELRTPIATIRSHAQLIERHGRAHPEIVLKSVNYITE

ESRQMQQLIEELLQLSRADRLTLDTHLLNLSETVQHLAEKLASAFSQTIEVHVTPNMSVESNESAIEQIL

DNFVTNAAKYSPADTIIAVNLTRDDDGNAVLAVHDQGAGISAEDKSHVFERFYRSANVRGTVPGTGLGLA

IANQLATINNGRIKLQDNQPYGTIAELILPLATTKERHQLNES

>gi|42519059|ref|NP_964989.1| two-component system histidine kinase [Lactobacillus johnsonii NCC 533] lj1133 ljohn26

MNKKIRTTSQQLTQLFLTLFIIILFLVNLAFAGISTAFIYNNAHEQAEEVIDTISDNISDHDEHKNKNEW

KLFLNAYLARQSNDAISLKTPKGKTVYSEDGEELFEKVKNHRNYNNVVFMENSVYYLRTANEDGYRISVI

LNVDDLFTLITHLLLAIIILNLIAIICSIPLIKRFSRKWSQPLEKIDQDIEAIEHQTTAKKKVFVPKQPA

EIHHLAQSFNKLLEYQDKAIQREQQFVTDASHELKTPLAAIRGHINLIKRRGKEHPEIVAKSLQYIDSES

SRMEILVNELLELGRAKKQHQSIDPIDLVPIVQKESEILEQEYDTCSVYISSPIKLFYPIEIKDFRLIVH

NLLDNAAKYSQDNAKIKVELQKDNGYLILRVKDQGIGIRKENYDKIFDRFYREDQAHSSQIKGTGIGLAI

VKDIVTKYHGKISVSANTPKGVIFEIKLPL

>gi|58337918|ref|YP_194503.1| sensor protein [Lactobacillus acidophilus NCFM] lba1660 lacido26

MKKNTTSAQLTRLFVGLFVAILLLVNIAFLIISSSYIYYHAKSQSEQVIQAVEENLEPKYDWSSLLDAFL

AKQDDDAIILTTPTGRTYYSEDAHETFKVIGQKRHYQNFVFAKKHVYFLNQERHRAYQIHVAINVDELLN

LITWLLFTMLGINIAAMLISIPLIRRLSYRWSRPIQNMNMEIKDIRKNGKKDQITVPTQPLEIKNLAKSF

NNLLAFQKKALEREQQFVSDASHELKTPIAAIRGHVNLIKRRGKSNPEIIPTSLNYIDVESKKLETLVNE

LLTLGRIDHYTNNDQNTDLVKIVQEVISEVQTVYPHTIQAKLPNQLIYSISTTDFYNIAHNLIENAAKYS

PENSDIDITLVEKDKHIIFKVADHGIGINSENKDKIFERFYREDNSHSSKISGSGLGLAIVQTEVNKYHG

KIVLKDNTPQGSIFIVSLPQK

>gi|116332986|ref|YP_794513.1| Signal transduction histidine kinase [Lactobacillus brevis ATCC 367 lvis_0316] lbrev26

MSTKTPPRTYADIIRHSITSLLLTIGVIIVITVSITLTIDQLVRAQDQAAKLSASLQTTQVSNFKDWLTI

NRSSGLNSHNTFVLIKNANGTTTSLLPHAQQLAKANTWSVPFTHLVYIKGWGLFFSETLRTGSRTYALFI

GMHVLIGNLHVLISVLIIAIGLSLLIGLLYVRRLAKRLSAPTLTLAQVAKQAAANSEATQPELPQPSEPV

EIGQLASDFNQLLAAQNGRLQRERQFISDASHELRTPIATIRGNLKLIERRGKQHPEIIPESLGFIDQES

LRMQHLIENLLHLSRANRAEISLHPVDIDPLVQSVIAHYQPLIDQPITYDGPATPTMVLADTDILHQVLT

ALLDNAHKYGAPDQSIDVTVAKQAQHVTLSVADHGPGISTADRPHIFERFYRVDESRSNKIEGSGLGLAI

VAQLVRLSKGTITITDNPPHGACFIISLPSANEN

>gi|28377206|ref|NP_784098.1| histidine protein kinase; sensor protein [Lactobacillus plantarum WCFS1] lp_0282 lplan26

MPKNNTTPTYEQLINKAIRRLMLTITLLVSLITLLMVGTQQTINTTREARDLMSSLNQANIKDVPSFIQW

NNTTTRNTKQPAFIRVTTKKATLTTATKTKRTIITTPSSKSFFTKQQTKLVGPLVYVRGFGFFLSYSEKD

GHNTYQLWVSLRRLFNSLLILILLMLAVTCAALALGTWWAHRLALRLSDPTIKLLHATKYTSNNPDSDQP

TLDVPASPREINDLGRAFNELLVTQNQRLQHERQFISDASHELRTPIAAIRGNLSLIKRRGDAHPEVIPE

SLQFIDEESLRMQHLIENLLHLSRADRAKLVLTDQDLSLLMLSLAEHYQPSIDQPLTLAIDPNIHVQGNA

EMLQQIVVALLDNAHKYSPHDQPITLSLHQTDQAVELAVADHGEGIPDDQKQAVFQRFYRVDQSRSQAIE

GSGLGLAIVQQLVTLNQANIKITDNQPSGSIFTVSWQL

>gi|116490564|ref|YP_810108.1| Signal transduction histidine kinase [Oenococcus oeni PSU-1] OEOE_0489 oeoe26

MRKVKNFFHNLLAFIRPLKNSSALIMTRSYFFLLAIITFSMVISVPTVLGYQLIRTQVQNANQYIKSLKK

TNIDSAHDWNIWIRNNTFDSDVIFIKAQTNKKKTFYSDGASHVASKNYKHLFFSKKYILDSKNWHDFNHY

KLYYTKTVTQYGNHFTVWINLYKVFNIIKITLYTLLIIICLVLIFGVWLIWQLSQRLTTPLSDLSSATEK

VSLKIKQSNQRMEDRLPEPTSPTEVNELAVSFNQLLDVISEKEVREKQFVSDASHELKTPIAAINGHLKL

IKRRGREHPEIIENSFDYISNETRRMQHLVQNLLDLSHAERDELNITKINLSELLVEISKNVPFPQKIKT

KFSSNIYIKADLDQIHEIILAVLENSSKYSPLDCPIILNIEQNSGKTIMRIIDFGKGISANDKKHIFERF

YRADASRSNSIPGSGLGLSIVKELANRQHIKINVLDNHPRGTIFELSLLR

>gi|125624322|ref|YP_001032805.1| sensor kinase protein [Lactococcus lactis subsp. cremoris MG1363] llmg_1518 llact26

MKKTESKTSANIITETFVRIFLIFTLLVTIIVSGIVAFTSLQIRESEGTSLISSVQEAAENGNVNWDEFK

LDSEKDEKSTFVRLTRASGDIDESHGTSHFLKSRLSWGNFSLSKDVIFWYSSETAKNGNKVELWLNINIV

VKSMIRAILVIVAVMIILFFIALILIRKAANTISQPLTDMMLATDEKEVKQLPVSKNPIEVRQLSQSFNR

LLNRLNQKIENEQQFVSDASHELRTPVAAIRGHVTLLKRRWHEHPEIIDDSLSYIDEESLRMKGLIENLL

TISRGNHLEVNKEKFNLTEFTDKVVSEIQPALSQKITFNGKSEIFVRADKMSLYKIIIVFIENAGKYSPQ

DSEIKVKISSENHQVELQVADEGVGIPEEEKINIFERFYRIDKSRSSEIPGTGLGLAIAKQYAQLNHAEV

FVTNNTPKGSIFHLLMESIEN

>gi|20807497|ref|NP_622668.1| Sensory transduction histidine kinase [Thermoanaerobacter tengcongensis MB4] TTE1017 tteng26

MVKMKIKLLPQRITLKIALLYSAVFSFVLISLNASVLYTLKYYLIYQSMEQVSTQAEVIKAKLSEAKQNM

DGALKDTAFSGIPDENIYVKIFDSKGRLLYLSKKLERVAIPYNTNLEVPVKVDEFDKDLVYLNTVFKKGD

ATFYIQVIKDMKNEYAFLKLLFILMFFADGAGIFISFVTGYFVTKRALRPVDYMIKEVKDIDAKGLNKRL

KVYGEEDELTRLAKTFNDMLDRLEESFARQNRFVSDASHELRTPISVIKGYIDMLDRWGKDDREVLEEGI

KAIKKETLEMESLVEKLLFLAKGDDRSIKLERESFDLKEIAEEVVREIKLIYEGKNVSLKGENVRINADK

KLIKEVLRILLDNAAKYTSKNGNIEIEIGSGDEAYIKVKDDGIGIPEEDLPYIFERFYRVDKARSKDTGG

TGLGLSIAKWIVEEHGGVIGVKSEVGKGTEFTVMLPLINDSK

>gi|16078391|ref|NP_389209.1| hypothetical protein BSU13260 [Bacillus subtilis subsp. subtilis str. 168] BSU13260 bsubti26

MKLKTKIHLYTSISLLILLILVHTAVYLIFSSALTSKDAARLADETDNIAEALRAAETEGVALQDMLQAY

LPANGMVRVVNGDQKAVMTITKEKAYKDFPLSFHSGETADVRKPDGKLFAEAAVPVIWTDGQVVSLQLVE

RLENTEESLFLLKIILIAASAAVCIASFFAGSLLARRIINPIRRLMITMKDIQRDKEFKTISLEGQSNDE

LYQMGLTFNEMAMMLKEHYDKQQQFVQDASHELKTPLTIIESYSSLMKRWGAKKPEVLEESIEAIHSEAV

HMKKLTNQLLALAKSHQGLEVDLKTIDLIKAARAVMQTLQSVYQRDILLETDKESLLVKADEERIKQLLT

ILLDNAIKYSEKPIEMSAGTRNGRPFLSVRDEGIGIPEEHIPHLFERFYRADEARNRKTGGTGLGLSIAK

QIADEHGIELSVKSKPGQGTAVTMQFSEQNGGGR

>gi|23098050|ref|NP_691516.1| two-component sensor histidine kinase [Oceanobacillus iheyensis HTE831] OB0595 oihey26

MIFRKLINFFSSITLRKKIIFFSTFFMLILILIVNTAIYFLFYQSASEGELDQLTTHTDTLVEKIGENSS

VSLEEMKDLLTAYLPTNGMIRVIDANENKLIDQKKPEYTEVPIEYTTTERREVISSDQADVAMVSKPIIW

QNGEIVTIQVSDHLIHMSETMQILRYVLFFSSAIILIPTIIASILLSRFLLNPIQKLKETMQTNISKRDW

KKIDIENRSKDELYEMEVTFNEMIDSLKENYEKQEIFVSNASHELKTPIQIIKSYAQLLSRRGEGNPELL

QESTEAIDSEADRMQNLVEQMLALAKNQQEQVVETVDMKELLQQVADTFQKAYAPREIILFTDEKTKYMW

QGNPDQIKQVIYILLTNALKYSEKEVNIRLQQKSDEMIVSVQDFGEGLAEQDQQHIFERFYRVDRARNRE

TGGTGLGLAIAKVIVETYQGEITVQSKLGEGSIFHIHLPNMDQ

>gi|116496229|ref|YP_807963.1| Signal transduction histidine kinase [Lactobacillus casei ATCC 334] lsei_2807 lsei807

MNKKIRFFQSIHFKIAIVFILLLVVTLELIGAYFVKTLEQQNIEQFKTSVNVDSYIQDKLAADLLRTDTE

GANDDIKSTLSQADIANSADLQVVDAKGTIRGDSDINAQTNVGQKSANSSIKNTLYSGQQYEGYEYRDRT

GSTYYKVIPIKNPNATGDNNSVIGAIAISASMEQAYDSINKIVGIFLISSLVAGLLGTLLSIVISRAITR

PIDEMKKQAIRMARGDYSGQVRIYGQDELGQLAVAVNNLSVRVEEAQEASEAERRRLDSVLAHMTDGVIA

TDRRGNVIIINQTALNFLNTKNEDVIGVSILKLLNIAKDYTLRDLLEHQTELLLDFSEQLDHELILRVDF

SLIQRETGFISGLVAVLHDVTEQQKIDRDRKEFVSNVSHELRTPLTSVRSYIEALSDGAWKDPKLAPRFL

NVTQEETDRMIRMINDLLSLSRLDSGTSKFNLETVNLNEFFNYVLDRFDMMLKTDNEHTGEAKTAQPLPQ

PDGQKKRYSIKREITDQDLWVEIDTDKFMQVIDNIMNNAIKYSPDGGTITARLFQTHDHVILSISDQGLG

IPRQDLGKIFDRFYRVDKARSRKQGGTGLGLAISKEVVEALHGRIWVDSQEGRGSTFYISLPYEPITDGG

DDWDEA

>gi|116493524|ref|YP_805259.1| Signal transduction histidine kinase [Pediococcus pentosaceus ATCC 25745] PEPE_1796 pepe807

MNKRTNFFKSINFKIALVFAMLLVVTLEVVGVIFVRQLETQNLNQFKTQVQLQPYVENEISTQLERANTK

SANSQISDIIGNINNQNITEIRVIDAKGVIRGTSNSTNQNIVGQKTTDRNVKDVIYNTRTYQQVSYNKTT

NTRYFVSIVPLINTAGATNNLTGVVYIRANLESVYQNVNNITLIFVVAALIAITIGLILAVLISRAITRP

IEEMRQRTIQIARGDYSGQVQIYGDDELGQLAEAVNELSVRVEESQESTESERRRLDSVLGYMTDGVLAT

DRRGRITIVNEMATDFLNLENDQIVGKSILDILDLRGTFTLRDLLENQEQVVLDLSDDDQDLILHASFAL

IQRESGFISGLVCVLHDVTEQQKIDQDRKRFVSNVSHELRTPLTSMKSYIEALVEGAWKDPEVAPNFLKV

TQEETDRMMRMINDLLNLSRMDLGTARLEREYVNLNELFNHILDRFDMILKNSDKPDKNYSIKRDFTRRD

IWVEVDTDKIQQVLDNIMNNAIKYSPDGGIITCRLLETHNHVIMSITDQGLGIPKDSISHIFDRFYRVDK

ARSRAQGGTGLGLAISKEVIQLHGGRIWVESREGEGSTFYISLPYEPFEEGDTWE

>gi|116490222|ref|YP_809766.1| Signal transduction histidine kinase [Oenococcus oeni PSU-1] OEOE_0106 oeoe807

MNNLSNFISRFFRSVHFKIALIFTVITLITLGLVGAFFVNQYSSQLIQSFDDRVTVPSTIKSQLISDLQN

SNKKAANQDINQALSTLGGSTDQDYVYVVDKSGVIRGSLTVEMKQLVGQQIDSSISGKKVQSNIYHALSS

SKDYVRRFRSESLGRRYETLTIPLESKPGDRSSTVGVFVYTASMESIYESIANITNMFVGALFIPLLMAI

MIGFLMVHLLTRPITELAEQTEQITKGNYSIKNSIKSKDEIGILATQINDLSDTIASETATSNMESDRLN

SLLSNMTDGVLAITRDGTITVINQAALDLLHIPRTYKAVGKNISDLLKLVDKKITLRKLFQSIRGFVVEP

DGPDGVTLNAVASLVRRQSGFISGAVVVLRDVTRQLKLENDQKNFVSNVSHELRTPLTSVNSYIETLQEG

ALTQKKIAREFLGVAHDETQRMIHMINDLLELSRMDQGRTKMDTEAVNFTKFINYVLDRFDVMFKQGHSM

HSDNHYKIIRNYDQKKSCYVEIDSDKFMQVVDNVINNAINYSPDGGNIEIGINETDDSIILSIKDHGLGI

PKKDLENVFTRFYRVDKSRARKQGGTGLGLAISKEVVEALHGKIRVESTVNVGTTFFISLPKVDTSSSRF

DFS

>gi|161506706|ref|YP_001576656.1| putative histidine kinase [Lactobacillus helveticus DPC 4571] lhv_0096 lhel807

MKKLREKLNATFNSINTKLAVVFMLMLLATIEVIGAYFTRQLEQNSIENFQSSIQIQTIVSNQLANQLAS

DSKNANDRLNQIVNDYNNDSISEIIVVDNKDTIRAVSNLNDKSKVGQRMNNTDVKQVISTGHQINKVVND

HGNYMIQISPLTSGNGSNNNVGALYVKASMQDVFDNLRQISLTFLIASLIAALLGAFLALVISRAITQPI

EEMQKQALHIANGDYSSQVKIYSNDELGQLAKAFNTLSVRIEQSQEESDSERRRLDSVLSHMSDGVLATD

RHGNVSVVNHMALSYLNADEDEIINRPIAEVLGLKDTSSQDLISSQKEIVVTVKPGTRDEIILHASFSLI

KRVTGFVSGSVCVLHDVTEQQKNENSQRQFVSNVSHELRTPLTSLQAYIEALNEGAWKDPEIAPKFLEVT

QQETSRMIRMINDLLSLSRMDRGVSKMDLEFVNFNDFVSHILNRFDMIVKTDKNKGHKKKYTIKRELGNQ

ALWVEIDTDKMMQVIDNIMNNAIKYSPDGGVITVRLTHSQGHVILSISDQGLGIPRKDLGKIFDRFYRVD

KARSRAQGGTGLGLAIAKEIVEAHHGRVWADSSEGKGSTFYISLPYETMTEEDDWDEV

>gi|116619022|ref|YP_819393.1| Signal transduction histidine kinase [Leuconostoc mesenteroides subsp. mesenteroides ATCC 8293] LEUM_1949 leum807

MKMTSITKFFQSIRFRITLVFVLLMIIALELLGAFFVRQMERQNLRTFQQQITLPKYVTDPINTALRNSN

SDEGNHEIQDVLSGLNNTNIEEVQVIDKTGTIRGTTSVSGQQTISQKTTDINAQKALAGDQSYVATRTIN

ENGERKEIMTTPLGVTTGRLKDVSGVIVVKASLLPVYKNINSVMRLFIIAGLIALVASVALALFLARTLT

RPIEQISEQTTKIAEGDYSMVNHIYGSDELSQLALSVNDLSTRIEESTETVNAERNRLDSVLTHMADGVL

AANRRGEVTIINQAAADFVGVNREDALGQNVVSLLRLEGKRTLRDILENLNDFRVDLSDDSKELLVQIYV

SLIKRQSGFISGLVVVLHDITEQQRIDSERRMFVSNVSHELRTPLTSVRSYVDALAEGAIDDPEMAQNFL

GVVQDETQRMIRMINDLLELSRLDQGTMEVKLEVINLNSLFNFVLNRFDMILESDAKNENTITKNYNILR

EFTKEDVWVEVDPDKFTQVLDNIMNNAVKYSPDGGTITARLIKTKTRAILSISDQGLGIPRKDLDNIFNR

FFRVDKSRSRAQGGTGLGLAISKEVVEKFNGRIWVDSVENKGSTFYISLAYVDEEPEDDWDAE

>gi|148543263|ref|YP_001270633.1| multi-sensor signal transduction histidine kinase [Lactobacillus reuteri F275] Lreu_0020 lreu807

MNSKLKFYQSIRFKIALVFVLILMLTLECVGAVFVRQLEHQNLNTFKQTIELPSYVDNSLAEQLSRSNTK

KANKQINQILSEVNNNNISEIRVVDSKSIVRGTSNADNRSAIGQKTTDQAIKATLLNNRSHTENLYDNSN

HTRYYVNVIPLVDNSNNNVVGAVYLRASLESVYSNINNITLVYLSAAMITIVLSLILAIIISQEITRPIE

EMRKQTLRIARGDYSGQVKVLGNDELGQLAGAVNNLSVRVEEAQESSDSERRRLDSILSHMSDGVLATDR

RGNVTIVNNMALQLLGVENEDDLIGKSIIDVLDIRHDYTVRQLVNGEQREMIIDMSNSGNNLILNAYFSP

IQRESGFVSGLVCVLHDVTSQQKEERERKQFVSNVSHELRTPLTSVRSYVEALSDGAWQDKEIAPQFLKV

VQDETDRMIRMINDLLSLSRMDAGTTKLNLEYVNINELFNYILDRFDMIIKKEEDPKKKKYTIERYFTKK

DLWVEIDTDKFTQVIDNIMNNAIKYSPDGGVITTRLLETHNHVILSISDQGLGIPRKDLGRIFDRFFRVD

KARSRKQGGTGLGLAISKEVINMLGGQIWVDSVEGKGSTFYISLPYVPYEEEDWDDEEAEK

>gi|90961026|ref|YP_534942.1| Two-component sensor kinase yycG [Lactobacillus salivarius subsp. salivarius UCC118] lsl_0036 lsal807

MNKKVRFFHSIHFKIALVFALLLLITFQIVGAIFVRQLKNDNLKSFKQRVELSTYVENSLIVALSRTDTE

GANTKIKNILDDINNANIQEVQVIDAKGTIRGDSDVNSRSLIGQKTTNSAVKGVLYTGRTYTETTYNRND

NKQYYISITPLLNVSSNNNAVVGAIYTRADMASVYDSVNNVVVIFASASLIAIVIGMVLAIIVSSSITKP

IEEMKRQTARIARGDYSGHVHQYGNDELGQLAQAVNNLSIRVEETQELSESERRRLDSVLSHMSDGVIAT

DRRGGITIVNDMAMNYLDISEGEVLGKSILDILDIRNQYTLRDLLEKQDEVVLDMSGEKDLILNAYFSVI

QRESGFISGIVCVLHDVTEQQKIDRERKQFVSNVSHELRTPLTSMRSYIEALNDGAWRDEKVAPEFLKVT

QDETDRMIRMINDLLSLSRMDSGTVKLDLELVNINGLVNYILDRFDMMLKKDKIDTRDIKTKNYTIKRDF

TKRDLWVELDTDKFIQVVDNIMNNAIKYSPDGGVITCRLLETHNNVILSISDQGLGIPKKDLGHVFDRFF

RVDKARSRAQGGTGLGLAISKEVIELHHGRIWVDSVEGKGSTFYISLPYEEYSEEDLWDEN

>gi|42518152|ref|NP_964082.1| two-component regulatory system histidine kinase [Lactobacillus johnsonii NCC 533] lj0066 ljoh807

MKKIKSVLNSINFKIAIIFMLLLLATIEVVGASFTRQLEQNSIQNFESSIQVPNIITNQIASQLSRANSK

SANQQLSQIISNYNLGDISQLMVVDNKGVIRAVSNVNDQNRIGQRTSNADIKSVLSNGKQVSKVINDNGN

YMVQISPLTSANGTNTPVGAIYVRASLQGVFNNLRQVSIYFLIASLIAAVLGAVVALVISRAITRPIEEM

RKQALRVANGDYSGHVRVYAQDELGQLAEAFNTLSVRIERTQEISDSERRRLDNVLTHMTDGVIATDRHG

NITIINETALDFLGKTEKDVIGKPITNLLGLKDVTIQDLLSTQQELVVRVNDNTRDEMILHANFSLIQRV

TGFVSGLVCVLHDITQQQKNEREQQQFVSNVSHELRTPLTSLRAYVEALNDGAWKDPNIAPQFLHVIQDE

TERMIRMINDLLSLSRMDRGVAKMDLEWVNLNDFVNHVLNRFDMMLKSDTDKTHKKKYSIKREFPHQALW

VEIDTDKMMQVIDNIMNNAIKYSPDGGVITVRLLQAQKHVILSISDQGLGIPRKDLNKIFDRFYRVDKAR

SRKQGGTGLGLAISKEIVEAHHGRIWADSAEGSGSTFYISLPYEAISEEGENWDEV

>gi|116628743|ref|YP_813915.1| Signal transduction histidine kinase [Lactobacillus gasseri ATCC 33323] lgas_0065 lgas807

MQLGQMKKIKSVLNSINFKIAVIFMLLLLATIEVVGASFTRQLEQNSIQNFESSIQVPNIITNQISSQLS

RANSKKANQQLSQIISNYNLGDISQLMVVDNKGVIRAVSNVNDQNRIGQRTSNVDIKSVLSNGKQVSKVI

NDNGNYMVQISPLTSANGTNTPVGAIYVRASLQGVFNNLRQVSIYFLIASLIAAVLGAIVALVISRAITR

PIEEMRKQALRVANGDYSGHVRVYAQDELGQLAEAFNTLSVRIERTQEISDSERRRLDNVLTHMTDGVIA

TDRHGNITIINETALDFLGKTEKDVIGKPITNLLGLKDVTIQDLLSTQQELVVRVNDNTRDEMILHANFS

LIQRVTGFVSGLVCVLHDITQQQKNEREQQQFVSNVSHELRTPLTSLRAYVEALNDGAWKDPNIAPQFLH

VIQDETERMIRMINDLLSLSRMDRGVARMDLEWVNLNDFVNHVLNRFDMMLKSDSDKMHKKKYTIKREFP

HQALWVEIDTDKMMQVIDNIMNNAIKYSPDGGVITVRLLQAQKHVILSISDQGLGIPRKDLNKIFDRFYR

VDKARSRKQGGTGLGLAISKEIVEAHHGRIWADSAEGAGSTFYISLPYEAISEEGENWDEV

>gi|116513325|ref|YP_812231.1| Signal transduction histidine kinase [Lactobacillus delbrueckii subsp. bulgaricus ATCC BAA-365] LBUL_0112 lbul807

MKKEKTNLLKRAVSSIQTKIALIFMLLLLATIEIIGAYFTRQMEQTSINNFQTTIQLQSIVTDQLTTQLV

KNGKSADRKMNQIITDYSNDAISEIQVVDSKNVIRAVSNLSDKNRVGQLSNNGDIREVINSGQQKTQVTS

DQKGSFMVQIVPLTSGTNIVGAVYVRASMADVFNNLRNISMMFLTASLLAVVVAAIASMFISRAITRPIE

ELRQQAIQVADGDYSTQVKIYSNDELGQLGAAFNTLSTKIERSNEQSESERRRLDSVLSHMTDGVIATDR

HGNVSIMNQMAQDFLDVKAEDAIKKPISQVLGLGEDYTSQDLISNQSDMQVTVNAGTDDEMILHASFTLI

KRVTGFVSGAVCVLHDVTEQIKNEDEQRQFVSNVSHELRTPLTSVRAYIEALNEGAWKDPEIAPQFLDVT

QKETERMIRMVNDLLVLSRMDRGATKLEPEWVNFTDFVSHLLNRFDMIVEHDQGEAQSDTKEYVIKRDLG

NQALWVEIDTDQMMRAIDNIMNNAIKYSPDGGTITVRLTQNQNQILLSISDQGLGIPRKDLGKIFDRFYR

ADKARSRAQGGTGLGLAIAKEIVTAHGGRIWADSREGHGSTFYIALPYEPMDEEDEWDEV

>gi|58336427|ref|YP_193012.1| putative histidine kinase [Lactobacillus acidophilus NCFM] LBA0079 laci807

MKKLRIKLNTTFNSINTKLAIVFMLMLLATIEVIGAYFTRQLEQTSIENFQSSIQIQTIVSNQLANQLAS

DNKNANDRLNQIVNDYNNDAISEIIVVDNKDTIRAVSNLNDKSKIGQRINNTDVKQVISTGHQINKVIDD

HGNYMIQISPLTSGNGSNNNVGAIYVKASMQDVFNNLRQISLTFLIASLIAALLGAFLALVISRAITQPI

EEMQKQALHIADGDYSSQVKIYSNDELGQLGKAFNTLSVRIERSQEESDSERRRLDSVLSHMSDGVLATD

RHGNVSVVNHMVLTFLNAKEEDVINKPIAEVLGLKDTSSQDLISSQKEIVITLDEGTRDEMILHASFSLI

KRVTGFVSGSVCVLHDVTEQQKNENSQRQFVSNVSHELRTPLTSLQAYIEALNEGAWKDPEIAPKFLEVT

QQETGRMIRMINDLLSLSRMDRGVSKMDLEFVNLNDFVNHILNRFDMIVKTDKNKDHKKKYTIKRELGNQ

ALWVEIDTDKMMQVIDNIMNNAIKYSPDGGVITVRLTQNQNHVILSISDQGLGIPRKDLAKIFDRFYRVD

KARSRAQGGTGLGLAIAKEIVEAHHGRIWADSSEGKGSTFYISLPYEPMTEEDDWDEI

>gi|28377005|ref|NP_783897.1| histidine protein kinase; sensor protein [Lactobacillus plantarum WCFS1] lp_0037 lpla807

MLRFKRKNFFKTINFKIALVFALLLLITLEIVGAIFVKQLESKNIQDFKQSVQIPTYIDNSLSEQLTVSS

TRKANGQIKTILSNYNNTNVDEIQVVDSKGTIRGTSDVNDQSVVGQKTTDRNVKNAIYNNRTYTKSTYDT

QNNGRYYVSIVPLYSSNSAGNSSQLVGVLYVRANMKSVYSTINNIMGIFVIASLAAMVLGLGIAIIIARA

ITRPIEEMKQQTQRIARGDYAGQVRVYSDDELGQLAKAINNLSIRVEESQESTEAERRRLDSVLSHMSDG

VIATDRRGNITIINETASDFMDVTAEKAIGNSILDILKIRDDYSLRDLIENQDELMLDFSSNERDLILNA

YFSLIQRESGFISGLVCVLHDVTEQQKIDNDRKQFVSNVSHELRTPLTSLRSYIEALSDGAWKDPEVAPG

FLKVTQEETDRMIRMINELLSLSRMDSGTTRVDMELVNINEMFNYVLDRFDMILKKDDNPAKYYTIKREF

TKRDLWVEIDTDKFTQVLDNIMNNAIKYSPDGGVVTCRLLETHNQVIISISDQGLGIPRADLGHVFDRFF

RVDKARSRAQGGTGLGLAISKEVVQMLGGRIWVDSVEGKGSTFYISLPYEPYEEEDLWDDDSQA

>gi|116332710|ref|YP_794237.1| Signal transduction histidine kinase [Lactobacillus brevis ATCC 367] lvis_0028 lvis807

MNSKIRWFQSIHFKIALVFALLLLLTLEIVGAVFVRQLERQNLNTFKAQQVVQTHIVNRLSDELSMSGMT

KSNKQIKSTLSEINNTNNAQIRVIDNKGIIRGTNEVDNQGIVGQKTTDNNIKNAIYNNRDVVRTTYSSQS

NSRYYTTITRLIRTQGNNSELVGVIYMRSSLDSVYQNINQITLIYVSATFVAIILGLVMAIIISRAITRP

IDEMKKQTLRIARGDYSGHVHVYGNDELGQLASAVNNLSVRVEEAQESTESERRRLDSVMAHMTDGVIAT

DRRGNVTIINETASSFLDHEPDDAVGKSILDLLDIRDDYTLRELLEDPDDLVLDFSTKEHDLILQAYFSL

IQRESGFISGLVCVLHDVTEQQKIDQARREFVSNVSHELRTPLTSVRSYIEALSEGAWKDPKVAPDFLKV

TQEETDRMIRMINDLLTLSRMDSGTQKVDLEMVNLNELFNYVLDRFDMMLKQDDQPEKTYTIKRDFTKRD

LWVEIDTDRFTQVLDNLMNNAIKYSPDGGVVTARLLETHNNVILSVSDQGLGIPRADIPRIFDRFYRVDK

ARSRAQGGTGLGLAISREVVQMLGGRIWVDSREGRGSTFYISLPYKPYDEEDLWDEDN

>gi|81427691|ref|YP_394689.1| Two-component system, sensor histidine kinase [Lactobacillus sakei subsp. sakei 23K] lsa0078 hpk3 lsak807

MNKKIRLFQSIHFKIAIVFVLLLLVTLEVIGASFVKQLEYQNVKDFKQSLQVTPYVQNQLSNELMSNSGN

ANKNIKDIIGDMGNSAEIQVVDNKGTIRGVTNLNTQSTVGQKTRNPRVKQAIYSGHTEQEVAYSESQGSY

YTAIQPLTDPNGDSNTVVGAVYIRASMEEVYKGISSITVIFLTSSLVAGLLGMALSIVISRAITRPIDEM

KKQAIQMARGDYSGQVRIYGQDELGQLAIAVNNLSVRVEEAQEASESERRRLDSVMSHMTDGVIATDRRG

NITIINETGQDFLNVTSDGVLGQSLLKVLKISEEYTLRDLLETQQVMVLDFSDQQDHDLILHVDFSLIQR

ETGYITGLVCVLHDVTEQQKNEREQREFVSNVSHELRTPLTSVRSYIEALNDGAWQDPEVAPNFLKVTQE

ETDRMIRMINDLLSLSRMDQGTAKMNLEFVNLNEFFGYVLDRFDMMIKRDQEHDNNASPRGTGKLPTDDP

NKKYSITRDFTKRDLWVEIDTDKFMQVVDNIMNNAIKYSPDGGVITCRLLETHRHVILSISDQGLGIPRK

DLGKIFDRFYRVDKARSRKQGGTGLGLAISKEVIEAHNGRVWVDSQEGKGSTFYISLPYEPIDSDGGDWD

EI

>gi|184154492|ref|YP_001842832.1| two-component sensor histidine kinase [Lactobacillus fermentum IFO 3956] laf_0016 lfer807

MNSKIKFYQSIHFKIALVFALMLMLTLEVVGAVFVRQLEHQELANFKQQIELPSYIDNSLATQLTSTDTK

TANKEIKTILARVNNTSITEIRVVDAKGIIRGTSSSGNQGIVGQKTTDTVIKNTLVNNRSHTENLYDSAN

HNRYYVSVVPLLASGNANNVVGVVYMRANLEGVYSTINSISLIYLSAALITIVLGLGLAVLISREITRPI

EEMRKQTLRIARGDFSGQVKVMGNDELGQLAGAVNNLSVRVEESQESSDAERRRLDSVLSHMSDGVLATD

RRGNLTIVNNTALQFLNIADEEQVLGKSILDVLKIRRQFSIRHLVDEDQDQVILDDLNEDLILSAYFSPI

KRESGFVSGLVCVLHDVTSQQKEERERKEFVSNVSHELRTPLTSVKSYVEALSDGAINDPELASRFLGVV

SDETERMIRMINDLLSLSRMDSGTAKLNLEYVNINELFNYILNRFDMIIKNEAADPSQKKYTIVRYFTKK

DLWVEIDTDKFTQVVDNIMNNAVKYSPDGGVITARLLETHNHVILSISDQGLGIPRKDLGHIFDRFFRVD

KARSRKQGGTGLGLAISKEVVNLLGGQIWVDSVEGKGSTFYISLPYVPYEEEEWDDQAD

>gi|170016544|ref|YP_001727463.1| Signal transduction histidine kinase [Leuconostoc citreum KM20] lck_00186 lcit807

MNSTTNFFKSIRFRIALVFVLLMIIALELLGAFFVRQIERQNLTTFQQQIALPKYVTDQITTALKKKDSN

EGNHEIQDILSSLNNTNLQEAQVIDKTGTIRGTLSVSGQQTIGQKTTDLNAQKAIAGEQSFLKSRTVNEN

NERKELLTTTLGSNNGRGKDIVGVLVVKASLAPVYKNIDSVMRLFVIAGILALMVSIALALFLARTLTRP

IEQIDEQTTKIANGDYSMVNHIYGSDELGHLAQSVNELSTRVEESTETVNAERNRLDSVLTHMADGVLAA

NRRGEVTIINQAAADFVGVDRDAAIGQNVIELLQLKGKRTLRDMLENLDDFRVDLSDDSKDLLVQAYVSL

IKRQSGFISGLVVVLHNITEQQRIDAERRMFVSNVSHELRTPLTSVRSYVDALAEGAINDPEMAHNFLGV

VQNETQRMIRMINDLLELSRLDQGTMAVKLEVINLNSLFNFVLNRFDMIIENDAKNTETPNKNYSIQREF

TSEDVWVEVDPDKFTQVLDNIMNNAVKYSPDGGVITARLIKTKTRAILSISDQGLGIPKKDLNNIFNRFF

RVDKSRSRAQGGTGLGLAISKEVVEKFNGRIWVDSVENQGSTFYISLAYVDDAELQEADWDAE

>gi|16802334|ref|NP_463819.1| hypothetical protein lmo0288 [Listeria monocytogenes EGD-e] lmo0288 lmon807

MHKMRFFQSVQFKLVIMYLLLIIVAMQVIGAYFVRELEGQLEKNFQDSITNSITLLDYNAREEIIKNSDN

SVKLQNDIRELLVDYSRASSNLIEVRIVDDKGKILGTSNLNNQGIVGQKSNDPLVKRTLSLGTTSEDKIY

KDESNKNNRVWVNVSSIKNKGQVIGAIYLVADIESVYKQVDDITNIFITGTLIAMIITAVLGILLSRTIT

KPIVEMKRQAYAMARGNYSRKVKVYGVDEIGELADSFNTLTKRVQEAQAMTEGERRKLSSVLAYMTDGVI

ATDRRGKVILINTPAEKMLRVKHESANGRSIIDVLDIGDTYQFEDLMEVDGSLTMDRSTLDKPYVLRANF

SVIQRETGFNNGVIAVLHDITDQEKVDQERRDFVSNVSHELRTPLTSMHSYLEALSDGAWEDKEIAPRFL

EVTQNETERMIRLVNDLLKLSRMDGGREQLEKSFVNFTDFFNHIIDRFEMMKKETIMFKRHIPREPVIIE

IDEDKVMQVLDNIISNANKYSPDGGRISFYLKKFEDEIEVSIADEGLGVPDEDLANVFDRFFRVDKARSR

EMGGTGLGLAIAREVIEAHGGRIWAERNKNKGTIIKFTLPYSDLPEDDWE

>gi|16081092|ref|NP_391920.1| hypothetical protein BSU40400 [Bacillus subtilis subsp. subtilis str. 168] BSU40400 bsub807

MNKVGFFRSIQFKITLIYVLLIIIAMQIIGVYFVNQVEKSLISSYEQSLNQRIDNLSYYIEQEYKSDNDS

TVIKDDVSRILNDFTKSDEVREISFVDKSYEVVGSSKPYGEEVAGKQTTDLIFKRIFSTKQSYLRKYYDP

KSKIRVLISAKPVMTENQEVVGAIYVVASMEDVFNQMKTINTILASGTGLALVLTALLGIFLARTITHPL

SDMRKQAMELAKGNFSRKVKKYGHDEIGQLATTFNHLTRELEDAQAMTEGERRKLASVIAYMTDGVIATN

RNGAIILLNSPALELLNVSRETALEMPITSLLGLQENYTFEDLVEQQDSMLLEIERDDELTVLRVNFSVI

QREHGKIDGLIAVIYDVTEQEKMDQERREFVANVSHELRTPLTTMRSYLEALAEGAWENKDIAPRFLMVT

QNETERMIRLVNDLLQLSKFDSKDYQFNREWIQIVRFMSLIIDRFEMTKEQHVEFIRNLPDRDLYVEIDQ

DKITQVLDNIISNALKYSPEGGHVTFSIDVNEEEELLYISVKDEGIGIPKKDVEKVFDRFYRVDKARTRK

LGGTGLGLAIAKEMVQAHGGDIWADSIEGKGTTITFTLPYKEEQEDDWDEA

>gi|15923009|ref|NP_370543.1| two-component sensor histidine kinase [Staphylococcus aureus subsp. aureus Mu50] SAV0019 saur807

MKWLKQLQSLHTKLVIVYVLLIIIGMQIIGLYFTNNLEKELLDNFKKNITQYAKQLEISIEKVYDEKGSV

NAQKDIQNLLSEYANRQEIGEIRFIDKDQIIIATTKQSNRSLINQKANDSSVQKALSLGQSNDHLILKDY

GGGKDRVWVYNIPVKVDKKVIGNIYIESKINDVYNQLNNINQIFIVGTAISLLITVILGFFIARTITKPI

TDMRNQTVEMSRGNYTQRVKIYGNDEIGELALAFNNLSKRVQEAQANTESEKRRLDSVITHMSDGIIATD

RRGRIRIVNDMALKMLGMAKEDIIGYYMLSVLSLEDEFKLEEIQENNDSFLLDLNEEEGLIARVNFSTIV

QETGFVTGYIAVLHDVTEQQQVERERREFVANVSHELRTPLTSMNSYIEALEEGAWKDEELAPQFLSVTR

EETERMIRLVNDLLQLSKMDNESDQINKEIIDFNMFINKIINRHEMSAKDTTFIRDIPKKTIFTEFDPDK

MTQVFDNVITNAMKYSRGDKRVEFHVKQNPLYNRMTIRIKDNGIGIPINKVDKIFDRFYRVDKARTRKMG

GTGLGLAISKEIVEAHNGRIWANSVEGQGTSIFITLPCEVIEDGDWDE

>gi|23100906|ref|NP_694373.1| two-component sensor histidine kinase [Oceanobacillus iheyensis HTE831] OB3451 oihe807

MNKVGFFRSIQLKFIIIYILLLIIAVQVIGSFFTDRLNEELTRNFKTTVGERVEMLSHNLEQAFARDRSE

ESGTIESLEEEIRRIINDINRGNASTTINVVDNQSRVRGTNVLDDQNQIGQKLAHPMVRDALLFNSTDDS

IFYEKDTDSRVYVYVKPILGEDNMAVGAIFYKSSLEEVYGQIEDINNIFLQGSILAITISAIIGILVART

ITKPIMEMREQALIMANGDFTQKVNVYGRDEIGQLAETFNDLNSRLKHSYATIEEERRKLSSILANMSDG

VIATDNDGSVTLMNDAAAKLIGENPEDLIGDSVIDVLHLEGKEIDLSELHSNGSMIIDFSNEDTPFLIRA

NFSTVVDEEEEVTGFITVISDVTEQQKMEQERRDFVSNVSHELRTPLTTMKSYLEALSDGAWENKEIAPR

FLDVTQKETNRMIRMVNDLLQLSKMDSDELPMHKQREEFTSYLYQVLDRYEMNKPESIHMEKNIPNTKAY

VWMDRDKITQVLDNVITNAIKYSPDGGKIRVKLDIRRHYLLVSIQDQGMGIAYDKLDKIFERFYRVDKAR

NRKLGGTGLGLAITKELVEAHHGQIWAQSSEGKGTTILFTLPLMSSKRGDVR

>gi|58337701|ref|YP_194286.1| putative histidine protein kinase [Lactobacillus acidophilus NCFM] lba1430 laci430

MIQKFRWKFIGASVAALLLVLIITLGGLVGITRVQSQNEVNRVLTALVKNEGRLSPRNAQPAFGNQKDII

NRNFLGGQYNPEAVYQYRYFAVTVDPTQRINVINDNNVYKINNTEIKSVTKRALANRDQFGDVKIGQNRY

AYRVDKNAAGQTMIVFLNETLIFNRFWLLFRVSLVLGAFALLIFAAVLILVSGKAIKPIVDTYHKQQEFI

TNAGHELKTPLAVISANTEMEEMLGNNSEWNESTKEQVEKLSELINRLISLARTGENGEVALSKVDFSQI

VEDVTQNFKSVMKQNDLVYQVSIREGINVIAEKHSLTEVVNILLDNARKYCDPHGRVKVELVKSTLSKNA

ILRVINTYKEGKNKDYTHFFDRFYREDESHNSKKGGFGIGLAMAQELIHTFHGKISVNHREENIVFSVSL

KIVK

>gi|116629369|ref|YP_814541.1| Signal transduction histidine kinase [Lactobacillus gasseri ATCC 33323] LGAS_0711 lgas430

MRLMIQKFRWKFIGMSITALFIVLVITLGTLLGISYTQSHNEVDRVLTTLVNNQGQLTPRNAKPVFGNQK

DPINKNFLAGEYNPEAIFQYRYFTVASTKNNTPKIINDDNVYDVARKQIIGTSKQVFKDKVTSGSVNIGS

NQYAYRVGKDSKGNQFIVYLNESLIYHRFSLLFRVSILLGLIALIVFALILILVSKKAIGPIITTYHKQR

EFITNAGHELKTPLAIISANTEMEEMLGNNSEWNKSTKEQVDRLTRLINRLIALARTGETGEVVLNKVNF

SEIVKKNVTSFKSVMQKNDLKYNAMIMPDLYVKAERNILSELTNILLDNARKYCDRDGEVRVSLTKGKLG

TNAILRVANTYKEGKGKDYSHFFERFYREDESHNSKKSGFGIGLAMAQEIVQTFHGKIHVSHKDDMIVFT

VILKLAK

>gi|42519460|ref|NP_965390.1| two-component system histidine kinase [Lactobacillus johnsonii NCC 533] LJ1586 ljoh430

MSITALFIVLVITLGALLGVSYTQSHNEVDRVLTALVNNQGQLTPRNAKPVFGNQKDPINKNFLAGEYNP

EAVFQYRYFTVAETPQSNPRIVNDDNVYDVGRAEILSTSKRIFKDKTTDGVVTIGNNQYAYRVGKNQTGR

KFIVYLNESLIYHRFSLLVRVSIVLGIGALIVFALVLILVSKKAIGPIITTYRKQREFITNAGHELKTPL

AIIAANTEMEEMLGNNYEWNKSTKEQVDRLTRLINRLIALARTGETGEVVLNKVDFSEIVKKNVNSFKSV

MQKNNLKYSGMIMPDLYVKAEKNVLSELINILLDNARKYCDKDGKVQVTLTKGRLGTNAILKVANTYKEG

KGKDYSHFFERFYREDESHNSKQSGFGIGLAMAQEIVHTFHGKIHVSHKDDMIIFTVILKLAK

>gi|116513941|ref|YP_812847.1| Signal transduction histidine kinase [Lactobacillus delbrueckii subsp. bulgaricus ATCC BAA-365] LBUL_0803 ldel430

MIKKIRRRFIWLATLSLFLVMFFSFSSLIGISLFESSHEADQVLSIIVKNNGRLDEVQAKKRLGDQYNKE

GMFKYRYFTANINTKKQTLTVIDADHIIGYSINSVSSRKKQISRRLSAGQLKGTIHSNGVRFRYLAKKIS

ANKYYICFIDLSPLMQSALLLVRYALIFTFGGILFFVIIMTLLADKAIQPIKDAYYKQRRFITNAGHELK

TPLAVISANAEMEELLGNDDEWSESTKEQVVRMTGLINQLITLTRMSEPGELVLSKVNFSEIIKESADSF

KSVLAKDNKKYVVSIDPDLYINAEQRSLLELVNILIDNAQKYCDPEGEVMVVLAKSRLNQNAVLTVTNSY

KNGAGIDTNRFFERFYREDESHHYDQKQKNGFGIGLSMAQDLVAAFGGKINASWSDGVMNFTVTFKRVH

>gi|148543301|ref|YP_001270671.1| integral membrane sensor signal transduction histidine kinase [Lactobacillus reuteri F275] Lreu_0059 lreu430

MIQRFRYKFIAISTAALLFVILTIVGSICTLTYYQSHQEIERVLTILVNNDGQIPRKGIRTTDNSQPQFS

REGLHQYRYFAVLVDNKNQVTEVQDDHIATVTPQDARAMTDRLVRRKVHSGQLLYRGVNYAYKIRNKNGE

KVIVFLDESLLMARTRSLMHTGLILGVIVLVLYTIVLTLYSRRAIRPIIEAEQRQKEFITNASHELKTPL

TVISANNEMQEIINGENEWTTSTKQQVTRLTKLINNLVSLARMQEQPTLTMLPVNVSQIAGDVASSFKSV

ISTEQKQFNVQIDEGLVANADENTLRELLNILLDNANKYCDPQGEIDFTVSSSNHSKNIVITIANSYKDG

KSLDAKKFFNRFYRVDESHTQGKKAGFGIGLSMAQYIVKQFKGKITAKYTNGKLAFIVTLKEVGK

>gi|22537474|ref|NP_688325.1| sensor histidine kinase [Streptococcus agalactiae 2603V/R] SAG1327 saga430

MFRNLRLRFIGIAALAILVVLFSVVGVLNSANHYQTKNEIYRVLTILADNNGRIPNKLEFSKELGDDLST

DAIFQFRYFSARTDAKGNVTSFDSRNIFEVSDRQIKNYAKRIVSQNSHSGHITYNFSTYSYLLKKVGKND

YLVVFLDTTNQYLDNQRLLQLSIWMSLVSFIVFMVIVSVLSGRVILPFVANYEKQRRFITNAGHELKTPL

AIISANNELVEMMSGESEWTKSTNDQIQRLTGLINGMVSLARFEEQPDISMVDLDFSHITKDAAEDFKGP

IIKDGKDFIMSIQPGIHVKAEEKSLFELVTLLVDNANKYCDPMGTVTVKLSRSSRLRRAKLEVSNTYKNG

KDIDYSKFFERFYREDESHNNKKSGYGIGLSIVTSLVHLFKGSIDVNYKHDTITFVIYI

>gi|24379373|ref|NP_721328.1| putative histidine kinase [Streptococcus mutans UA159] SMU.928 smut430

MSMFRKIRFRFILVASFAILCILISFVGVFNSVRYVQNNDEINAILTILSRNKGEFPDPIEINKEVGNRG

LSVAVINQYQYYSFIVDRKKDTVSLNLSHISHLTEEQAESYMRNINLHERDGRFRKNNHFYSYRVTKMNP

SGRYLVVVLDATRFFADRSELINLSIQMSFYSLLFFILVVSVFSGYAIKPYLENYEKQKRFITNAGHELK

TPLSIISANNELQELMTGETEWTKSTKDQVERLTHLINQLVTLARLEEQPNIVLERVCFSEIVQDAAEDF

KAPIIKDGKTFEMQIASDIYVKAEEKSLFELVTLLVDNANKYCDDKGTVSVKLSRTSGLKKARLEVSNTF

KEGTEIDYSRFFDRFYREDESHNNQTSGYGIGLSMAQSIVKIFKGKISVSYKNDAITFKVIL

>gi|89893959|ref|YP_517446.1| hypothetical protein DSY1213 [Desulfitobacterium hafniense Y51] DSY1213 dhaf430

MVKNLQKRFIAIAMLSLLVVMVVVLGSLNLVNVGHVTQNADNLLATLAENEGRFPPLGHGEPPRREPRSG

FRMTVETPFETRYFLIRTNKDQSITEIDTSHIAGVSSTEAMDYAQKLLKEGRVNGYLGQYRYLIAEKDYG

NLLILMDCSRQINTSKDFLWNSIGIALISLLILFILVSALSRRAIRPVIENMERQKQFITDAGHEIKTPL

AIIAANSEVLELTNGKNEWTQSIQHQVKRLDELVKNLLTLAKMDEGKVELIFENLSFSDLVEETAGSFTA

LGEKKGLEFVLDIQPGLLIKGEESSLRYLVTILVDNGVKYAAEHGQLKVSLKKRGKTTCLEVYNTCDPLP

EGDLNRLFDRFARGETSRSRESGGYGIGLSVAQAIVTAHQGRITVERREQGISFMVVI
