# Supplementary material for: Evolutionary history of the OmpR/IIIA family of signal transduction two component systems in Lactobacillaceae and Leuconostocaceae
Source: BMC Evol Biol. 2011 Feb 1;11:34. doi: 10.1186/1471-2148-11-34 (PMC3040137; doi:10.1186/1471-2148-11-34)
Supplement: Additional file 2 — Alignments. A zip file containing the alignments used in this study in either FASTA or Phylip format. Details of the sequences used in this study and the tags used to identify them in the alignment files can be found in the files IIIA-seqs.doc and OmpR-seqs.doc (MS Word). A detailed list of the alignments can be found in the file readme.doc (MS Word). [file 1471-2148-11-34-S2.ZIP › alignments/OmpR-seqs.doc]

>gi|116493790|ref|YP_805524.1| DNA-binding response regulator, OmpR family (Rec-wHTH domains) [Lactobacillus casei ATCC 334] lsei_0219 lsei219

MKILIVDDDKEIVELLSIYVKNEGYEPIQAYSGKEAITKLVTNPDIALMILDIMMPNMSGMDVVKEVRKD

SQIPIIIVSAKTADMDKIQGLITGADDYVTKPFNPLEVMARVKSLLRRSQNEVTNTEPDVLEGGPLTIKK

DSHEVTTLTGKQIQLTALEFGILYLLASHPNRVFSADEIFERVWQQESIVSAKTVMVHVSHLRDKIEEAT

DGEKVIETVWGVGYKVEA

>gi|116493445|ref|YP_805180.1| DNA-binding response regulator, OmpR family (Rec-wHTH domains) [Pediococcus pentosaceus ATCC 25745] PEPE_1717 pepe219

MKILVVDDDKEIVELLDIYIKNEGYEPVSAYNGKEALSKLRTDPDISLMILDIMMPEMDGMQVVREVRKD

SSLPILMLSAKTDDMDKIKGLLTGADDYVAKPFNPLEVMARVKSLLRRSEKDMTADEPEILDIGSLIINS

DSHEVRTLAGKDVQLTALEFGILYLLASHPNRVFSADEIFERVWQQESVISAKTVMVHVSHLRDKIEEAT

GGEKVIETVWGVGYKVVAH

>gi|116490235|ref|YP_809779.1| DNA-binding response regulator, OmpR family (Rec-wHTH domains) [Oenococcus oeni PSU-1] OEOE_0119 oeoe219

MTKILVVDDDKEIAELLEIYIRNEGNEPVLAYSGEEALSRLNTNPDIALMILDVMMPGMNGFDVVKAVRK

DTNIPILIVSAKTGDMDKIQGLITGADDYVTKPFNPLEVMARVRSLLRRSSNEVKDEIPEVIDAGALTIN

KDSHEVKTSDGEIVQLTALEFGILYLLASHPNRVFSADEIFERVWQTESVVSAKTVMVHVSHLRDKIKEA

TNGDEVIQTVWGVGYKVEIN

>gi|161508040|ref|YP_001578007.1| Two-component response regulator [Lactobacillus helveticus DPC 4571] lhv_1855 lhel219

MKILVVDDDKEIVELLSIYLKNEGYEPIAAYSGKEAITKLTTTPDIALMILDIMMPNMSGIEVIKEVRKD

SDIPIIIVSAKTGDMDKIQGLITGADDYVSKPFNPLEVMARVRSLLRRSQKQVKDDEPDVLEVGPLVINR

DSHEVKTIDGKAIQLTALEFGILYLLASHPNRVFSADEIFERVWQQESIISAKTVMVHVSHLRDKIQKAT

GGEDVIQTVWGVGYKVEA

>gi|116617476|ref|YP_817847.1| DNA-binding response regulator, OmpR family (Rec-wHTH domains) [Leuconostoc mesenteroides subsp. mesenteroides ATCC 8293] LEUM_0351 leuc219

MKILVVDDDIEIAELLEIYLKNEGYEPIMADDGKEALSKLNTNPDIALMILDVMMPNMNGLDVVKTVRKD

SRIPILMLSAKSGDMDKIQGLITGADDYVTKPFNPLEVMARVRSLLRRAENAVQDQTPDVLDIGSLIINK

DSHEVKTSTGDLVNLTALEFGILYLLASHPNRVFSADDIFERVWQQESVVSAKTVMVHVSHLRDKIEEAT

NGDQVIQTVWGVGYKIEVNQ

>gi|148543356|ref|YP_001270726.1| two component transcriptional regulator, winged helix family [Lactobacillus reuteri F275] Lreu_0117 lreu219

MKILVVDDDKEIVQLLEIYIRNEGYEPISAYDGKEALTKLNTNPDIGLIILDLMMPEIDGMDVIKRVRKD

SDIPILVLSAKTADMDKIQGLITGADDYVTKPFNPLEVMARVKSLLRRSQGEVTNDQPDILNVGPLLINK

DSHEVKTIKGDVIQLTALEFGILYLLASHPNRVFSADDIFERVWQQESVVSAKTVMVHVSHLRDKIEEAT

NGEKVIQTVWGVGYKVEDH

>gi|90961187|ref|YP_535103.1| Two-component response regulator [Lactobacillus salivarius subsp. salivarius UCC118] LSL_0205 lsal219

MKILIVDDDKDIVELLSIYVENEGYEVEKAYNGKEALSKLNMNPDIALMILDVMMPQMDGMEVIREVRKE

SQIPVLILSAKTDDLDKIQGLVQGADDYVTKPFNPLEVMARVKSLLRRAEHNVKNNELDQIEIGPLIIKK

DSHEVKTLEGKEIKLTALEFGILHLLASHPNRVFSADEIFERVWQQESVVSAKTVMVHVSHLRDKIEDAT

NGEQVIQTVWGVGYKIESH

>gi|42519762|ref|NP_965692.1| two-component system response regulator [Lactobacillus johnsonii NCC 533] LJ0563 ljoh219

MKILVVDDDKEIVELLSIYLKNEGYEPIAAYSGKEALTKLSTNPEIALMILDIMMPQMSGIEVIKEVRKD

SEIPILVVSAKTSDMDKIQGLITGADDYVVKPFNPLEVMARVRSLLRRSQKEVKDDKPDVLEVGPLVINK

DSHEVKTLTGKVIQLTALEFGILYMLASHPNRVFSADEIFERVWQQESVVSAKTVMVHVSHLRDKIQKAT

DGEEVIQTVWGVGYKVEA

>gi|116630289|ref|YP_815511.1| DNA-binding response regulator, OmpR family (Rec-wHTH domains) [Lactobacillus gasseri ATCC 33323] LGAS_1735 lgas219

MKILVVDDDKEIVELLSIYLKNEGYEPIAAYSGKEALTKLSTNPEIALMILDIMMPQMSGIEVIKEVRKD

SEIPILVVSAKTSDMDKIQGLITGADDYVVKPFNPLEVMARVRSLLRRSQKEVKDDKPDVLEVGPLVINK

DSHEVKTLTGKVIQLTALEFGILYMLASHPNRVFSADEIFERVWQQESVVSAKTVMVHVSHLRDKIQKAT

DGEEVIQTVWGVGYKVEA

>gi|104774697|ref|YP_619677.1| Two-component system, response regulator [Lactobacillus delbrueckii subsp. bulgaricus ATCC 11842] Ldb2046 Lactobacillus delbrueckii subsp. bulgaricus ATCC BAA-365 YP_813730 LBUL_1893 lbul219

MKILVVDDDHEIVELLSIYLKNEGYEPIAAYNGKEALTKLSTTPDIALMILDVMMPNMSGIDVVKEVRKD

SDIPIIIVSAKTGDMDKIQGLITGADDYVSKPFNPLEVMARVRSLLRRSQKQVKGEEPDVLEVGPLIINK

DSHEVKTVEGKEIQLTALEFGILYLLASHPNRVFSADEIFERVWQQESIVSAKTVMVHVSHLRDKIHKAT

DGEEVIKTVWGVGYKVEA

>gi|58338068|ref|YP_194653.1| two-component response regulator [Lactobacillus acidophilus NCFM] LBA1820 laci219

MKILVVDDDKEIVELLSIYLKNEGYEPVTAYSGKEAITKLTTTPDIALMILDVMMPNMSGIEVIKEVRKD

SDIPIIIVSAKTGDMDKIQGLITGADDYVSKPFNPLEVMARVRSLLRRSQKQVKDEKPDILEVGPLVINR

DSHEVKTIDGKAIQLTALEFGILYLLASHPNRVFSADEIFERVWQQESIVSAKTVMVHVSHLRDKIQKAT

DGEDVIQTVWGVGYKVEA

>gi|28379577|ref|NP_786469.1| response regulator [Lactobacillus plantarum WCFS1] lp_3191 lpla219

MKILVVDDDKEIAQLLEIYIKNEGYEPISAYNGREALTKLNTNPDIALMILDIMMPEMDGIEVIKEVRKD

SQLPILVLSAKTGDMDKIQGLITGADDYVTKPFNPLEIMARVKSLLRRSENNVTTTEPDILDIGPLTINK

DSHEVKTLTGKDIQLTALEFGILYLLASHPNRVFSADDIFERVWQQESIVSAKTVMVHVSHLRDKIEEAT

DGEKVIQTVWGVGYKIESR

>gi|116334617|ref|YP_796144.1| DNA-binding response regulator, OmpR family (Rec-wHTH domains) [Lactobacillus brevis ATCC 367] lvis_2057 lvis 219

MKILVVDDDKEIAQLLEIYIKNEGYEPITAYNGKEALTKLHTTPDIALMILDIMMPEMSGIEVVKEVRKD

SQIPILILSAKTGDMDKIQGLITGADDYVTKPFNPLEVMARVKSLLRRSQDQVTDDSPDILDIGTLTINK

DSHEVKTIAGLTISLTALEFGILYLLASHPNRVFSADEIFERVWRQESIVSAKTVMVHVSHLRDKIEEAT

NGEKVIQTVWGVGYKIESH

>gi|81427892|ref|YP_394891.1| Two-component system, response regulator [Lactobacillus sakei subsp. sakei 23K] lsa0277 rrp31 lsak219

MKILIVDDDKEIVELLSIYVKNEGYEPIQAFTGKEALTKIATNPGIDLMILDIMMPNMSGIEVIKAVRKD

SQVPILVVSAKTTDMDKIQGLITGADDYVSKPFNPLEVMARVKSLLRRSQQQVTNEVPDILEVGPLVIKR

DSHEVTTIAGKQIQLTALEFGILYLLASHPNRVFSADDIFERVWKQESIVSAKTVMVHVSHLRDKIEEAT

DGEKVIETVWGVGYKVEV

>gi|184154583|ref|YP_001842923.1| two-component response regulator [Lactobacillus fermentum IFO 3956] LAF_0107 lfer219

MKILIVDDDKEIVQLLEIYARNEGYEPLTAYNGKDAITKLNTMPDISLVILDLMMPEMNGMDVIRQVRKD

SSIPILVLSAKTADMDKIQGLVTGADDYVTKPFNPLEVMARVKSLLRRSENQVTNDKPDVLNVGPLVINK

DSHEVKTLKGDPIQLTALEFGILYLLASHPNRVFSADDIFERVWQQESVVSAKTVMVHVSHLRDKIEAAT

GGEKVIETVWGVGYKVENH

>gi|170016613|ref|YP_001727532.1| response regulator [Leuconostoc citreum KM20] lck_00255 lcit219

MKILVVDDDIEIAELLEIYLKNEGYEPVMAADGKEAISKLNTNPDIGLMILDVMMPNMNGLEVVKIVRKD

SRVPILMLSAKSGDMDKIQGLITGADDYVTKPFNPLEVMARVRSLLRRADNAVQDKTPDVLDIGSLVINK

DSHEVKTANGDIVNLTALEFGILYLLASHPNRVFSADDIFERVWQQESVVSAKTVMVHVSHLRDKIEEAT

NGDQVIQTVWGVGYKIEVN

>gi|16804460|ref|NP_465945.1| hypothetical protein lmo2422 [Listeria monocytogenes EGD-e] LMO2422 lmon219

MTTSILIADDDKEIVDLVKLYLQNEGYTIYQAYDGAEVWRLVQEKQPTLVILDIMMPEMNGLEVCRLMRD

NGILTPILMLSAKAEDNDKIMGLLTGADDYMVKPFNPLELSVRVKAILRRMQQMSQAEPVSQDKVIIGPI

VVDRGLHVVTVGGKELHLTTSEFDILFLLASEPGRVFSSEYIFEKIWQEKALGASKTVMVHISNLRDKLR

EAMGGENVIKTIWGVGYKIEI

>gi|126699228|ref|YP_001088125.1| two-component response regulator [Clostridium difficile 630] CD1624 cdif219a

MNENILIVDDEKEIADLIEVYLKNEGFTVYKFYTGKEALECIESKNLDMAILDIMLPDIDGFHICQKIRE

KYYYPIIMLTAKIEDIDKIMGLTIGADDYITKPFNPLEVVARVKTQLRRYMRYNNSYEQQSIIVNEYDIK

GLIINKETHKCSLFGKEVALTPIEFSILWYLCEHQGKVVPSEELFEAVWGEKYLDNNNTVMAHIGRLREK

IHEPSKNPKFIKTVWGVGYTIEK

>gi|126701227|ref|YP_001090124.1| two-component response regulator [Clostridium difficile 630] CD3600 cdif219b

MNNTVLVVDDEKEIRDLIEIYLLNSGYNVIKAEDGKEALDILKKENIHLMILDIMMPKMDGIEVCRKVRE

SLNIPILMLSAKSEDMDKIQGIMTGADDYLTKPFNPLELTVRVKSLIRRAYYFSGANNEDKDVIKVGVVI

IDKEKHSVTVAGNEIILTSREFDILYLLANNKGRVYSTEEIFEKVWKEKYYQSNNTVMVHMSRIRDKIEK

YTDGEKIIHTVWGVGYKIEK

>gi|116494021|ref|YP_805755.1| DNA-binding response regulator, OmpR family (Rec-wHTH domains) [Lactobacillus casei ATCC 334] lsei_0460 lsei460

MLSDERLRGTVLAVDDDIDILRMLNRYFAARLPTFNLITAESGAGAVQRLTNNPDLILLDVNLPDTDGFS

LAERIRAITSVPIIFLTARVTDEDKVRGLGAGGDDYVTKPFSLAELGARIKAHFRRESQPQKRARMAIFD

QILIDYRAKQVSIGDQVLSLEKKQYQLVELLSLNPGQVFSREQLYEKIWGYDALGDDRVITEHIRRIRLL

FDRYQVTSPIETVWGVGYKWTS

>gi|126698063|ref|YP_001086960.1| two-component response regulator [Clostridium difficile 630] CD0486 cdif460a

MNNKILVVDDEEGIITLLKDYFEMNGYEVYTAQSGKDALKKISKNPDIILLDINMPEIDGIEVCNRIRDY

ISCPILFLTARIENTDKINGFRAGADDYIVKPFDLDELGARVSAHLRREQRKSNQTKIKFYNDLTIDYEK

RLICIYNNQISLSKKEFDIVELLSMNPGQVFDKEKIYDRVWGYNSNGNSDVIMEHIRKIRIKLSKHTLEN

YIETVWGVGYKWIK

>gi|126698058|ref|YP_001086955.1| putative lantibiotic resistance two-component response regulator [Clostridium difficile 630] CD0481 spaR cdif460b

MSKILIIDDEMDLVMLLEDELKAKGHEVLIAYDGQAGIELSKQEPDLIILDIMMPKMNGFEVCQAIRDDV

LCPIIFLSAKQSETDKIKGLTFGGDDYVTKPFGLRELMARIEANLRREKRSQYINEENKRSTLYFGKLCL

YMKERAIKIDGKNIDLTKIEYDIVELLALHAGQVFSREQIYEKVWGCDSEGDSSTVVERVKKIRAKFSAV

TPEKEYISTVWGIGYKWNKM

>gi|56965768|ref|YP_177502.1| two-component response regulator [Bacillus clausii KSM-K16] ABC4010 (assoc. kinase ABC4009 orthologous to LSEI_1042) bkla460

MGAKTVLVVDDEPELLELVVTYLRKESYRALTADNGADALKRIEQDAVDLVILDVMMEGMDGFHVCERIR

QKHSLPVIMLTARGNEADKVRGLKTGADDYMVKPFSPKELMARVEAALRRANMDKIRGRRLVFGELELDQ

DGRAVTVAGSLISLTRREYELLLFLAENAGRAFSREHLFERVWTDTAASSLRTVDTHIKTLRLKLNGAGR

FIETVWGVGYKFGGSE

>gi|116494259|ref|YP_805993.1| DNA-binding response regulator, OmpR family (Rec-wHTH domains) [Lactobacillus casei ATCC 334] LSEI_0711 lsei711

MTVKPRILVIEDDPDLNESITDFIRAFADPEAALNGADGLYLAEEGIFDAIILDIMLPELNGFDVLKKLR

AKQIKTPVLILTAKDALTDKLHGFELGGDDYLTKPFHREELIMRLKALLKRTGHFFDDDGLTAGPFVVHV

TSHEVLINDQPVALNGKEFDLLVYLLQNQKTIITKDQIFDRLWGFDSETAVSVVEVYMSNLRKKLKPLKV

EHWIKTIRNVGYIFQEPEGSE

>gi|116490257|ref|YP_809801.1| DNA-binding response regulator, OmpR family (Rec-wHTH domains) [Oenococcus oeni PSU-1] OEOE_0142 oeoe711

MANSSVKILLIEDDKILNDDITAMLSEIAEVKQVFNGADGEYEAVEAPYDLIVSDLMLPEMNGLDIIKEM

RDAHIETPVLILTAKDSVDDKVKGFEVGADDYLTKPFHREELLARVEALLRRAGIDTEEKEINIGNLKIH

LSNRTVTVGNQNVQLVGKEYDILVYLAKNKNIIITKDQIFDRVWGLDSDTTTSVVNIYLNNLRRKLESVG

KNDLIKTLRNVGFILESDEQN

>gi|116618899|ref|YP_819270.1| DNA-binding response regulator, OmpR family (Rec-wHTH domains) [Leuconostoc mesenteroides subsp. mesenteroides ATCC 8293] LEUM_1810 leum711

MSKAIKILLIEDDVNLADNVVGFLQDFADINAVTDGLDGEFEAQEAPYDLIISDLMLPGETGLELIKNLR

ANDVETPVLILTAKTSLDDKIEGFNVGADDYLTKPFHREELLVRVKALLRRSGVYSEDNTLAVGDVMINL

ENRGVQVHGQPVKLVGKEFDILTYLAQNKNIIVTRDQIFDRVWGIDSDTTINVVNIYLNNLRRKLEAVGQ

NNLIKTLRNIGFILEVEDAERNQ

>gi|116333023|ref|YP_794550.1| DNA-binding response regulator, OmpR family (Rec-wHTH domains) [Lactobacillus brevis ATCC 367] lvis_0354 lvis711

MRTTAKVLVVEDEKALSADIVELIEPICETTTAFDGEQGEFLASQGVFDAIILDLMLPGESGLDLLLHIR

NQGVKTPVLILTAKDTIADKLRGFNDGADDYVTKPFHREELLARMKALLKRTGHLNSSDTIALGQLEINT

SKHLATFNGAPLTLKGREFDLLAYLVGNPGTIITKEQIFNRLWGFDSETSLSVVEVYMSNLRKELKHAGS

DLSLRTIRNAGYIVEAPDEQ

>gi|170017835|ref|YP_001728754.1| response regulator [Leuconostoc citreum KM20] LCK_01486 lcit711

MSKAIKILLIEDDANLADNIVGFLQDFADVNVVTNGLDGEYEGQESPYDLIICDLMLPGENGLEVIKHLR

DNDIETPVLILTAKSSLDDKIEGFNVGADDYLTKPFYREELLVRVKALLRRAGVYSEDNMITVGDVLINL

ENRGVQVHGQPVKLVGKEFDILTYLAQNKNIIVTRDQIFDRVWGIDSDTTINVVNIYLNNLRRKLEAVGQ

NDLIKTLRNIGFILEVDDA

>gi|125623601|ref|YP_001032084.1| two-component system regulator llrF [Lactococcus lactis subsp. cremoris MG1363] llmg_0747 llac711

MIKILLVEDDLSLSKSVYDFLKSFAQVKQVYDGVEGLYEAELGIYDLILLDLMLPEKNGFEVLKELREQN

VDTPVLIMTAKESLDDKMHGFDIGADDYLTKPFYLDELKARIQALLKRTGKLEDSNGLSYGNIRLNLSNK

STLVDDQPVDLIGKEFDLVVYLMQNQNVILPKEQIFDRIWGYDSDTTVTVVEVYMSKIRKKLKDTEFVNN

LSTLRNVGYILR

>gi|24379561|ref|NP_721516.1| putative response regulator CiaR [Streptococcus mutans UA159] SMU.1129 smut711

MIKLLLVEDDLSLSNSIFDFLDDFADVMQVFDGEEGLYEAESGVYDLILLDLMLPEKNGFQVLKELREKG

ITTPVLIMTAKEGLDDKGHGFELGADDYLTKPFYLEELKMRIQALLKRSGKFNENNLTYGELTVDTATNT

TMVNGKEVELLGKEFDLLVYFLQNQNVILPKSQIFDRIWGFDSDTTISVVEVYVSKIRKKLKDTTFGKNL

QTLRSVGYILKDAN

>gi|22537145|ref|NP_687996.1| DNA-binding response regulator CiaR [Streptococcus agalactiae 2603V/R] SAG0985 saga711

MIKILLIEDDLSLSNSVFDFLDDFADVMQIFDGEEGLYEAESGVYDLILLDLMLPEKNGFQVLKELREKG

ITTPVLIMTAKESIDDKGQGFDLGADDYLTKPFYLEELKMRIQALLKRSGKFNDNSLIYGDIRVDMSTNS

TFVNQTEVELLGKEFDLLVYFLQNQNVILPKSQIFDRIWGFDSDTTISVVEVYVSKVRKKLKGTLFSENL

QTLRSVGYILKHVETD

>gi|126699294|ref|YP_001088191.1| two-component response regulator [Clostridium difficile 630] CD1688 cdif711

MKVLIVEDNKILLESVVEELSKHFETEKCEDGEEALYLVNQNIYDLVILDLMLPNINGFDILKKMRVNNI

DTPVLILTAKETLDDKVEAFTIGANDYLTKPFYMEELVARVYAILRTNGKIKERNGLEFKSLYLDTLEKR

VYIEKEEIKLQNKQFNLLEYFVLNKGSILLKEQIYDRIWGIDSDATIEIVEVYVSNLRKKLSKYGYDKYI

KTKRKVGYIFDDK

>gi|116494441|ref|YP_806175.1| DNA-binding response regulator, OmpR family (Rec-wHTH domains) [Lactobacillus casei ATCC 334] lsei_0934 lsei934

MKKILIVDDEPAILTLLQYNLEAEHYQVETATDGQEALDKVRNEPFDFIILDLMLPSLSGLDVTRKIRED

KIQTPIMILTAKDNETDKIVGLELGADDYVTKPFSPREIIARIKAIERRSQSQPQTSQKPGNSDQITVGQ

IAIDPENYKASKGGKRLQLTPKEFELLVYFAQRVGKTLSRDALLNGVWGFDYPAETRMVDIQVSHLRDKI

ETDPKHPDYLKTVRGFGYQMEAPHE

>gi|116492234|ref|YP_803969.1| DNA-binding response regulator, OmpR family (Rec-wHTH domains) [Pediococcus pentosaceus ATCC 25745] PEPE_0431 pepe934

MSKTILVVDDEPAIVTLITYNLEQANYQVVSVDNGLDAVEIVQKGLVDFVIMDLMLPQLDGIEATKQIRK

FDEQIPIIMLTAKTSQVDKILGLELGADDYITKPFSPQELLSRIKVILRRTDRAINLAEIDPTLQAENIA

IDFKRMVVKKNNEKIQLTPNEFKLLRYLYKNANQVLSRDQILEKVWGYEYGGQTRIVDIHISHLRDKLEN

DPKHPQLIKTIRGFGYEFVSKKGD

>gi|116617701|ref|YP_818072.1| DNA-binding response regulator, OmpR family (Rec-wHTH domains) [Leuconostoc mesenteroides subsp. mesenteroides ATCC 8293] LEUM_0585 leum934

MTKILVVDDETAIATLLQYNLQQNGYEVTVASDGLSAYQKAKEQQFNAILLDLMLPEMDGMTVLKQLRQD

KVSTPIILVTAKGDEFDRVLGLELGADDYITKPFSPREVVARLKAVLRRSQVSTDTNKTDEMVISIQDLL

INDSKKTVMKENIVLSLTPREYDLLLYFAQRLGRVIDRETILTAVWGYEYTGESRMVDMHISNLRDKIES

NPKAPKILKTVRGFGYTMTN

>gi|170017613|ref|YP_001728532.1| response regulator [Leuconostoc citreum KM20] LCK_01263 lcit934

MAKILVVDDEIAISTLLKYNLEENGYDVTVASDGQTAYDLAKQESFDAILLDLMLPVIDGMTLLKNLRQD

KVSTPVILVTAKGDEFDRVFGLELGADDYITKPFSPREVVARVKAVLRRSQVDHQVVAESDVIKVQNVVI

DDNKKVVTRDNQILTLTPREYELLLYFAHRIGRVIDRETILTAVWGYEFTGESRMVDMHISNLREKIETN

PKSPQLLKTVRGFGYTLVV

>gi|28377603|ref|NP_784495.1| response regulator [Lactobacillus plantarum WCFS1] lp_0743 lpla934

MTMDKILVVDDEPAIVTLLSYNLKQAGYEVVTATDGADALSLGLAQSFTCILLDLMLPKLDGMEVTKKLR

QEKVQTPIIIVTAKNDEFDKVFGLELGADDYITKPFSPREVLARIKAVIRRTTPDEPVTPPAPMPPANRA

VMLVGDLQIDQNKYRVARNGKNISLTPKEFELLVYFVEREGRVLSRDAILNHVWGYDYASETRIVDIHIS

HLREKIEADPKQPRLIRTVRGFGYEFVGDGHA

>gi|116333280|ref|YP_794807.1| DNA-binding response regulator, OmpR family (Rec-wHTH domains) [Lactobacillus brevis ATCC 367] LVIS_0631 lvis934

MSRVLVVDDEPAIVTLLQYNLEQADYQVVTAIDGEQALNLALHEKFDVILLDLMLPKMDGVEVTKRLRQE

KVKTPIIMITAKTSEFDTVFGLELGADDYITKPFSPREVIARMKAVMRRYHEEEPTDTVPSQQGHRLQVG

DLTIDQDKFQVKRGNQAIELTPKEFELLLFFAKRPGKVWSREQLLEGVWGFDYSGQTRMVDIHVSHLREK

IEQDPKHPTLLKTVRGFGYTFEVQP

>gi|81428113|ref|YP_395112.1| Two-component system, response regulator (possibly involved in phosphate regulation) [Lactobacillus sakei subsp. sakei 23K] lsa0500 rrp2 lsak934

MKTILVVDDEPAILTLLRYNLEQEHFKVMTATDGAQALTMALQQKFDFIILDLMLPKLDGIAVTKKIREA

KLKTPIMILTAKDNETDKIVGLEVGADDYVTKPFSPREIIARIRAIERRTTQPIEQPVEQASETIVVGDL

SVDETEVIAKKGDHKLHLTPKEFELLVYFMHRLNKVQSREKLLNAVWGFDYPAETRMVDIQVSHLREKIE

DNPRHPVYLKTVRGFGYQLEDPAHDQA

>gi|90962157|ref|YP_536073.1| Alkaline phosphatase synthesis two-component response regulator phoP [Lactobacillus salivarius subsp. salivarius UCC118] LSL_1182 lsal934

MAKVLVVDDEQAITTLLKYNLEKAGYEVKTVNNGNDALDEGLTGKYDFIILDLMLPGIDGIEITKRIRQE

KITTYILILTAKDDEYDKIIGLELGADDYLTKPFSPREIVARLKAVKRRVEHYSDNSEKDSKNKYYFGNY

MVNLDSYELRDKNNNLIKLTPKEFELLVYFLRKKGRVLGREELLNNVWGFDYIGQTRMVDMHVSHLREKI

EENPKKPIFLKTVRGFGYRFEDE

>gi|148544779|ref|YP_001272149.1| putative two component transcriptional regulator, winged helix family [Lactobacillus reuteri F275] Lreu_1569 lreu934o

MTKKFLLMSQQDDLVQQLQAICKKEHWTYELVTTPTGLVVALEKNQISGIWWDMTEVSLDTTIATMTLIR

SQVQGPITVFTPRFTEHIQRKLYRAHVDDVIAFPLLLSVFRSLIQHRLWTYHYPQMHSLADDNMTKNDPV

ITTGSWEINQETYTVTKNDKTIALTPKEFQLLSYLVDHKGQVLNRDQLVNGVWGYDILDTSRIVDIHISH

LRDKLEDDSQHPTHLLTVRGFGYKFV

>gi|184155706|ref|YP_001844046.1| two-component response regulator [Lactobacillus fermentum IFO 3956] LAF_1230 lfer934o

MTKDQTFATDLAHFCHHQHWDLDRVTTATALVVNLEQNSYTGILWDLAATNLDTTIATMTLIRHQQNGPL

LVFSPNLDDRQRRKLFRAKVDDVLEKDLAHDVLRPLIEQRCWLYQHATVADEDATTNDDDDEPIIEDRIK

IGDWEIDPKKVRVTKAGQEVSLTRKEFQLLAFLADHQGQVLSREQLLNGVWGYDILGSSRIVDIHVSHLR

DKLEDDSQKPVHLTTVRGFGYKLE

>gi|27468287|ref|NP_764924.1| alkaline phosphatase synthesis transcriptional regulatory protein [Staphylococcus epidermidis ATCC 12228] SE1369 sepi934

MSQKILVVDDEQSIVTLLKYNLETAGYIVEVAYDGEEALKKVETEQPELIVLDVMLPKKDGIEVCKTIRS

DKNLVPILMLTAKDDEFDRVLGLELGADDYMTKPFSPREVVARVKAILRRSQFVNEIEKEDVDDEDIIIG

SIRIRPEFFEVYKEDELLELTPKEFELLLYLIERQGRVITREHMLNSVWNYEFAGDSRIVDVHISHLRDK

LEENPKQPKLIKTVRGLGYKLERPKA

>gi|16804539|ref|NP_466024.1| two-component response phosphate regulator [Listeria monocytogenes EGD-e] LMO2501 lmon934

MVKILVVDDEASIVTLLQFNIEKAGFEVVTAEDGRTGYELALSEKPDLIVLDLMLPEMDGIEVTKKLRQD

KVNVPILMLTAKDEELDKIIGLELGADDYMTKPFSPREVVARIKAILRRTEGKAEIIEELTEDVEATILI

GDLKILPDSYEVYLQDDLLDLTPKEFELLLFLANHRGKVFSRDQLLDTVWNYDYVGETRIVDVHVSHLRD

KIELDTKQPKYIKTIRGFGYKMENVK

>gi|30022664|ref|NP_834295.1| Alkaline phosphatase synthesis two-component response regulator phoP [Bacillus cereus ATCC 14579] BC4589 bcer934

MNNRILVVDDEEFILTLIEFNLQQAGFEVITAMDGEMALQKATTERPDLIILDLMLPKMDGMEVCKELRL

QRVMTPILMLTAKDDEFDKVLGLELGADDYMTKPFSPREVVARVKAILRRTKLQQEEQVAEAPDEDSITI

AELKILPEFYEAYFQGRKLELTPKEFELLVYLAKNKSRVLTRDQLLSAVWNYDFAGDTRIVDVHISHLRD

KIEQNTKKPTYIKTIRGLGYKLEEPKGNE

>gi|116513986|ref|YP_812892.1| DNA-binding response regulator, OmpR family (Rec-wHTH domains) [Lactobacillus delbrueckii subsp. bulgaricus ATCC BAA-365] LBUL_0872 ldel873

MTEIWCVEDDESIREIEMYTLESMNFKVRGFEDGQSFFKALDNSRPDLVILDLMLPDEDGSEILHRLRST

SATEDLPVIIASAKTAEYDKVKNLDSGADDYLTKPFGMMEMVSRVKAVLRRTNRKKEDKIEKDGIKILLK

RHEVFENGHQVDLTLKEYGLLKLLITHPGTVFSREEIMDQIWETGFYGETRTVDVHVRTLRQKLGEAGKH

IETVRGVGYRYHEAD

>gi|22538097|ref|NP_688948.1| phosphate regulon response regulator PhoB [Streptococcus agalactiae 2603V/R] SAG1961 saga873

MIYCVEDDADIREMMLYTLQMAGFKAQGFSSSELFWEAIQEKVPDLILLDIMLPGDDGLTILERLRRKHQ

TEMIPVIMTTAKGSEYDKVKGLDLGADDYLVKPFGMMEMISRIKAVLRRSRQVDSKAHIIIGNLEIDPTN

YWVKRGTEKIHLTLKEFELLVLFFRNPNRVFTRQELLDKVWGEQFLGETRTVDVHIGTLRTKLGEDGYLI

ATVRGVGYRLEERHD

>gi|125974109|ref|YP_001038019.1| two component transcriptional regulator, winged helix family [Clostridium thermocellum ATCC 27405] Cthe_1600 cther873

MIYCVEDDRSIRELIIYALKSNGYEAVGFDKAEPFYKELENKLPDLVLLDIMLPGEDGIEILKKLKASPK

LRNIPVIMLTAKSAEYDKVLGLDSGADDYITKPFGIMEFLSRVKAVLRRSGNVSNSSEISVGQLTMYIDK

HVVIADGKEVALTFKEFELLKYLMENAGIVLTRDKLLEEVWGYEYEGETRTLDVHIRTLRQKLGEAGAII

ETVRGVGYKIGGKA

>gi|116494457|ref|YP_806191.1| DNA-binding response regulator, OmpR family (Rec-wHTH domains) [Lactobacillus casei ATCC 334] LSEI_0950 lsei950

MNYLIVEDEPALGETLRAYLAKDGQAVLATSLAQANERVAQISPDVIILDLSMPDGDGLTWLAQWRKHLT

ARVIVLTANDEELTMLKGLRLADDYVVKPVSLRVLKARIAKQLPMTVQQFGAVSLNLATGQVTKNETAVV

LTAAEYRLLAYFFANPNQILARDQLLAALWDTREQFVADNTLTVTIKRLREKLEDDPAHTTLIRTVRGMG

YFIDG

>gi|153940586|ref|YP_001390235.1| DNA-binding response regulator [Clostridium botulinum F str. Langeland] CLI_0967 cbot950

MNILLVEDDKIIASGIEYSLEQEGFTTILCYDVATAKKALKYNKFDLCIFDLSLPDGNGYELCKIVREKE

DIPIIFLTACDDEVNVVMGLDMGADDYIIKPFRIRELISRIKSVLRRYQKGINSKIILELNDIRINTLDA

KVYKNDKEIVLTALEYRLLLILANNQGQVLSRNQLLQGIWDVAGDFVNDNTLTVYIKRIREKLENDPQKP

VIIKTVRGMGYKVGD

>gi|125973056|ref|YP_001036966.1| two component transcriptional regulator, winged helix family [Clostridium thermocellum ATCC 27405] CTHE_0537 cthe950

MPKILIVEDDKDIVNNLTEYLRDEGFDVDSAGGQNEAINKMDKNKYDLLLVDISLKDGNGFAVCSAAREN

NDIPVIFLSASDDEYNIVTGLEIGADDYIVKPFRPRELLSRIRKALRRNGKAGAVIEHDNIRVDTVRGIV

TKNGSEIFLSALEYRLFLVFLNNKGAVLSRSRLLEEIWNIAGEYVNDNTLTVYIKRLREKIEDDPQNPTF

IKTVRGLGYKVGD

>gi|23097812|ref|NP_691278.1| two-component response regulator [Oceanobacillus iheyensis HTE831] OB0357 oihe950

MKKCRIKEMLETSKVDECNMNILIVEDDKTIATGLEYSLKQEGYDVVVCHKVQEAQRKINNDLDDISLCL

FDLSLPDGSGYELCEMVKRKADIPVIFLTVVDDEVNVVMGLDMGADDYITKPFRIRELISRIKTVLRRYE

KSKPNKEQLSSVISIGSINIHTLEGKVYKETEEILLTALEYRLLMIFVNHRGQVLSRKQLLQLIWDIAGD

FVNDNTLTVYIKRLRKKIEEDAQHPTVIETVRGMGYRMVDTHDKKQ

>gi|116494543|ref|YP_806277.1| DNA-binding response regulator, OmpR family (Rec-wHTH domains) [Lactobacillus casei ATCC 334] lsei_1041 lsei041

MKLLMIEDNTSVAEMMAMFFQKEKWEVVNAYDGEEGLAKFKEAPDSFDIITLDLNLPKMDGMQVAKEIRK

ISQTVPLIMLTARDTESDQVLGLELGADDYVTKPFSPITLIARIKALHRRAEVGAEVGAEQTTSEPPDGF

DVETDHFKLSTKTREAYLDGKQIEGLTPKEFDLLHTLSKSPKQVFSREQLLQLVWDYEYYGDERTVDAHI

KKLRQKVEKVGPQIIQTVWGVGYKFDDSGLDR

>gi|161507256|ref|YP_001577210.1| Response regulator [Lactobacillus helveticus DPC 4571] lhv_0790 lhel041

MSLRILMVEDDNSVAEMMGMFFKKEGWQQDIAVDGVEAVDMFKENADKYDLITLDLNLPKKDGIQVAKEV

RAISPTVPIIMLTARGSESDQVLGLGIGADEYVTKPFSPIALIARIKALHRRVMMEEDVQTQENNNQEYE

IQTAHLKISKDRREVLFDDQLVVDLTPKEFDLLYTMAQKPKQVFSRDQLLDLVWGYEYYGEERTVDAHIK

KLRQKLEKVGAKVIQTVWGVGYKFDDSQVK

>gi|116493100|ref|YP_804835.1| DNA-binding response regulator, OmpR family (Rec-wHTH domains) [Pediococcus pentosaceus ATCC 25745] PEPE_1354 pepe041

MRILMIEDNKSVSDMMGMFFKKERWNVTFVYDGLKAIETFKTDPEQWDIVLLDLNLPGKDGMEVAAELRR

TSQSIPLIMLTARDSESDQVLGLEMGADEYITKPFSPITLIARIKALYRRVSMSRETMTQGAQNAKFDVR

TAHLKISTSTREVYLDEKLISDLTPKEFDLLKTLAQKPMQVFSREQLLQIVWDYEYYGDERTVDAHIKKL

RQKLEAVGPQVVQTVWGVGYKFDDSKIDED

>gi|90962136|ref|YP_536052.1| Two-component response regulator [Lactobacillus salivarius subsp. salivarius UCC118] LSL_1161 lsal041

MKLLMIEDNKSVCEMMSMFFEKEKWDVEYAYDGISAVEKFKEKSDEWDMITLDLNLPGMDGMQVNAEIRK

ISSTVPIIILTARDSESDQILGLEMGADEYVSKPFSPITLIARIKALHRRAQISALGSSKQNKVDDDFDV

KTDHFKLNSSTREAYLNDNRIDGLTPKEFDLLRVLAKKPRQVFSREQLLQLVWDYEYYGDERTVDAHIKK

LRQKIEKVGPQVIQTVWGVGYKFDDTEAE

>gi|42518843|ref|NP_964773.1| two-component system response regulator [Lactobacillus johnsonii NCC 533] LJ0918 ljoh041

MLKILMVEDDNSVAEMMKMFFSKEQWDVDIAKDGVEAVEMFKANPSSYDIVTLDLNLPKKDGMEVAKEIR

AISLSIPIIMLTARDSETDQILGLGIGADEYVTKPFSPLALIARIKALYRRSHLEKDMHASNGIKYDVVT

KHLKISKDRREVRFDDKVVEGLTPKEFDLLYTMAQKPKQVFSRDKLLELVWGYEFFGEERTVDAHIKKLR

QKLEKAGPQVVQTVWGVGYKFDDSEV

>gi|116629893|ref|YP_815065.1| DNA-binding response regulator, OmpR family (Rec-wHTH domains) [Lactobacillus gasseri ATCC 33323] LGAS_1261 lgas041

MLKILMVEDDNSVAEMMKMFFSKEQWEVDIAKDGIEAVEMFKAHPDSYDIVTLDLNLPRKDGMEVAKEIR

AISLSIPIIMLTARDSETDQILGLGIGADEYVTKPFSPLALIARIKALYRRSRIEKDMHASNGVKYDVIT

KHLKISKDRREVRFDDNIVEGLTPKEFDLLYTMAQKPKQVFSRDKLLELVWGYEFFGEERTVDAHIKKLR

QKLEKAGPQVVQTVWGVGYKFDDSEV

>gi|104773776|ref|YP_618756.1| Two-component system, response regulator [Lactobacillus delbrueckii subsp. bulgaricus ATCC 11842] Ldb0688 Lactobacillus delbrueckii subsp. bulgaricus ATCC BAA-365 YP_812688 LBUL_0621 lbul041

MKILMVEDDKSVSQMMELFFKKEGWEQVSAYDGEEAVEIFQTHPRDFDIITLDLNLPKKDGIQVAKEIRR

ISETVPIIMLTARDSESDQVLGLGVGADDYVTKPFSPIALIARIKALHRRVMLEGQPRNLPKNIPSNDFD

LETNHIRISKSRREVYFDENRVEGLTPKEFDLLYTMAQKPKQVFSREQLLELVWGYEYFGEERTVDAHIK

KLRQKLEKSGPQIIQTVWGVGYKFDDSQVNA

>gi|58337060|ref|YP_193645.1| response regulator [Lactobacillus acidophilus NCFM] LBA0746 laci041

MSLNILMVEDDNSVAEMMGMFFKKEGWQQDIAVDGVEAVDMFRKNADKYDLITLDLNLPKKDGIQVAKEV

RAISPTVPIIMLTARGSESDQVLGLGIGADEYVTKPFSPIALIARIKALHRRVMMEEEPDTAKEDKQDYE

ITTKHLKISKNRREVLFDKQPVTNLTPKEFDLLYTMAQKPKQVFSREQLLELVWGYEYYGEERTVDAHIK

KLRQKLEKVGAKVIQTVWGVGYKFDDLQVE

>gi|28379053|ref|NP_785945.1| response regulator [Lactobacillus plantarum WCFS1] lp_2506 lpla041

MKLLMIEDNKSVSEMMAMFFKKEKWDAHFAYDGNEAVEMFNEDVDGWDMVTLDLNLPGKDGMQVSADIRK

ASPTVPIIMLTARDSESDQVLGLEMGADDYVTKPFSPITLIARIKALHRRADLAKTTPADQPASTVSSFD

VQTDHFKLNTKTREAYLADKQIQDLTPKEFDLLKTLAQKPRQVFSREQLLQLVWDYEYYGDERTVDAHIK

KLRQKIEKAGPQIIQTVWGVGYKFDDSGVDAQ

>gi|116333923|ref|YP_795450.1| DNA-binding response regulator, OmpR family (Rec-wHTH domains) [Lactobacillus brevis ATCC 367] lvis_1316 lvis041

MANELFANARGVQPLEVAMKLLMIEDNHSVSQMMSMFFKKEQWDAEFAYDGNEAVEMFSANPNIWDMVTL

DLNLPGMDGMQVSAEIRKLSPTVPIIMLTARDSESDQVLGLEMGADDYVTKPFSPITLIARIKALHRRAE

LGPTAKPTEAEPLNETEAFDVVTENFKLNTKTREAYLNGHQIQDLTPKEFDLLKTLAQKPRQVFSREQLL

QLVWDYEYYGDERTVDAHIKKLRQKIEKVGPQIIQTVWGVGYKFDDSGADQQ

>gi|81428827|ref|YP_395827.1| Two component system, response regulator [Lactobacillus sakei subsp. sakei 23K] lsa1215 rrp48 lsak041

MKILMVEDNKSVSEMMGMFFQKEAWDAHFAYDGNEAVEQFSVDPDSWDIITLDLNLPGMDGMQVAQKIRE

QSKVVPIIMLTARDSESDQVLGLELGADDYVTKPFSPITLIARIKALHRRAELVDEQVGAEDAQVEEVDN

SDYDVQTDHFKMSSKTREAYLMDEPILDLTPKEFDLLKTLAKNPRQVFSREQLLELVWDYQYFGDERTVD

AHIKKLRQKIEEVGPQVIQTVWGVGYKFDDSAVNKH

>gi|16077443|ref|NP_388257.1| hypothetical protein BSU03750 [Bacillus subtilis subsp. subtilis str. 168] BSU03750 yclJ bsub041

MKILMIEDNVSVCTMTEMFFFKEGFEAEFVHDGLEGYQRFTEENWDLIILDIMLPSMDGVTICRKIRETS

TVPIIMLTAKDTESDQVIGFEMGADDYVTKPFSPLTLVARIKAVIRRYKATGKAVIDEDMIETECFTINK

KTREVLLNGEPVENLTPKEFDLLYYLVQNPRQVFSREQLLEQVWGYQFYGDERTVDVHIKRLRKKLASED

KPFLYTVWGVGYKFDED

>gi|16803547|ref|NP_465032.1| hypothetical protein lmo1507 [Listeria monocytogenes EGD-e] lmo1507 lmon041

MKLLMIEDNVSVCEMIEMFFMKEEIDATFVHDGKMGYEAFFKDDYDIAIIDLMLPNMDGMTICRKIREVS

DVPIIILTAKESESDQVLGLEMGADDYVTKPFSPLTLMARIKAVTRRKNSATSTENNEDILETTYFKISK

RTREIFYQDELLDALTPKEFDLLYFLMQHPRQVFSREQLLEQVWGYQFYGDERTVDVHIKRLRQKIATET

KPFLHTVWGVGYKFDETE

>gi|20808919|ref|NP_624090.1| Response regulators consisting of a CheY-like receiver domain and a HTH DNA-binding domain [Thermoanaerobacter tengcongensis MB4] TTE2570 tten041

MGESQKIYVVDDDKNICEIVSLYLEKEGFEVEQIYDGQTALRKIQEKPPKLVILDIMLPGIDGIALCKEV

RKFSKVPIIMLTAKGDTLDKVLGLEIGADDYIVKPFDGKELVARVKAVLRRYEGESEDKSVSYPDLYVSL

SEYKVIYKGKNIDLTPKELELLYFLCTHPNRVFTRDQLLENVWGYDYMGDSRTVDVHIKRLREKVGEGPN

WKLTTVWGVGYKFEVN

>gi|148381483|ref|YP_001256024.1| two-component response regulator [Clostridium botulinum A str. ATCC 3502] CBO3543 cbot041

MEGKIGKVLVVDDDENIAEVIKMYLENSGYDTRVCYNGKEAQEGFLEYKPDLVLLDIMLPHIDGVDVLKW

IRKDSTIPIIMLTAKGDTFDKVLALELGADDYIVKPFEPKELLARVKAVLRRYNVDNENKEALNFEQLTI

DVNSYTVVYKNKEIKMPPKEFELLYYLARNKNRVFTREQLLCEVWGYDYPGDSRTVDVHIKRLREKLEGG

PNWEIETVWGVGYKFEVK

>gi|148543652|ref|YP_001271022.1| two component transcriptional regulator, winged helix family [Lactobacillus reuteri F275] Lreu_0416 lreu041

MEILMIEDNHSVCEMMAMFFKKEKWHCEFAYDGVEAEEKFNADPHKWDIILLDLNLPKKDGMQVAADIRR

VSPTVPLIMLTARDTESDQVLGLEIGADDYVTKPFSTITLIARIKALYRRTHLNKLEDVQDTDSDDTFDI

QTKNFKMNTKTREVFLYGKPVGDLTPKEFDLLKTLASKPRQVFTRSQLLQLVWDYEYYGDERTVDAHIKK

LRQKLENIGPQVIKTVWGVGYKFDDEGND

>gi|184154873|ref|YP_001843213.1| two-component response regulator [Lactobacillus fermentum IFO 3956] laf_0397 lfer041

MKILMIEDNRSVCEMMSMFFKKEKWDATFAYDGEEAVEKFAAEADDWEMVLLDLNLPKKDGMQVAADIRR

ISPTVPIIILTARDSESDQVLGLEMGADDYVTKPFSPLTLIARIKALRRRVQLSGIANKAEQVTSEFDIQ

TSHFKLNSKTREAYLNGNLISNLTPKEFDLLKTLASKPKQVFTREQLLQLVWDYEFYGDERTVDAHIKKL

RQKIEKVGPKVIQTVWGVGYKFDDSEADDK

>gi|116491734|ref|YP_811278.1| DNA-binding response regulator, OmpR family (Rec-wHTH domains) [Oenococcus oeni PSU-1] OEOE_1773 oeoe772

MVKILVVEDDENFNSLVSRYLASNGFEVISCADAQKAYDALYNNLFDLIVTDVMMPGIDGVEFAKTVRQL

NENIPILFVTARDDIKTKKAGFRVGIDDYLVKPIDLDELKLRISAVLRRANISQNQKIEIGNLLLKSEEI

SASINGVPIPLTVREFNILFKMLSFAGRAFSRAELLDDFWDTESNASLRAVDVYITKLREKFKSTNAFEI

KTIHGLGYKTVIRS

>gi|116618064|ref|YP_818435.1| DNA-binding response regulator, OmpR family (Rec-wHTH domains) [Leuconostoc mesenteroides subsp. mesenteroides ATCC 8293] LEUM_0956 leuc772

MANIIVVEDDPNFNRILSIYLSNHGHQVQSALGAQEAYDKMYNQLFDMILSDVMMPNIDGFEFTETVRKI

NPNIPILFVTAKDDIQSKQRGFSSGIDDYMVKPIDMSEMLMRVTALLRRAHVANDRKLTIGNTVLDADSV

SIMVDSQEISVTVREFNILFKLLSYPNKTFSRSQLLDEFWGVDTTTGLRAVDVYITKLRDKFSGSDVFEI

TTVHGLGYKAVLK

>gi|153953197|ref|YP_001393962.1| Transcriptional regulator [Clostridium kluyveri DSM 555] CKL_0560 cklu772

MINILVVEDDIKINHIVCAYLNDNGYHAIGCKTAMEAFDKMMDNIIGIIISDIMMPEMDGYEFAKTVRKQ

NKNIPILFMTARDDISSKKKGFNIGIDDYMVKPIELDELLMRIEALLRRANIASERKLAVGSLTLNADEN

MAYLNGEEVPLTVREFEILFKLLSYPKRTFTRTQLMDEFWGIESESTPRTVDVSITKLRSKLAECKDIEI

VTVRSLGYKVVIKT

>gi|158320237|ref|YP_001512744.1| two component transcriptional regulator, winged helix family [Alkaliphilus oremlandii OhILAs] Clos_1202 aorem772

MFNILVIEDDKNLRRLMEAFLKRENYNVLSAKDGLEALNVLDSFHVDLIISDIMMPHMDGYEFTEQLRKS

NINVPILMVTAKETIEDKKRGFSVGTDDYMVKPINLDEMLLRVAALLRRSNIVNEHRMVIGEVALDYNTL

TVSKKGESIVLPKKEFYLLFKLLSHPKQIFTRQQLMDEIWGMDAETDERTVDVHIKRLREKFGDFSEFEI

ITVRGLGYKAEKLL

>gi|30020095|ref|NP_831726.1| Two-component response regulator [Bacillus cereus ATCC 14579] BC1956 bcer772

MIPKILIVDDDPHIRELVSVFLEREGFQTYEAIDGVDALQKMDDVKVDMVILDIMMPNMDGFDVCFELRK

YYDIPVLMLTAKGETSQKVKGFHLGTDDYLVKPFDPIELVVRVKALLKRYQITVSQSIQVGNVLLNRKTF

EVTSGEQTVTLPLKEFELLFTLGAKSGRTCSREQLIEEIWGYDFEGNERTLDVHINRLREKFQEEQSKFS

IKTIRGLGYRLEVSK

>gi|22536308|ref|NP_687159.1| DNA-binding response regulator [Streptococcus agalactiae 2603V/R] SAG0123 saga772

MIKILVVEDNIVQQKIITTKLTQEGYQFITASNGQEALNCLDTEEVQLIITDIMMPMMDGYQLIQELRSA

AYNVPIIVMTAKSQMEDMTKGFGLGADDYMVKPVQLQELALRIKALLRRANIVAQHQLIIGNTCLNEDEL

SLKYFEQEIIFPQKEFRVLFHLLSYPNRIFTRLELLDSIWGMDTDLDERVVDACINKIRRKVEHLPDFKI

ETVRGVGYRAKND

>gi|116494703|ref|YP_806437.1| DNA-binding response regulator, OmpR family (Rec-wHTH domains) [Lactobacillus casei ATCC 334] LSEI_1209 lsei209

MGKILIVEDHQDIQALLRDVLSPYYAVIQAYDGISALAQFHQEAPDLIILDSMLPNVTGESVLTTIRKTS

SVPVLVLTAIQDKQKTVALLQQGANDYLTKPFDIDELLARVQVQLRQMLPAPSAKAQDQLQVGEITLNLK

RHAVTVNNRPLTLPKKEYAMLALMMRDPHQVFDKQQLYEHVWGEPFMNADNTLNVHISNLRTKINDLAKD

PKYIVSIWGIGVRLI

>gi|28209933|ref|NP_780877.1| transcriptional regulatory protein [Clostridium tetani E88] CTC00163 ctet209a

MVIFIMNSNINILVVEDDTDINNLLCTMLNKQGYKVTSAYSGSEAKMCLEKQDFQLILLDLMIPAISGEN

LISEIRKFKTMPIIVISAKPGQDIKIKVLKLGADDFICKPFDTNEVLARVESQLRRYTVFSSKNKEKNIL

TYKDLSLNKETLEVAVKGEYISLTAREFTILELLMSNQKKVFTKANLFEHAWNEDFFCDHNTVNVHISNI

RSKLAKINPDIEYIHTVWGIGYKINDKT

>gi|28211448|ref|NP_782392.1| two-component response regulator [Clostridium tetani E88] CTC01805 ctet209b

MGVTYMEGLSNANVLIVEDDKEINNLISKALIKEGFSVVQAFEGIEGYSKYRKIDFSIIILDIMLPYLNG

IEIMKKIREESVVPIILISAKDEEYDRIIGLELGADDYIVKPFFVGELVARIKSQLRRYIDFHKNDEVIS

RIQKGDIILDLNNYCIYKNGEKIDLTAKEFGLLKLFFTNPNRVFTKMQIFENVWHEEYRGDDNTIMVNIR

RLRNKIEDDPNNPEYILTVWGIGYKLGDIK

>gi|24379476|ref|NP_721431.1| putative response regulator [Streptococcus mutans UA159] SMU.1038c smut209

MARILQIEDNNDIQKILRDFFSEEHEVIQAYSGTEGLRLFESENIDLVLLDIMLPGKSGEQVLQEIRKVS

SVPIIMLTALSDKQMISQYLLAGANDYIVKPFNLEEVYARVTVQLRSVTKSHNPATTKQTALSIKNITVN

PDTFEISYLEKSVRLGKKEFQILHALLQYPKKIFTKEDLYESVWKEPYLPGDNTLNAQLSNLRRKLAQVD

DSTDYIETIWGLGVRLKGED

>gi|125717269|ref|YP_001034402.1| Two-component response transcriptional regulator (CheY-like receiver and winged-helix DNA-binding domains), putative [Streptococcus sanguinis SK36] SSA_0401 ssan209

MAHILLIEDNSDIHEILKDLFQKEHTVYSAYSGTEGISLFEREEIDLVLLDIMLPGKNGDEVLKEIRSSN

ATVPVVMLTALGEKALVSQYLLNGANDYIVKPFNLDEVFARITVQLRSQQALSGEEEGQSFKNIRLLKAT

FEAVCGEKSVRLSKKEYHILQLLLRHPKKIYTKEELFEQVWEESYLPGDNTLNTHLSNLRKKLAQLDPTE

EYIETVWGLGVRLKED

>gi|15613383|ref|NP_241686.1| two-component response regulator [Bacillus halodurans C-125] BH0820 bhal209

MIEHKILLIEDDVSISEMVKEHLQLEGYHVCCAFDGEEATRLFEQNTYDLVLLDLMLPRRNGIDCLQWIR

TKSVVPVLIMSAKDSDVDKALGLGFGADDYITKPFSMIELTARVKAAIRRTVNYAQASQSAENERIKIHE

LVVELSSFTVKKKGEEIKLTAKEFQILKLFVTHPKKVFTKEQLYRSVWNDDYYSDENVINVHIRRLREKI

EDTPSKPQYIKTIWGIGYRMGEFE

>gi|116495220|ref|YP_806954.1| DNA-binding response regulator, OmpR family (Rec-wHTH domains) [Lactobacillus casei ATCC 334] LSEI_1740 lsei740

MFKIMIVEDDQTISGLIADNLKKWQFDPVVVTDFNAVFDDFLQEKPHLVLLDINLPVFDGYYWVQKIREV

SKVPVIFISSRNTNMDMVMAMNMGADDFLNKPFAMEVLIAKINALLRRAYNYADQGTDVLEHNGLMLNLK

NGMAQIGENEINLSKNEYKLLQILIRQHGQIVSRSRLLKDLWQDERFVDDNTLTVNINRLRKKIEEAGLK

DYIQTKVGQGYIVP

>gi|116494906|ref|YP_806640.1| DNA-binding response regulator, OmpR family (Rec-wHTH domains) [Lactobacillus casei ATCC 334] LSEI_1420 lsei420

MAQKIFIVEDDAVIAKTISDYLNRWAFAVSTVTKFDQVDAEISAAAPDLVIMDISLPYFNGFHWLSELRK

HSKVPVIFLTSSGDDMNLVMAMNLGADDFLAKPIELPVLLAKIQGMLRRTYQYQQIDTNLSHGAFTLVPT

DNQLKTAKQVVALTPTETKLLSLLFSADGEVVSREAMIEKLWEGDDFIDRNALAVNMNRLRKKVTSIGLA

QLIETVKGKGYRLASEGTKP

>gi|81429063|ref|YP_396063.1| Two-component system, response regulator [Lactobacillus sakei subsp. sakei 23K] lsa1454 rrp1 lsak740

MFEIMIVEDDPTIANLIAENLEKWQLKAIIPDDFDTIFDRFLTDKPHLVLLDINLPVYDGFYWCRKIREV

SKVPIIFISSRSTNMDMVMSMNMGGDDFVNKPFSMEVLIAKINALLRRTYNYVDQNTDVIEHNGLLINLQ

SGGAQVGDTVVDLSKNEYKLLQFLMRQHGQIVSREKLLRALWDDERFVDDNTLTVNINRLRKKIEQAGLE

DYIQTKIGQGYIIP

>gi|148544993|ref|YP_001272363.1| two component transcriptional regulator, winged helix family [Lactobacillus reuteri F275] Lreu_1787 lreu740

MAKIFIVEDNPDIIHSTTQELNKWQHDVITVNNWQNIANEILATNPDLVLFDITLPTFDGFYWIQEVRKH

SQVPIIVISAAEIDTNVMHAVAAGADDYIMKPFAMTVLLAKIQALLRRNKQTADAEEIMWDHNSFNPLTN

ILTNENGTAQLTPTEGSMLQVLLSHRNKTVSKEQLLQWLWQGGKYLNENTLNVNISRLRTKLSTIGLRQC

IHTERGLGYRLVENNE

>gi|116617944|ref|YP_818315.1| DNA-binding response regulator, OmpR family (Rec-wHTH domains) [Leuconostoc mesenteroides subsp. mesenteroides ATCC 8293] LEUM_0834 leum740

MKYKIFIVEDNQEIVSLLKKGLISWGLSAVSCKDFNQVEEEITMHSPHLVLMDINLPYYNGFFWTQKIRQ

NSMIPIIFLSSRDEESDMILAMNFGADDYITKPFNIDLLIVKINALLRREYSFSVEKKHSQFQGYTLNII

DNQLIYDSREVVLSKNETKLLYLLFTHPNQLVTREDIMATLWDNEMFVDKNTLAVTLTRLRNKVTEIGFS

KYLQTVKGKGIKLSD

>gi|170016954|ref|YP_001727873.1| response regulator [Leuconostoc citreum KM20] lck_00596 lcit740

MQKVYIIEDDEGITQQMRQALEKWRFAVSSVADFEAIDTEVKQCQPNIIVMDITLPYFDGFFWTQKIRTF

SQVPIIFVSAASLDPNAIRAIAIGADDYLTKPFSTAVFISKIQAILRRIGQYDDFTDTITCGNYSLSIVT

NILQTPTNFIKLTATESIILRLLMLNPNQTISKEKIIKRIWQNGDFTDENILNVNISRLRDKLTQVGLKN

RLTTEYGKGYKFIS

>gi|16803785|ref|NP_465270.1| hypothetical protein lmo1745 [Listeria monocytogenes EGD-e] lmon740

MVKVYIVEDDEVIRDTIRKHLSKWGFEIGVVEDFNNILQEFLAFEPQLVILDVNLPFFDGFYWCNQIREV

SNVPIIFLSSRNSRMDQIMGMNMGADYYIEKPVDLDVLMARINALLRRTYSYADLEEANVMEHNNVFLHI

DTNTLTHLEDKIELTKNEFLILYELMKQKGSIVSRDEIMRALWEDESFVDDNTLTVNVVRIRKKLAEIGL

SEFIKTKKGQGYMIE

>gi|146295565|ref|YP_001179336.1| two component transcriptional regulator, winged helix family [Caldicellulosiruptor saccharolyticus DSM 8903] Csac_0509 csac740

MYKIMIIEDDISIAETIKNHLSKWNFDALYVTDFKNIIECFIQFEPHLVLIDIILPFYNGFYWCNEIRKI

SKVPIMFISSASDNMNIIMAINMGGDDFIEKPFDLNVLTAKVHALIRRTYSFVSNINLIEHKGVILNLNN

TTLIHQNKKIELTKNEYKILQLLMENAGRVVSREEIMQHLWQSDSFIDDNTLTVNITRLRKKLAELGLEN

FIKTKKGIGYIIE

>gi|125718486|ref|YP_001035619.1| Two-component response transcriptional regulator (CheY-like receiver and winged-helix DNA-binding domains), putative [Streptococcus sanguinis SK36] SSA_1685 ssan740

MHKILLVEDDPVIRQMIKKMLEQWGFQVVAVEDFMDVLTIFVREDPHLVLMDIGLPLFNGYHWCQEIRKI

STVPIMFLSSRDQSMDIVMAINMGADDYVTKPFDNNVFLAKVQGLLRRSYEFGNDQSLLEHQGVILNLKS

TDLVFEGKVVTLTKNEFQILRVLFEHSDGIVARDDLMKELWNSDFFIDDNTLSVNVARLRKKLEEHGLKN

FIETKKGIGYGLINGHTG

>gi|55823251|ref|YP_141692.1| response regulator (homolog to RR01Spn & Sth) [Streptococcus thermophilus CNRZ1066] str1336 sthe740

MHKILLVEDDEVIRQQVKKMLEQWGYEVVLVEDFMEVLSIFVKVEPHLVLMDIGLPLFNGYHWCQEIRKV

SKVPIMFLSSRDQAMDIVMAINMGGDDFVTKPFDQNVLLAKIQGLLRRSYEFGKDQNLLEYMGVILNLKA

MDLVYQGEVVSLTKNEFQILQVLFDHAGNIVSREDLMKELWNSDFFIDDNTLSVNVARLRKKLEAVGLKD

FIETKKGVGYGLRHDG

>gi|16080092|ref|NP_390918.1| hypothetical protein BSU30400 [Bacillus subtilis subsp. subtilis str. 168] BSU30400 bsub740

MFKLLLIEDDESLFHEIKDRLTGWSYDVYGIQDFSQVLQEFAAVNPDCVIIDVQLPKFDGFHWCRLIRSR

SNVPILFLSSRDHPADMVMSMQLGADDFIQKPFHFDVLIAKIQAMFRRVHHYNTEPSTIKTWCGAAVDAE

QNLVSNDKGSVELTKNEMFILKQLIEQKNKIVSREELIRSLWNDERFVSDNTLTVNVNRLRKKLDALQLG

AYIETKVGQGYIAKEEDKFYD

>gi|116495160|ref|YP_806894.1| DNA-binding response regulator, OmpR family (Rec-wHTH domains) [Lactobacillus casei ATCC 334] LSEI_1679 lsei679

MSKILIIEDEKNLARFVELELKHEGYETEVHFNGRTGLEAALAEDWDAILLDLMLPELNGLEVCRRVRQV

KNTPIIMMTARDSVIDRVSGLDHGADDYIVKPFAIEELLARLRALLRRISIEGENNTAKQTTIKYRDLVI

EKENRVVRRGDDIIELTKREYELLLTLMENVNVVLARDVLLSKVWGYNSDVETNVVDVYVRYIRNKIDRP

GEPSYIQTVRGTGYVMRS

>gi|81428994|ref|YP_395994.1| Two-component system, response regulator [Lactobacillus sakei subsp. sakei 23K] lsa1384 lsak679

MSRILIIEDEKNLARFVELELKHEGYGTEVHFNGRTGLEAALNEDWDSILLDLMLPELNGLEVCRRVRQV

KNTPIIMMTARDSVIDRVSGLDHGADDYIVKPFAIEELLARLRALLRRIDIEGEHNAAKQTTVTYRDLTV

EKENRIVRRGDDIIELTKREYELLLTLMENVNVVLARDVLLSKVWGYDSDVETNVVDVYIRYLRNKIDRT

GEQSYIQTVRGTGYVMRS

>gi|28378254|ref|NP_785146.1| response regulator [Lactobacillus plantarum WCFS1] LP_1544 lpla679

MSRILIIEDEKNLARFVELELKHEGYDIQVEYNGRKGLDAALAEDFDAILLDLMLPELNGLEVCRRVREV

KNTPIIMMTARDSVIDRVSGLDHGADDYIVKPFAIEELLARLRALLRRIDLESEQQSTKQTTVTYKDLTI

EKENLVVKRGDEVINLTKREYELLLTLMENINVVLARDVLLNKVWGYESEVETNVVDVYIRYLRNKIDRP

GEKSYIQTVRGTGYVIRS

>gi|116333655|ref|YP_795182.1| DNA-binding response regulator, OmpR family (Rec-wHTH domains) [Lactobacillus brevis ATCC 367] lvis_1018 lvis679

MSRILIIEDEKNLARFVELELKHEGYETNVQFNGRTGLEAALSQDFDVILLDLMLPELNGIEVARRVREV

KSTPIIMMTARDSVIDRVSGLDHGADDYIVKPFAIEELLARVRALLRRIHLEGEQKSTKQTTVKFKDLTI

EKENRIVRRGDEVINLTKREYELLLALMENINVVLARDVLLTKVWGYESEVETNVVDVYVRYLRNKIDVP

GQKSYIQTVRGTGYVMRS

>gi|90961498|ref|YP_535414.1| Two-component response regulator [Lactobacillus salivarius UCC118] lsl_0522 lsal679

MSRILIIEDEKNLSRFVELELQHEEYETEVCKNGRQGLELALDEDWDAILLDLMLPELNGLDICRRVRQV

KNTPIIMMTARDSVIDRVSGLDHGADDYIVKPFAIEELLARLRAVLRRVELENEQNGNKQTTLTYRDLTI

EKENRVVRRGDEIIELTKREYELLLTLMENINVVLARDTLLKKVWGYETQIETNVVDVYIRYLRNKIDRP

GEDSYIQTVRGTGYVMRS

>gi|116492482|ref|YP_804217.1| DNA-binding response regulator, OmpR family (Rec-wHTH domains) [Pediococcus pentosaceus ATCC 25745] pepe0714 pepe679

MSRILIIEDEKSLARFVQLELQHEGYEVEVQSNGRTGLEAALSEDFDAILLDLMLPEINGIEVCRRIRRE

KITPIIMMTARDSIIDRVSGLDHGADDYIVKPFAIEELLARLRAVLRRVDLETQDSTFHQTTVEFQDIVI

EKENRVVRRNNEVINLTKREYELLLLLMENINVVQSRETLLNKVWGVDDGVETNVVDVYIRYLRNKIDVK

GRPSYIQTVRGTGYVMRT

>gi|116630026|ref|YP_815198.1| DNA-binding response regulator, OmpR family (Rec-wHTH domains) [Lactobacillus gasseri ATCC 33323] lgas1398 lgas679

MEKIMSKILIIEDEKNLARFVELELRHEKYETQVEGNGRKGLDLALNEDFDAILLDLMLPDLNGLEIARR

VRQEKTTPIIMMTARDSVIDRVSGLDHGADDYIVKPFAIEELLARLRAVLRRVQIEKRAMGDTMGVQKVV

HFNDLTIETANRIVHRGDSTVDLTKREYNLLMTLIENKNNVVTREQLLNKIWGPESKIETNVVEVYVRYL

RNKIDVPGRPSYIKTVRGTGYMVRTDENDEAREEAK

>gi|104774339|ref|YP_619319.1| Two-component system, response regulator [Lactobacillus delbrueckii subsp. bulgaricus ATCC 11842] Ldb1493 Lactobacillus delbrueckii subsp. bulgaricus ATCC BAA-365 YP_813340 LBUL_1389 lbul679

MPKILIVEDEKNLARFVELELQHEGYETVVENNGRKALDRALSEDFDAILLDLMLPDLNGLEIARRVRQS

KTTPIIMMTARDSVIDRVSGLDHGADDYIVKPFAIEELLARLRAVLRRVKIEKDASKNKLPRQKVVKFKD

LTIETANRIVHRGDGKTVDLTKREYNLLMTLIENKNNVVGRDQLLNKIWGPESNIETNVVEVYVRYLRNK

IDIPGQPSYIKTVRGTGYMVRDEEDNA

>gi|42519507|ref|NP_965437.1| two-component regulatory system response regulator [Lactobacillus johnsonii NCC 533] LJ1631 ljoh679

MSKILIIEDEKNLARFVELELRHEKYETQVEGNGRKGLDLALNEDFDAILLDLMLPDLNGLEIARRVRQE

KTTPIIMMTARDSVIDRVSGLDHGADDYIVKPFAIEELLARLRAVLRRVQIEKRAMGDTMGVQKVVHFND

LTIETANRIVHRGDSTVDLTKREYNLLMTLIENKNNVVTREQLLNKIWGPESKIETNVVEVYVRYLRNKI

DVPGRPSYIKTVRGTGYMVRTDENDEAREETK

>gi|148544452|ref|YP_001271822.1| two component transcriptional regulator, winged helix family [Lactobacillus reuteri F275] Lreu_1228 lreu679

MSRILIIEDEENLANFVELELKHEGYDTDVELDGRAGLEAALNQDFDVILLDLMLPELNGIEVARRVREI

KDTPIIIMTARDSVIDRVSGLDHGADDYIVKPFAIEELLARVRALLRRISIEDGNNKEHQTTVNYKDLTI

EKENRVVRRGDEVINLTKREYELLLILMENINVVMSRKDLLSKVWGYDSKVETNVVDVYIRYLRNKIDRP

GEKSYIQTVRGTGYVIRS

>gi|161507847|ref|YP_001577811.1| Two-component system regulator [Lactobacillus helveticus DPC 4571] lhv_1588 lhel679

MAKILIIEDEKNLARFVELELQHENYETVVENNGRKGLEDALNQDFDAILLDLMLPDLNGLEIARRVRQV

KTTPIIMMTARDSVIDRVSGLDHGADDYIVKPFAIEELLARLRAVLRRVKIEKDASRVTVAKQKIVKFKN

LTIETANRIVHRGDGKAIDLTKREYNLLMTLIENKNNVVSRDQLLNKIWGPESNIETNVVEVYVRYLRNK

IDVPGQPSYIKTVRGIGYMVRDEDDDQE

>gi|58337790|ref|YP_194375.1| two-component system regulator [Lactobacillus acidophilus NCFM] LBA1525 laci679

MAKILIIEDEKNLARFVELELQHENYETVVENNGRKGLDDALAQDFDAILLDLMLPDLNGLEIARRVRQV

KTTPIIMMTARDSVIDRVSGLDHGADDYIVKPFAIEELLARLRAVLRRVKIEKDASKVTVAKQKIVKFKD

LTIETANRIVHRGDGKAIDLTKREYNLLMTLIENKNNVVSRDQLLNKIWGPESNIETNVVEVYVRYLRNK

IDAPGQPSYIKTVRGTGYMVRDEDDDQE

>gi|116618821|ref|YP_819192.1| DNA-binding response regulator, OmpR family (Rec-wHTH domains) [Leuconostoc mesenteroides subsp. mesenteroides ATCC 8293] LEUM_1732 leum679

MSKVLIVEDEENLAKFVGLELKHEGYEAETVNDGRSGLDLALENDYDVILLDLMLPELNGLEVARRLREV

KKTPIIMMTARDSVIDRVSGLDYGADDYLVKPFAIEELLARIRSLLRRIAIETETNDKHRSIINFKDLRI

EKENRIARRDDQIINLTKREYDLLLTLVENINVVQSREQLLKEVWGFDSEVETNVVDVYIRYLRNKIDDP

ESKASYIQTVRGTGYVMRS

>gi|170016759|ref|YP_001727678.1| response regulator [Leuconostoc citreum KM20] lck_00401 lcit679

MSKVLIVEDEENLAKFIGLELKHEGYEVETVLDGRSGLDSALENNYDVILLDLMLPELNGLEVARRLRES

KKTPIIMMTARDSVIDRVSGLDYGADDYLVKPFAIEELLARIRSLLRRIAIETESSDKHRAIINFKDLRI

EKENRIARRDDQIINLTKREYDLLLTLVENINVVQSREQLLKEVWGFDSEVETNVVDVYIRYLRNKIDDP

ESKASYIQTVRGTGYVMRS

>gi|184155774|ref|YP_001844114.1| two-component response regulator [Lactobacillus fermentum IFO 3956] laf_1298 lfer679

MSRILIIEDEENLARFVELELKHDGYETDVELDGRQGLDAALNQDFDVILLDLMLPELNGLEVARRVREV

KDTPIIIMTARDSVIDRVSGLDHGADDYIVKPFAIEELLARVRALLRRISIEGDANSVHQTTVTYKDLTI

EKENRVVRRGDEVINLTKREYELLLILMENINVVMSRKELLSKVWGYDSKVETNVVDVYIRYLRNKIDRP

GEKSYIQTVRGTGYVIRS

>gi|125623751|ref|YP_001032234.1| two-component system regulator llrA [Lactococcus lactis subsp. cremoris MG1363] llmg_0908 llac679

MASKKILIIEDEKNLARFVSLELEHEGYATEIKDNGRSGIEEATSKDYDLILLDLMLPELDGFEVARRLR

KEKDTPIIMMTARDSTMDRVAGLDIGADDYITKPFAIEELLARVRAFFRREEHGHAVERAENTSFRDLVI

DKTNRTVHRGKKVIDLTRREYDLLLTLMQNVGDVVTREHLVSQVWGYEEGTETNVVDVYIRYLRNKIDVE

GQDSYIQTVRGLGYVMRERK

>gi|22537765|ref|NP_688616.1| DNA-binding response regulator CsrR [Streptococcus agalactiae 2603V/R] SAG1625 saga679

MGKKILIIEDEKNLARFVSLELLHEGYDVVVETNGREGLDTALEKDFDLILLDLMLPEMDGFEITRRLQA

EKTTYIMMMTARDSVMDIVAGLDRGADDYIVKPFAIEELLARVRAIFRRQEIETKTKEKGDSGSFRDLSL

NTHNRSAMRGDEEISLTKREFDLLNVLMTNMNRVMTREELLEHVWKYDVAAETNVVDVYIRYLRGKIDIP

GRESYIQTVRGMGYVIREK

>gi|56420692|ref|YP_148010.1| two-component response regulator [Geobacillus kaustophilus HTA426] gk2157 gkau679

MNGRILLIEDEAGLARFLELDLKHEGFDVHVCADGREGLELALSEQWDLILLDVMLPRLNGMEVCRRIRA

AKSTPIIMITARDSVFDRVMGLDNGADDYIVKPFAIEELLARIRALFRRVHPEANAQVLTFKDLVVDVQA

RTVKKGDEFIELTKREYDLLVAFMQNINVVLTRDALLDKVWGFDAEVETNVVDVYVRYLRQKLDEHDKER

YIQTVRGTGYVMRP

>gi|15924405|ref|NP_371939.1| truncated (putative response regulator) [Staphylococcus aureus subsp. aureus Mu50] SAV1415 saur679

MTQILIVEDEQNLARFLELELTHENYNVDTEYDGQDGLDKALSHYYDLIILDLMLPSINGLEICRKIRQQ

QSTPIIIITAKSDTYDKVAGLDYGADDYIVKPFDIEELLARIRAILRRQPQKDIIDVNGITIDKNAFKVT

VNGAEIELTKTEYDLLYLLAENKNHVMQREQILNHVWGYNSEVETNVVDVYIRYLRNKLKPYDRDKMIET

VRGVGYVIR

>gi|116496108|ref|YP_807842.1| DNA-binding response regulator, OmpR family (Rec-wHTH domains) [Lactobacillus casei ATCC 334] LSEI_2681 lcasei26

MKSTLLLVEDEAALADSLTTEFELENMSVIWAKDGAEALTLFKNNEGKINVIILDWMLPKLDGFSVLRKI

RQSSQVPIIMLTARDYIGDKVAGLTGGADDYITKPFEMEELVARVEVALRHNRQAVSPATIFQVDDLLVD

TDNKRVQRGEVIMPLTQREYELLVELVAHQDEACSRDDLLAAVWGTDFDGQPNILDVYIRNLRHKIDDHS

GSKKLIHTVRGVGYMLSANVASI

>gi|42519058|ref|NP_964988.1| two-component system response regulator [Lactobacillus johnsonii NCC 533] LJ1132 ljohn26

MEKTKILLIEDEESLASFIQPELELEGYEVVWAKDGKEGLEFFEKEKPTLILLDWMLPVYDGITVLRRIR

KVSDIPIIMLTARNQASDISNALDQGLDDYMTKPFEIEELFARIRVILRRLEREKRRVVSSTLEFSVLKI

DLVSHKFWCKGEEIYLTPKEYALLCELMNDPEKVKSRDELLDIVWGYDFVGQTNTVDVYIRALRNKLGKY

RNLIKTVRGYGYCLRKVDDNE

>gi|58337917|ref|YP_194502.1| response regulator [Lactobacillus acidophilus NCFM] LBA1659 lacido26

MDEKDVKILLVEDEEAVASFVKTELEFEGYQVIWAQDGKEALELFQKEKPTLILLDWMLPVYDGITVLRR

IRKKSEVPIIMLTAKNSTSDISSALDQGLDDYITKPFEIEELFARIRVILRRLEKSNKQKENSTISFNFG

PFKIDLVKHEFFSNDEKIYLTPKEFALMTELMRDPEKVKSRDELLDVVWGYDFVGQTNTVDVYIRTIRNK

IGDPNKKLIQTVRGLGYCLRKAE

>gi|116332987|ref|YP_794514.1| DNA-binding response regulator, OmpR family (Rec-wHTH domains) [Lactobacillus brevis ATCC 367] lvis_0317 lbrev26

MKNTILLVEDEEGLASYVAKELTFEDFDVLTATDGAAAWEIYQQKKDRLLLVLLDWMLPKMDGMEVLRRI

RKHDELPVIMMTARDYLGDKVTGLDNGADDYITKPFEIEELLARIRVIQRRTAHQSGLDQTYHLGDLTLV

TKTRQVDRAGDTIQLTQREYDLLLFLMQHPGQVFTRDELLDNVWGVDFLGQQNVVDVYIGYLRNKIDVAA

APHLFHTVRGVGYSLKEDEAHEH

>gi|28377207|ref|NP_784099.1| response regulator [Lactobacillus plantarum WCFS1] lp_0283 lplan26

MKNTILIVEDEGSLSGYLTKELQFEDFNVLVAANGLAAMETYQAHQNELLLILLDWMLPKLDGMEVLRRI

RKHDQVPVIIMTARDYVGDKVAGLDNGADDYITKPFEIEELLARIRVIQRRIENQVEPNQVYQLDDVQLD

TKAHRVTNGQEEVTLTHREYDLLQFFLAHPDEVFTRDDLLDQVWGADFLGQQNVVDVYVGYLRNKITTTG

ASPLIQTVRGVGYKLREPMSDAQK

>gi|116490563|ref|YP_810107.1| DNA-binding response regulator, OmpR family (Rec-wHTH domains) [Oenococcus oeni PSU-1] OEOE_0488 oeoe26

MVKPIILIIEDETAIVAYLQTELKFEDYIVLTASDGEMGLSVFEQNSNRINVVLLDWMLPKRDGLEVLRR

IRKLNSQVSIILMTAKSDIGDKVAALDSGADDYITKPFEIEELLARLRVTIRHQNKPRPKLYQVSNLVLD

LDAHRVTRDGQVIDLTQREFQLLTYLIENADKTLTRDDLLDNVWGVDFNGQYNTADVYIRYLRQKIDDHF

YPKLIHTVRSVGYVLRAK

>gi|125624324|ref|YP_001032807.1| two-component system regulator llrE [Lactococcus lactis subsp. cremoris MG1363] llmg_1520 llact26

MTYHVLIVEDEPSISDYVKKELVFEDYQVSTAFDGLAALEVFEQAKPAVDVVLLDWMIPKLDGLTVLRRM

KKIQPNTPIIFLTARDYIGDKVAGLDAGADDYITKPFEIEELLARLRLIFRKWSTSRSKVYELDYLKVDL

TAHTTEVAGKTVNLTQREFALLSYFLEHRGIVVSRADILDDVWGMDFDGQENTVDAYVRYLRATIDLPNH

KSLIETVRGVGYRLNGEG

>gi|20807496|ref|NP_622667.1| Response regulators consisting of a CheY-like receiver domain and a HTH DNA-binding domain [Thermoanaerobacter tengcongensis MB4] TTE1016 tteng26

MKEKILIVEDERQIARFLQLEFEHEGYEVKIVYDGTSGVKEALEGDYDLVLLDIMLPGMDGYEVLKKIRE

NSDIPVIMLTAKYEVKDKVRGLDIGADDYVTKPFSIEELFARVRAALRKRNPSIKKDVLKFAGIVMDLTT

HEVRRQGVKIDLTKKEFDLLEFLMRNANIALSREKILRSVWGYDYYGDTNVVDVYIRYLRSKIDDPFDRK

LIHTIRGVGYSLRDGEDED

>gi|16078390|ref|NP_389208.1| hypothetical protein BSU13250 [Bacillus subtilis subsp. subtilis str. 168] BSU13250 bsubti26

MEKGHILIVEDEEKIARVLQLELEYEGYSVTIKHNGTEGLDAAAEGGYSLVLLDVMLPGLSGLEVLRRLR

KTDSQTPVILLTARDSIPDKVTGLDIGANDYVTKPFEIEELLARIRAALRQNGTKTEDIGTFLTYDDLRV

NEKTREVRRGDKEVELTPREFDLLVYMLKHPQQVLTREQILSSVWGFDYIGDTNVVDVYIRYIRKKLDYP

YEKQLIHTIRGVGYAIKG

>gi|23098049|ref|NP_691515.1| two-component response regulator [Oceanobacillus iheyensis HTE831] OB0594 oihey26

MAERILIVEDEQKISRVLQLELEYEGYESEVASNGKDALNKIEQENWDLVLLDIMIPQLSGLEVLRRVRR

AENQTPIILLTARDEVHDKVSGLDLGANDYITKPFQIEELLARVRVHLRKVVQAANDNQTLSVADLQVDL

HAYEVTRNGEKIELTPREFNLLVCLLKNKNIVLTREQLIESVWGYDYFGDTNVVDVYIRYLRQKVDKNFE

IPIIQTVRGVGYTIKEDKQ

>gi|116496230|ref|YP_807964.1| DNA-binding response regulator, OmpR family (Rec-wHTH domains) [Lactobacillus casei ATCC 334] lsei_2808 lsei808

MAKKILVVDDEKPISDIVKFNLDKEGYDVVTAYDGEEALKKVEAESPDLILLDLMLPKIDGLEVARQIRK

EHDTPIIMLTAKDSEIDKVLGLELGADDYVTKPFSNRELVARVKANLRRTASSNAASNEEDEANKELEVG

DLTIHPDAYTVSKRGENIELTHREFELLHYLARHLGQVMTREHLLQTVWGYDYFGDVRTVDVTVRRLREK

IEDNPSHPEWLVTRRGVGYYLRNPDAE

>gi|116493525|ref|YP_805260.1| DNA-binding response regulator, OmpR family (Rec-wHTH domains) [Pediococcus pentosaceus ATCC 25745] PEPE_1797 pepe808

MPKILVVDDEKPISDIVKFNLDKEGYDVVTAYDGEEALAQVEEEKPDLILLDLMLPKIDGLEVARQVRAK

HNIPIIMVTAKDSELDKVVGLELGADDYVTKPFSNRELVARVKANLRRQDQLQQDDETVKNNIKIGPLVI

NSDSYSVTRDGMQLDLTHREFELLHYLAQHTGQVMTREHLLQTVWGYDYFGDVRTVDVTVRRLREKIEET

PGNPQILVTRRGVGYYLRNPEND

>gi|116490221|ref|YP_809765.1| DNA-binding response regulator, OmpR family (Rec-wHTH domains) [Oenococcus oeni PSU-1] OEOE_0105 oeoe808

MSKILVVDDEKPISDVIKFNLQKEGYDVVTAFDGREALEVFASENPDLVLLDQMLPEIEGTEVLRQIRSN

SSTPVIIVTAKDSEIDKVVGLEMGADDYVTKPFSNRELVARVKANLRRQDATLPGVDASGDHSQKEIKVA

DLIIRPDAYTVTKNGIDLDLTHREFDLLHYLARRPGQVLTREALLTEVWGFDYFGDVRTVDVTVRRLREK

VEDSPSRPEILMTKRGVGYFIKPDSVH

>gi|161506705|ref|YP_001576655.1| putative response regulator [Lactobacillus helveticus DPC 4571] lhv_0095 lhel808

MPKKILVVDDEKPISDIIKFNLTKEGFDVDTAYDGEEAVKKVDEYDPDLMILDLMLPKKDGLEVAREVRQ

THDMPIIMVTAKDTEIDKVLGLEMGADDYVTKPFSNRELVARVKANLRRRDIVKKAEAANQDDTDKNIKI

GNLVIMPDAYIVEKDGQKIELTHREFELLYYLAQHMGQVMTREHLLQTVWGYDYFGDVRTVDVTVHRLRE

KIEDNPIQPQVLVTRRGVGYYVKQPSEG

>gi|116619023|ref|YP_819394.1| DNA-binding response regulator, OmpR family (Rec-wHTH domains) [Leuconostoc mesenteroides subsp. mesenteroides ATCC 8293] LEUM_1950 leum808

MSKVLVVDDEKPISDIIKFNLTKEGYDVVTAADGQEALDMYNDENPDLVLLDQMLPEVDGIEVLRQIRSK

SDVPVIMVTAKDSEIDKVLGLEMGADDYVTKPFSNRELVARVKANLRSRKSVSQTSEETFNSTEDIHLGD

LVIHPQAYMVSKDGADIELTHREFELLYYLAQHIGQVMTREHLLQQVWGYDYFGDVRTVDVTVRRLREKI

EDNPSHPNWLATRRGVGYYLRPSEE

>gi|148543262|ref|YP_001270632.1| two component transcriptional regulator, winged helix family [Lactobacillus reuteri F275] Lreu_0019 lreu808

MAKKILVVDDEKPISDIIKFNLEKEGYEVVVAYDGEAALEQVEAENPDLIILDLMLPKIDGLEVAKRVRA

KHTMPIIMVTAKDSELDKVLGLELGADDYVTKPFSNRELVARVKANLRRQATLKAPAEEENNTDIQIGDL

TIHPEAYTVTKRGENINLTHREFELLHYLAQHIGQVINREHLLQTVWGYDYFGDVRTVDVTVRRLREKIE

DNPSHPQWLITRRGVGYYLANPDQN

>gi|90961025|ref|YP_534941.1| Two-component response regulator [Lactobacillus salivarius subsp. salivarius UCC118] LSL_0035 lsal808

MKKILVVDDEKPISDIVKFNLTKEGYDVYTAYDGEEALQQVKEVEPDLILLDLMLPKIDGLEVAREVRKT

HDMPIIMVTAKDSEIDKVLGLELGADDYVTKPFSNRELVARVKANLRRQSAVAAKSSAEDDKNSEITVGD

LTIHPEAYTVSKNGQRIELTHREFELLHYLAQHLGQVMTREHLLQTVWGYDYFGDVRTVDVTVRRLREKI

EDNPSRPTWLVTRRGVGYYLRNPEVE

>gi|42518151|ref|NP_964081.1| two-component regulatory system response regulator [Lactobacillus johnsonii NCC 533] LJ0065 ljoh808

MPKKILVVDDEKPISDIIKFNLTKEGFDVDTAYDGEEAVKKVEEYNPDLMILDLMLPKKDGLEVAREVRQ

THDMPIIMVTAKDTEIDKVLGLEMGADDYVTKPFSNRELVARVKANLRRRDLTQKATEDDEDKNITIGNL

VIMPEAYMVEKNGEKIELTHREFELLHYLAQHMGQVMTREHLLQTVWGYDYFGDVRTVDVTVRRLREKIE

DNPSSPTILVTRRGVGYYVKNPSDE

>gi|116628742|ref|YP_813914.1| DNA-binding response regulator, OmpR family (Rec-wHTH domains) [Lactobacillus gasseri ATCC 33323] LGAS_0064 lgas808

MPKKILVVDDEKPISDIIKFNLTKEGFDVDTAYDGEEAVKKVDEYDPDLMILDLMLPKKDGLEVAREVRQ

THDMPIIMVTAKDTEIDKVLGLEMGADDYVTKPFSNRELVARVKANLRRRDLTQKATEDDEDKNITISNL

VIMPEAYMVEKNGEKIELTHREFELLHYLAQHMGQVMTREHLLQTVWGYDYFGDVRTVDVTVRRLREKIE

DNPSSPTILVTRRGVGYYVKNPSDE

>gi|116513324|ref|YP_812230.1| DNA-binding response regulator, OmpR family (Rec-wHTH domains) [Lactobacillus delbrueckii subsp. bulgaricus ATCC BAA-365] LBUL_0111 lbul808

MPKKILVVDDEKPISDIIKFNLIKEGFDVETAYDGEEAVEKVEECNPDLLILDLMLPKKDGLEVAREVRQ

THDMPIIMVTAKGGEIDKVLGLEMGADDYVTKPFSNRELVARVKANLRRRDIVKKAVEVNQQKEKEQESN

DIQIGNLLIKPDAYLVEKNGEKIELTHREFELLYYMAQHMDQVMTREHLLQTVWGYDYFGDVRTVDVTVH

RLREKIEDNPVRPKILVTRRGVGYFVKQQTEE

>gi|58336426|ref|YP_193011.1| putative response regulator [Lactobacillus acidophilus NCFM] LBA0078 laci808

MPKKILVVDDEKPISDIIKFNLTKEGFDVDTAYDGEEAVKKVDEYDPDLMILDLMLPKKDGLEVAREVRQ

THDMPIIMVTAKDTEIDKVLGLEMGADDYVTKPFSNRELVARVKANLRRRDIVKKAEAANQEEPDKNIKI

GNLVIMPDAYIVEKNGKKIELTHREFELLYYLAQHMGQVMTREHLLQTVWGYDYFGDVRTVDVTVHRLRE

KIEDNPIQPQILVTRRGVGYYVKQPSEG

>gi|28377004|ref|NP_783896.1| response regulator [Lactobacillus plantarum WCFS1] lp_0036 lpla808

MKKILVVDDEKPISDIMKFNLTKEGYEVHVAADGEEALQKVDEVHPDLILLDLMLPKMDGLEVARQVRKN

YDMPIIMVTAKDSELDKVLGLELGADDYVTKPFSNRELVARVKANLRRQGTPSAQPAEVEENSDIEIGDL

VIHPEAYMVSKRGENIELTHREFELLHYLAQHIGQVMTREHLLQTVWGYDYFGDVRTVDVTVRRLREKVE

DNPSHPKWLVTRRGVGYYLRNPENE

>gi|116332709|ref|YP_794236.1| DNA-binding response regulator, OmpR family (Rec-wHTH domains) [Lactobacillus brevis ATCC 367] lvis_0029 lvis808

MPKKILVVDDEKPISDIMKFNLEKEGYEVAVAYDGEAAVQAVEEDVPDLVILDLMLPKIDGLEVAREIRK

KHDMPIIMVTAKDSELDKVLGLELGADDYVTKPFSNRELVARVKANLRRQNTAAHAQPEEPENQDIEIGD

LTIHPEAYSVSKRGANIELTHREFELLHYLAQHIGQVMTREHLLQTVWGYDYFGDVRTVDVTVRRLREKI

EDSPSHPTWLVTRRGVGYYLRNPDSDQA

>gi|81427690|ref|YP_394688.1| Two-component system, response regulator [Lactobacillus sakei subsp. sakei 23K] LSA0077 rrp3 lsak808

MAKKILVVDDEKPISDIVKFNLTKEGYDVYTAYDGEEALQQVEEVVPDLILLDLMLPKVDGLEVARQVRK

SHDMPIIMVTAKDSEIDKVIGLELGADDYVTKPFSNRELVARVKANLRRQGSTSQSDKEANEENHEINIG

DLTIHPEAYIVSKRGTKIELTHREFELLHYLAKHIGQVMTREHLLQTVWGYDYFGDVRTVDVTVRRLREK

IEDNPSHPEWLVTRRGVGYYLRNPEQE

>gi|170016543|ref|YP_001727462.1| response regulator [Leuconostoc citreum KM20] lck_00185 lcit808

MSKILVVDDEKPISDIIKFNLTKEGYDVITAADGQEALALFNDEKPDLVLLDQMLPEIDGVEVLRQIRSK

SETPVIMVTAKDSEIDKVLGLEMGADDYVTKPFSNRELVARVKANLRSRKAVSQHSEEATVHNEDIALGD

LTIHPQAYMVSKAGQDIELTHREFELLYYLAQHIGQVMTREHLLQQVWGYDYFGDVRTVDVTVRRLREKI

EDNPSHPNWLATRRGVGYYLRPDAE

>gi|184154491|ref|YP_001842831.1| two-component response regulator [Lactobacillus fermentum IFO 3956] laf_0015 lfer808

MAKKVLVVDDEKPISDIIKFNLEKEGYEVVVAYDGEEALQKVESESPDLIVLDLMLPKIDGLEVAKQVRA

KRSTPIIMVTAKDSELDKVLGLELGADDYVTKPFSNRELVARVKANLRRQDATVSPANDDRTADIKVGDL

TIHPDAYTVTKRGENINLTHREFELLHYLAQHIGQVINREHLLQTVWGYDYFGDVRTVDVTVRRLREKIE

DNPSHPQWLITRRGVGYYLANPNQD

>gi|23100907|ref|NP_694374.1| two-component response regulator [Oceanobacillus iheyensis HTE831] OB3452 oihe808

MSQKILVVDDEQPIADILQFNLEKEGYQVVVANDGDTAIELAESEKPNLILLDIMLPGKDGNEVLREVRK

TQNIPVIMLTAKDAEIDKVLGLELGADDYVTKPFSNRELIARVKANLRREQVPDETVKTTKDIVIGDLIV

HPDAYVVSRKGEEIELTHREFELLHYLARHIGQVMTREHLLETVWGYDYFGDVRTVDVTVRRLREKIEEN

PSNPLWIVTRRGVGYYLRNPEQE

>gi|16802333|ref|NP_463818.1| hypothetical protein lmo0287 [Listeria monocytogenes EGD-e] LMO0287 lmon808

MAEKKILVVDDEKPIADIVKFNLNKEGFDVYCAYDGDEALELVEEVQPDLILLDIMLPGRDGIEVCREVR

KKYDMPIIMVTAKDSEIDKVIGLELGADDYVTKPFSNRELIARVKANLRRHSQVSSSTAEEEENSELEIG

SLIIHPDAYVASKRGETIELTHREFELLHYLAKHMGQVMTREHLLQTVWGYDYFGDVRTVDVTVRRLREK

IEDNPSHPAWLVTRRGVGYYLRNPEQE

>gi|16081093|ref|NP_391921.1| hypothetical protein BSU40410 [Bacillus subtilis subsp. subtilis str. 168] BSU40410 bsub808

MDKKILVVDDEKPIADILEFNLRKEGYEVHCAHDGNEAVEMVEELQPDLILLDIMLPNKDGVEVCREVRK

KYDMPIIMLTAKDSEIDKVIGLEIGADDYVTKPFSTRELLARVKANLRRQLTTAPAEEEPSSNEIHIGSL

VIFPDAYVVSKRDETIELTHREFELLHYLAKHIGQVMTREHLLQTVWGYDYFGDVRTVDVTVRRLREKIE

DNPSHPNWIVTRRGVGYYLRNPEQD

>gi|15923008|ref|NP_370542.1| response regulator [Staphylococcus aureus subsp. aureus Mu50] SAV0018 saur807

MQMARKVVVVDDEKPIADILEFNLKKEGYDVYCAYDGNDAVDLIYEEEPDIVLLDIMLPGRDGMEVCREV

RKKYEMPIIMLTAKDSEIDKVLGLELGADDYVTKPFSTRELIARVKANLRRHYSQPAQDTGNVTNEITIK

DIVIYPDAYSIKKRGEDIELTHREFELFHYLSKHMGQVMTREHLLQTVWGYDYFGDVRTVDVTIRRLREK

IEDDPSHPEYIVTRRGVGYFLQQHE

>gi|58337702|ref|YP_194287.1| two-component response regulator [Lactobacillus acidophilus NCFM] LBA1431 laci430

MKILVAEDEPQLLRVLTVAMQKAGYEVDPVDNGLKAVEHAKKNCYDVIILDIMMPVMDGITALKQIRESG

DKTYILMLTAKAEIDDRVTGLDSGADDYLTKPFSLKELLARLRSKERREEDFTPNELKVGDVSLNVSEQE

LASHNSIRLSGQETQLMNYFLLNEGKELSTDELLNHVWKNDEDANSDVVWIYVSYLRQKLQSIRSSVKIE

GEKGGNYELVE

>gi|42519461|ref|NP_965391.1| two-component system response regulator [Lactobacillus johnsonii NCC 533] LJ1587 ljoh430

MKILLAEDEEQLSRVLVAAMQSVNYEVDPVFNGRDAVELARKNSYDVIILDIMMPVMDGITALKEIRKSG

DKTYVLMLTAKAEVDDRVTGLDSGADDYLTKPFSLKELLARLRSKERRDDQYTPNEVEVGDLALNVAEQE

LVSHNSIRLGSKETQLLNYLMLNEGKEFSTSDLLAHVWKDDEEASEDVVWIYISYLRQKLQSIQSSVQIV

GEKGGSFSLIR

>gi|116629368|ref|YP_814540.1| DNA-binding response regulator, OmpR family (Rec-wHTH domains) [Lactobacillus gasseri ATCC 33323] LGAS_0710 lgas430

MKILLAEDEEQLSRVLVAAMQSVNYDVDAVFNGRDAVELAKKNSYDVIILDIMMPIMDGITALKQIRKSG

DKTYVLMLTAKAEIDDRVTGLDSGADDYLTKPFSLKELLARLRSKERRDDQYTPNEVEVGDLALNVSEQE

LVSHNSIRLGSKETQLLNYLMLNEGKEFSTSDLLAHVWKDEDDANEEVVWIYISYLRQKLQSIQSSVQIV

GEKGGSFSLIR

>gi|116513940|ref|YP_812846.1| DNA-binding response regulator, OmpR family (Rec-wHTH domains) [Lactobacillus delbrueckii subsp. bulgaricus ATCC BAA-365] LBUL_0802 ldel430

MKILLAEDEAQLNRVLTVAMQKSGYDVDSVFNGQEAVDAVKKHPYDVIIMDIMMPVKNGLDAVKEIRSTG

DKTYIMMLTAKSEIDDKVTGLDVGADDYLTKPFSLKELLARLRSKERRSEDYNSTQELNYDNLKLDLNSQ

ELSSNNSISLSKQETGLMRYFLLNPGKAISTEELFNHVWKNEDESEDVVWVYISFLRSKLASIGANVQIS

GEKGGEFCLERA

>gi|148543300|ref|YP_001270670.1| two component transcriptional regulator, winged helix family [Lactobacillus reuteri F275] Lreu_0058 lreu430

MRILVAEDEQQLSHVLSSAMTASGYQVDIANNGQEAVEQAKENAYDVIILDIMMPVKSGLEALKEIRATG

NRTYIMMLTAMGEEDDKVTGLDAGADDYLSKPFSLKELLARLRSRQRRDDSYQVDVLEFGDLTLNGNDQS

LESHNSISLTNRENRLLQYFILNANKELSANELINHVWDENETADQEDLWINICYLRQKLQAIQSQVTIS

GEKTGPFMIAC

>gi|24379372|ref|NP_721327.1| putative response regulator [Streptococcus mutans UA159] SMU.927 smut430

MSLTILLAEDEEQLSRVYTAALKHQGYQVDQVYNGQDAIEKAHENAYDVFILDIMMPIKTGLEALREIRA

SGDHTHAIMLTAMSEIEDKVTGLEAGADDYLTKPISLKELLARLASLERRLDRFTDKVLTFGSVKLDLAQ

QEMVSGNSIRLAGKESKLMAFFMLNPNKSLSTKEIFQHVWAKDDDPEIDEGYVFIYISYLRQKLKSIKAN

LAILGEENGDFTLVRIGG

>gi|22537475|ref|NP_688326.1| DNA-binding response regulator [Streptococcus agalactiae 2603V/R] SAG1328 saga430

MRVLIAEDEEQMSRVLSTAISHQGYVVDVAYDGQTAIDLANQNAYDVMVMDVMMPVKTGIEAVKEIRQSG

NKSHIIMLTAMAEIDDRVTGLDAGADDYLTKPFSLKELLARLRSMSRRLEDFTPNVLSLGRVTLSVGEQE

LQCENTIRLAGKEAKMLAFFMLNHDKELSTQQLFEHVWGADKDQEDVDEGYVFIYVSYLRQKLQAIGANL

EIIGQEGSSYKLSEINGG

>gi|89893958|ref|YP_517445.1| hypothetical protein DSY1212 [Desulfitobacterium hafniense Y51] DSY1212 dhaf430

MEKGRGMMRLLLAEDELELANALTAILKHNNYSVDAVDNGADALDWALAGNYDGILLDIMMPKMNGLEVL

AKLREKGISTPILMLTAKGEIEDRIEGLDHGADDYLTKPFAMGELLARIRAITRRKTEFAPNLLTVGNLQ

LDRANYELSGEKGSIRLGNKEYQMMEMMMSSPNRLISTEQFMERIWGYDAEAEINVVWVYISYLRKKLTA

LGSTVTIKASRGLGYTLEDSHG
