# Supplementary material for: Evolutionary history of the OmpR/IIIA family of signal transduction two component systems in Lactobacillaceae and Leuconostocaceae
Source: BMC Evol Biol. 2011 Feb 1;11:34. doi: 10.1186/1471-2148-11-34 (PMC3040137; doi:10.1186/1471-2148-11-34)
Supplement: Additional file 2 — Alignments. A zip file containing the alignments used in this study in either FASTA or Phylip format. Details of the sequences used in this study and the tags used to identify them in the alignment files can be found in the files IIIA-seqs.doc and OmpR-seqs.doc (MS Word). A detailed list of the alignments can be found in the file readme.doc (MS Word). [file 1471-2148-11-34-S2.ZIP › alignments/readme.doc]

List of files included and description of their contents:

**IIIA-seqs.doc**: MS Word document containing the sequences of IIIA HKs used in this study. The headings include the corresponding locus_tags and the abbreviated names utilized in the alignments.

**OmpR-seqs.doc**: MS Word document containing the sequences of OmpR RRs used in this study. The headings include the corresponding locus_tags and the abbreviated names utilized in the alignments.

**IIIA-muscle.fasta**: Alignment of IIIA sequences.

**IIIA-muscle-gb.fasta**: Alignment of IIIA sequences trimmed with Gblocks.

**OmpR-muscle.fasta**: Alignment of OmpR sequences.

**OmpR-muscle-gb.fasta**: Alignment of OmpR sequences trimmed with Gblocks.

**YCLs-muscle.fasta:** Alignment of IIIA sequences belonging to orthologous clusters Ycl1 and Ycl2.

**YCLs-muscle-gb.fasta:** Alignment of IIIA sequences belonging to orthologous clusters Ycl1 and Ycl2 trimmed with Gblocks.

**IIIA-PHO_872-muscle.fasta:** Alignment of IIIA sequences belonging to orthologous clusters Pho and 872.

**IIIA-PHO_872-muscle-gb.fasta:** Alignment of IIIA sequences belonging to orthologous clusters Pho and 872 trimmed with Gblocks.

**OmpR-PHO_872-muscle.fasta:** Alignment of OmpR sequences belonging to orthologous clusters Pho and 872.

**OmpR-PHO_872-muscle-gb.fasta:** Alignment of OmpR sequences belonging to orthologous clusters Pho and 872 trimmed with Gblocks.

**IIIA-KIN_ETA-muscle.fasta:** Alignment of IIIA sequences belonging to orthologous clusters Kin and Eta.

**IIIA-KIN_ETA-muscle-gb.fasta:** Alignment of IIIA sequences belonging to orthologous clusters Kin and Eta trimmed with Gblocks.

**OmpR-KIN_ETA-muscle.fasta:** Alignment of OmpR sequences belonging to orthologous clusters Kin and Eta.

**OmpR-KIN_ETA-muscle-gb.fasta:** Alignment of OmpR sequences belonging to orthologous clusters Kin and Eta trimmed with Gblocks.

**IIIA-CRO-muscle.fasta:** Alignment of IIIA sequences belonging to orthologous cluster Cro.

**IIIA-CRO-muscle-gb.fasta:** Alignment of IIIA sequences belonging to orthologous cluster Cro trimmed with Gblocks.

**OmpR-CRO-muscle.fasta:** Alignment of OmpR sequences belonging to orthologous cluster Cro.

**OmpR-CRO-muscle-gb.fasta:** Alignment of OmpR sequences belonging to orthologous cluster Cro trimmed with Gblocks.

**Concatenate-CRO.phy:** Concatenated alignment of trimmed IIIA and OmpR Cro sequences (phylip format).

**IIIA-ETA-muscle.fasta:** Alignment of IIIA sequences belonging to orthologous cluster Eta.

**IIIA-ETA-muscle-gb.fasta:** Alignment of IIIA sequences belonging to orthologous cluster Eta trimmed with Gblocks.

**OmpR-ETA-muscle.fasta:** Alignment of OmpR sequences belonging to orthologous cluster Eta.

**OmpR-ETA-muscle-gb.fasta:** Alignment of OmpR sequences belonging to orthologous cluster Eta trimmed with Gblocks.

**IIIA-YYC-muscle.fasta:** Alignment of IIIA sequences belonging to orthologous cluster Yyc.

**IIIA-YYC-muscle-gb.fasta:** Alignment of IIIA sequences belonging to orthologous cluster Yyc trimmed with Gblocks.

**OmpR-YYC-muscle.fasta:** Alignment of OmpR sequences belonging to orthologous cluster Yyc.

**OmpR-YYC-muscle-gb.fasta:** Alignment of OmpR sequences belonging to orthologous cluster Yyc trimmed with Gblocks.

**Concatenate-reference.phy:** Concatenated alignment of trimmed sequences of141 conserved genes used for reference tree (phylip format).
